# Chrysin Protects Against Titanium Particle-Induced Osteolysis by Attenuating Osteoclast Formation and Function by Inhibiting NF-κB and MAPK Signaling

**DOI:** 10.3389/fphar.2022.793087

**Published:** 2022-03-23

**Authors:** Zuoxing Wu, Chen Li, Yu Chen, Qian Liu, Na Li, Xuemei He, Weibin Li, Rong Shen, Li Li, Chenming Wei, Siyuan Shao, Fangsheng Fu, Jiaxin Ding, Xiaochen Sun, Dairong Wang, Guixin Yuan, Yiji Su, Jinmin Zhao, Jiake Xu, Ren Xu, Xin Xu, Feng Xu

**Affiliations:** ^1^ Research Centre for Regenerative Medicine, Guangxi Key Laboratory of Regenerative Medicine, Guangxi Medical University, Nanning, China; ^2^ Fujian Provincial Key Laboratory of Organ and Tissue Regeneration, School of Medicine, Xiamen University, Xiamen, China; ^3^ Xiang’an Hospital, School of Medicine, Xiamen University, Xiamen, China; ^4^ Pharmaceutic College, Guangxi Medical University, Nanning, China; ^5^ Department of Orthopedics, Guilin People’s Hospital, Guilin, China; ^6^ Department of Orthopedics, The Second Affiliated Hospital, Shantou University Medical College, Shantou, China; ^7^ School of Biomedical Sciences, The University of Western Australia, Perth, WA, Australia; ^8^ Department of Orthopedic Surgery, The First Afiliated Hospital of Xiamen University, Xiamen, China; ^9^ Department of Subject Planning, Ninth Peoples Hospital Shanghai, Jiaotong University School of Medicine, Shanghai, China

**Keywords:** osteoclast, chrysin, osteolysis, NF-κB, MAPK

## Abstract

Bone homeostasis only exists when the physical function of osteoblast and osteoclast stays in the balance between bone formation and resorption. Bone resorption occurs when the two processes are uncoupled, shifting the balance in favour of bone resorption. Excessive activation of osteoclasts leads to a range of osteolytic bone diseases including osteoporosis, aseptic prosthesis loosening, rheumatoid arthritis, and osteoarthritis. Receptor activator of nuclear factor kappa-B ligand (RANKL) and its downstream signaling pathways are recognized as key mediators that drive the formation and activation of osteoclastic function. Hence, osteoclast formation and/or its function remain as dominant targets for research and development of agents reaching the treatment towards osteolytic diseases. Chrysin (CHR) is a flavonoid with a wide range of anti-inflammatory and anti-tumor effects. However, its effect on osteoclasts remains unknown. In this study, we found the effects of CHR on inhibiting osteoclast differentiation which were assessed in terms of the number and size of TRAcP positive multinucleated osteoclasts (OCs). Further, the inhibitory effects of CHR on bone resorption and osteoclast fusion of pre-OC were assessed by hydroxyapatite resorption pit assay and F-actin belts staining; respectively. Western blotting analysis of RANKL-induced signaling pathways and immunofluorescence analysis for p65 nuclear translocation in response to RANKL-induced osteoclasts were used to analyze the mechanism of action of CHR affecting osteoclasts. Lastly, the murine calvarial osteolysis model revealed that CHR could protect against particle-induced bone destruction *in vivo*. Collectively, our data strongly suggested that CHR with its promising anti-tumor effects would also be a potential therapeutic agent for osteolytic diseases.

## Introduction

The dynamical homeostasis of bone is coordinated by two individual but inter-coupling processes, osteoclast (OC)-mediated bone resorption and osteoblast-mediated bone formation (Boyle et al., 2003; Kular et al., 2012). Excessive OC bone resorption is associated with many osteolytic diseases, including osteoporosis, aseptic prosthetic loosening, rheumatoid arthritis, and periodontitis (Hadjidakis and Androulakis, 2006; Kim et al., 2013). Total joint replacement (TJA) is a common orthopaedic surgery. The purpose of this surgery is to alleviate joint pain associated with end-stage joint diseases (such as osteoarthritis and osteoporotic fractures), restore joint flexibility, and improve the quality of life of patients. However, aseptic prosthesis loosening caused by inflammatory local osteolysis has always been a common complication of TJA. Complication of aseptic prosthetic loosening resulted in poor life quality of patients when long-time wearing become abrasive and painful (Wooley and Schwarz, 2004; Yunpeng et al., 2013). Traced to its cause, long-term complications of TJA were mainly caused by the aseptic prosthesis loosening by common particle-derived prosthetic materials, including ultra-high molecular weight polyethylene (UHMWPE) and metal biomaterial implants. Although the specific mechanism of prosthetic loosening remains unclear, it is believed that the abrasive wear particles induce local cells to release chemokines and cytokines that activate OC formation resulting in the increase of bone resorption and bone loss around the prosthesis (Anderson et al., 2008).

Macrophage colony-stimulating factor (M-CSF) and receptor activator of nuclear factor-κB ligand (RANKL) have been regarded as the two key cytokines for OC formation (Lacey et al., 1998; Teitelbaum, 2000). M-CSF bridged the RANKL-mediated OC precursor fusion through promoting OC precursor proliferation and survival, and increasing RANK expression (Arai et al., 1999). RANKL belongs to the tumor necrosis factor (TNF) superfamily due to its similar molecular structure and bio-function (Thaler et al., 2016). Various downstream signaling pathways such as NF-κB and MAPKs (p38, JNK1/2, and ERK1/2) received the activation signals from adaptor proteins TNF receptor-associated factors (TRAFs), in particular TRAF6, which being recruited after RANKL binds to its cognate receptor RANK. This leads to the activation of transcription factors in the nucleus c-Fos and NFATc1 to induce the expression of OC-specific genes such as tartrate resistant acid phosphatase (*Acp5*), V-ATPase V0 domain subunit d2 (*Atp6v0d2*), calcitonin receptor (*Ctr*), and matrix metalloproteinase-9 (*Mmp-9*) (Matsuo et al., 2004; Nakashima and Takayanagi, 2015). Therefore, agents that can restrain the differentiation and/or bone resorption function of OCs are prime drug candidates for the protection of local osteolysis disease.

In recent years, natural compounds have demonstrated many beneficial biological effects including anti-oxidative and anti-inflammatory effects, wherein many of them show therapeutic potential in the treatment of a variety of disease conditions such as bone metabolic disorders (Norman, 2002; Wang et al., 2017). For example, the Chinese medicine alkaloid, bulleyaconitine A (BLA) and its analogues, being approved for the treatment of chronic pain and rheumatoid arthritis, are likely to be candidate compounds for treating osteoporosis and osteolytic conditions. BLA takes action in inhibiting OC function and promoting bone mineralization by stimulating osteoblasts (Zhang et al., 2018). With natural compounds being in abundance, novel candidates with therapeutic potency could be developed to protect bone destruction from OC over-activation and to provide new medical options to osteolytic bone diseases. Chrysin (CHR) is a natural flavonoid found in honey and propolis, fruits, vegetables, and certain beverages at low concentrations. It shows a variety of potential clinical applications, and has been reported to exhibit anti-inflammatory (Funakoshitago et al., 2016; Nile et al., 2017), anti-oxidative (Zeinali et al., 2017; Wu et al., 2018), and anti-tumorigenic (Nagasaka et al., 2018; Wang et al., 2018) effects. Furthermore, the inflammation caused by LPS induction could be blocked by CHR through attenuating JNK and NF-κB signaling cascades (Ha et al., 2010), thereby providing a compelling basis for exploring its effects on osteoclastogenesis and bone resorption.

Chrysin (CHR) is a flavonoid compound with a variety of anti-inflammatory and anti-tumor effects. However, the effects of CHR on osteoclasts and LPS-induced inflammatory osteolysis remain unclear. In this study, we aimed to determine the effects of CHR on OC formation and function *in vitro*, and on abrasive osteolysis *in vivo*, and to define the molecular mechanisms by which CHR exerts these effects. The results of this study aim to provide a theoretical basis for the clinical application of CHR in the treatment of osteolysis.

## Materials and Methods

### Regents

**FIGURE 1:**
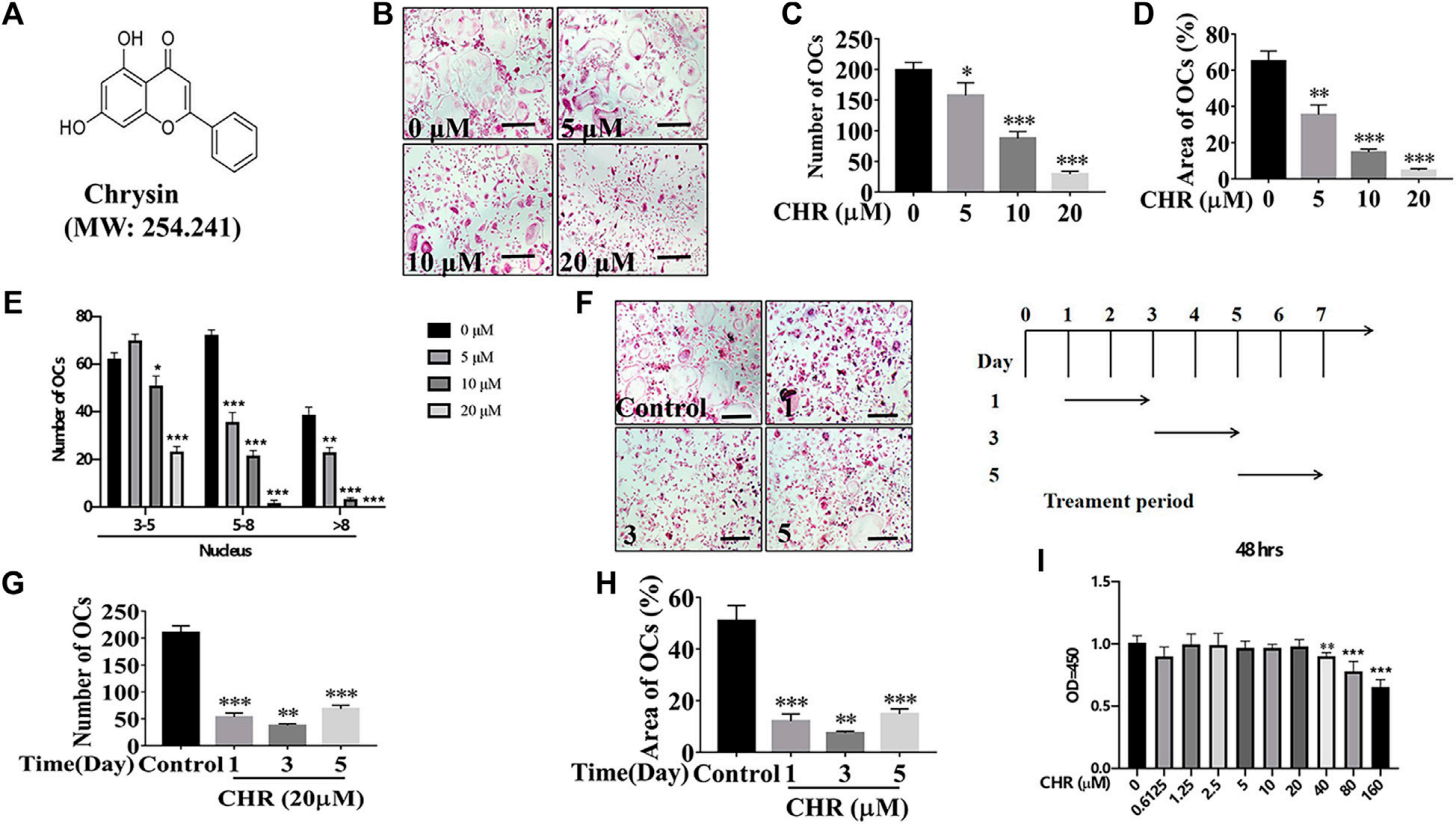
CHR reduced RANKL-induced OC formation. **(A)** Chemical structure of CHR. **(B)** Murine BMMs were dose-dependently treated with indicated concentrations of CHR, 30 ng/ml M-CSF, and 100 ng/ml RANKL for 6 days and then stained for TRAcP activity (Magnification = ×100; scale bar = 100 µM). **(C)** The numbers of TRAcP positive multinucleated OC (nuclei > 3) were quantified (n = 3). **(D)** The percentage of OC area from panel B was measured. **(E)** Quantitative analysis of osteoclast number with different nuclear numbers. **(F)** Murine BMMs were stimulated with 30 ng/ml M-CSF and 100 ng/ml RANKL in the presence of 20 μM CHR for 1, 3, or 5 days. Cells were then fixed and stained for TRAcP activity (Magnification = ×100; scale bar = 100 µM). **(G)** The numbers of TRAcP positive multinucleated OC (nuclei > 3) were quantified (n = 3). **(H)** The percentage of OC area from panel F was measured. **(I)** BMMs were treated with indicated concentrations of CHR and 30 ng/ml M-CSF, for 48 h; Cell viability was measured using CCK-8 assay in accordance with manufacturer’s protocol. (n = 5) (*p < 0.05, **p < 0.01, ***p < 0.001 versus the 0 or positive control).

Chrysin (purity > 98% Figure 1A) was purchased from Aladdin reagent co., LTD (Shanghai, China). Recombinant mice M-CSF was obtained from R&D Systems (Minneapolis, MN, United States). The purification process of Recombinant RANKL followed the protocol as previously reported (Xu et al., 2000). The CCK8 assay kits were purchased from Promega (Madison, WI, United States). Antibodies for ERK, phospho-ERK, IκBα, phospho-IκBα, and β-Actin were obtained from Abcam (Cambridge, United Kingdom). Anti-phospho-JNK, anti-JNK, anti-phospho-p38, anti-p38, anti-phospho-NF-κB p65, and anti-NF-κB p65 were obtained from Cell Signaling Technology (Boston, United States).

### Cell Culture and Cell Viability Assay

The first step was to obtain murine bone marrow monocyte/macrophages (BMMs) that were isolated from 6 to 8 weeks old male C57BL/6 mice weighing about 20 g. Complete medium of a-MEM with supplement of 10% (v/v) FBS 1% (w/v) penicillin/streptomycin, and 30 ng/ml M-CSF was used for cell growth in a humidified incubator at 37°C and 5% CO_2_ cultured until ready for further applications. To assess the cytotoxic effects of CHR on BMMs, cell viability was determined using the CCK8 cytotoxicity assay. BMMs were seeded at a density of 8 × 10^3^ cells/well in 96-well plates and cultured with M-CSF (30 ng/ml) in the absence or presence of CHR (0.625–160 μM) for 48 and 96 h. CCK8 solution (10 μl/well) was added to each well and incubated with cells for 1.5 h. The absorbance at 450 nm was detected by microplate reader (Thermo, United States).

### Osteoclast Formation Assay *In Vitro*

The observation of whether OC differentiation was inhibited under the CHR administration was conducted by tartrate resistant acid phosphatase (TRAcP) activity staining. M-CSF-dependent BMMs were seeded into 96-well plates as 8 × 10^3^ cells/well with complete α-MEM. Cells were placed overnight ensuring the tight adhesion. Stimulation of BMMs using 100 ng/ml RANKL combined with gradually doubling of CHR (from 5, 10 to 20 μM, dose-dependent effect) in every other day. Replacement of culture media containing M-CSF, RANKL, and CHR occurred every 2 days to generate mature multinucleated OCs. Cells were fixed with 4% paraformaldehyde for 15–20 min, followed by the staining of TRAcP activity. Scoring criteria obeyed the rule that the number of TRAcP-positive cells with more than 3 nuclei would be counted as mature osteoclasts.

### Real-Time PCR Analysis

Real-time PCR was used for investigating the expression of specific OC genes at the end of OC formation after the application of CHR. The cell RNA extraction reagents and conditions for the real-time PCR instrumentation were as previously described (Masanori et al., 2010). Table 1 shows the related primer sets.

**TABLE 1:** The primer sets used are as follows.

DC-STAMP	Forward: 5′-CTTGCAACCTAAGGGCAAAG-3′
	Reverse: 5′-TCAACAGCTCTGTCGTGA CC-3′
ACP5	Forward: 5′-TGTGGCCATCTTTATGCT-3′
	Reverse: 5′-GTCATTTCTTTGGGGCTT-3′
CTR	Forward: 5′-TGCAGACAACTCTTGGTTGG
	Reverse: 5′-TCGGTTTCTTCTCCTCTGGA
ATP6V0d2	Forward: 5′-GTGAGACCTTGGAAGACCTGAA-3′
	Reverse: 5′-GAGAAATGTGCTCAGGGGCT-3′
MMP-9	Forward: 5′-CGTGTCTGGAGATTCGACTTGA-3′
	Reverse: 5′-TTGGAAACTCACACGCCAGA-3′
β-actin	Forward: 5ʹ-TCTGCTGGAAGGTGGACAGT-3ʹ
	Reverse: 5′-CCTCTATGCCAACACAGTGC-3ʹ
CTSK	Forward: 5′-GGCCAACTCAAGAAGAAAAC3′
	Reverse: 5′-GTGCTTGCTTCCCTTCTGG-3′

### Resorption Pit Assay

BMMs were initially seeded into 6-well plates at a density of 1 × 10^5^ cells/well under the cultivation of additional 100 ng/ml RANKL and the small osteoclastic cells appeared at the third or fourth day. On day 4, cells were transferred to hydroxyapatite-coated 96-well plates (Corning, United States) and treated with 10 or 20 μM CHR and maintained for an additional 2 days. The mature OCs were removed using 10% sodium hypochlorite for 15 min after osteoclast formation was observed, washed twice with PBS, and dried. Light microscopic images captured for each well and pit area were quantified by ImageJ.

### F-Actin Ring Staining

BMMs were seeded with 8 × 10^3^ cells/well into 96-well plates and cultured in complete α-MEM medium containing M-CSF (30 ng/ml), RANKL (100 ng/ml), and CHR (10 and 20 μM) respectively, ensuring the formation of mature multinucleated OC. On day 6 cells were fixed with 4% paraformaldehyde and then were washed, followed by permeabilization under the condition of 0.1% Triton X-100 with 100 μl volume in each well for 5 min. The 3% BSA in PBS was used to block with Non-specific immuno-reactivity. Cells were washed and incubated with Rhodamine-conjugated phalloidin for 1–2 h to stain actin. Following washes 3 times with PBS, cells were counterstained with DAPI for 5 min. Then the fluorescence microscope was used for obtaining images.

### Western Blot Analysis

BMMs were seeded in 6-well plates and maintained until >90% confluence. Cells were serum-starved for 2 h. The treatment group was treated with 20 μM CHR for 2 h, subsequent by stimulation with RANKL at a concentration of 100 ng/ml in time points (discarding RANKL at 5, 10, 20, 30, or 60 min). To explore the effect of Chrysin on RANKL-induced osteoclast signaling events, nuclear and cytoplasmic proteins were extracted from BMMs after combination of stimulation presence or absence 20 μM CHR for 1, 3, or 5 days together with RANKL. Untreated cells served as control. Total cellular protein from each sample was extracted using RIPA buffer contained with PMSF and phosphatase inhibitor. Next, 20 µg of total cellular protein lysate transfected with nitrocellulose membrane by 10% SDS-PAGE. Subsequently, the 5% BSA in TBST (50 mM Tris, pH 7.6; 150 mM NaCl; and 0.1% Tween-20) were used incubated with membrane for one and a half hours and incubated with primary antibody (1:1000) for 12–15 h at 4°C. Subsequently, the membranes were washed 3 times with TBST, and incubated with the IRDye fluorescent secondary antibody corresponding to the primary antibody for 1 h at room temperature. The immuno-reactive bands were analyzed using The Odyssey Infrared Imaging System (LI-COR).

### Titanium Particle-Induced Murine Calvarial Osteolysis Model *In Vivo*

A total of healthy 24 male C57BL/6 mice were obtained from Guangxi Medical University’s Animal Experiment Center and were randomly assigned into 4 experimental groups wherein each group included 6 mice: Sham group, vehicle group, low-dose CHR group, and high-dose CHR group. After anesthesia with 10% chloral hydrate in the abdomen, 30 mg of titanium particles were embedded under the periosteum located at the middle suture of the mouse skull. No particles were embedded in the Sham group. CHR was injected into the calvaria of mice every other day at 4 (low dose) or 8 mg/kg (high dose) for 14 days. Sham and vehicle groups received same volume PBS injections every 2 days during the 14 days of the experimental period. At the end of the experiment, all experimental mice were sacrificed, calvariae were isolated and fixed in 4% paraformaldehyde for subsequent micro-CT scanning, and histological sectioning as previously reported (Qin et al., 2012).

### Micro-CT, Histology, and Histomorphometric Analysis

The scanned images of whole calvarial were reconstructed and analyzed for the degree of calvarial osteolysis using high-resolution Microcomputed tomography (Skyscan 1176; Skyscan; Aartselaar, Belgium). The analysis was performed using 50 kV, 800 μA, 14.4 μM resolution. After 3D reconstruction, the square ROI around the sagittal suture of the murine calvaria was selected as an analysis of further bone mass parameters. The detail information of this process protocol was based on our previous report (Wu et al., 2019). Structural parameters for the calvarias were measured with the built-in software using the bone volume/total volume (BV/TV), the number of porosity, and the percentage of total porosity as previously reported (Feng et al., 2018).

### Statistical Analysis

Data was illustrated either as means ± standard deviation (SD), or the representative one with all independent triplicates. Statistical analysis among or within groups was conducted by one-way ANOVA tests using SPSS 19.0 software (SPSS Inc., United States). *p < 0.05, **p < 0.01, ***p < 0.001 was regarded as statistical significance.

## Results

### CHR Suppressed RANKL-Induced Osteoclast Differentiation *In Vitro*

To investigate the effect of CHR (Figure 1A) on OC formation, murine BMMs were stimulated with RANKL and M-CSF in the absence or presence of CHR in dose- and time-dependent manners. As shown in Figures 1B–E, we found that various dose of CHR (5, 10, and 20 μM) inhibited the number and size of TRAcP positive multinucleated OCs in a dose dependent way, wherein 20 μM had the most pronounced effect. In order to examine the inhibition of CHR towards OC formation was in the time-dependent manner, murine BMMs stimulated with M-CSF and RANKL were exposed to CHR on day 1, 3, or 5. As with the dose-dependent effect, CHR also time-dependently inhibited the formation of mature TRAcP positive multinucleated OCs (Figures 1F,G) and significantly reduced the size of OC (Figures 1F,H) as compared with the untreated control. Treatment of CHR on day 1 or day 3 showed the most pronounced inhibitory effect (Figures 1F–H). To rule out the possibility of cytotoxicity, we performed CCK-8 viability assay on murine BMMs. As shown in Figure 1I, CHR demonstrated little to no cytotoxicity at concentrations up to 20 μM. Collectively, these findings indicated that CHR inhibited OC formation through a mechanism that abrogates early RANKL-induced signaling cascades.

### CHR Suppressed RANKL-Induced Osteoclast Gene Expression

**FIGURE 2:**
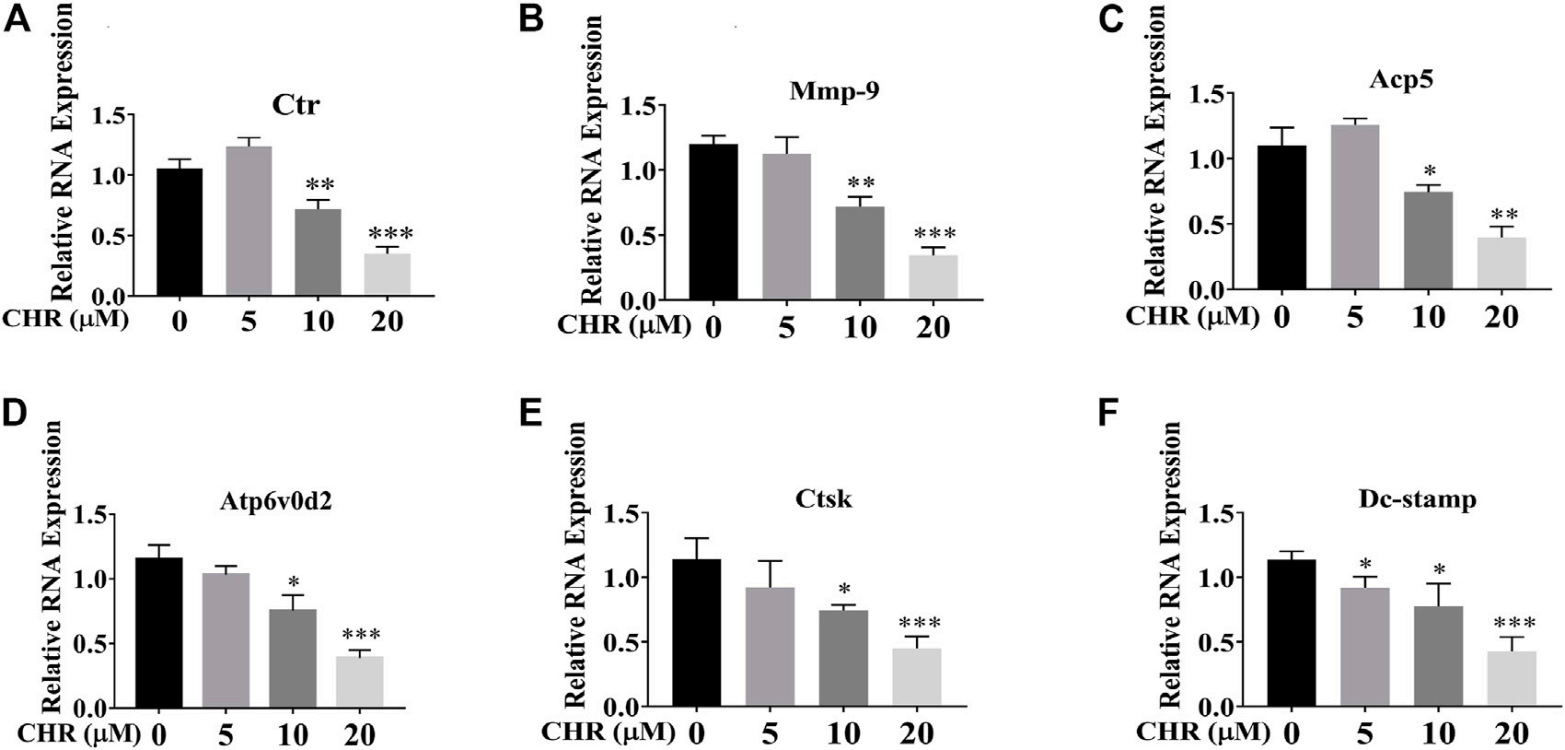
CHR suppressed RANKL-induced expression of OC-associated genes. Murine BMMs were stimulated with 30 ng/ml M-CSF and 100 ng/ml RANKL in the absence or presence of indicated concentrations of CHR for 6 days to form multinucleated TRAcP positive OCs. Cell were lysed, RNA were extracted, and the cDNA generated from reverse transcription were subjected to real-time quantitative PCR using specific primers against OC-specific genes. **(A)** *Ctr,* **(B)** *Mmp-9*, **(C)** *Acp5,* **(D)** *Atp6v0d2,* **(E)** *Ctsk,* and **(F)** *Dc-Stamp*. Gene expression levels of *Ctr, Mmp9, Acp5, Atp6v0d2, Ctsk,* and *Dc-stamp* were expressed relative to control group (n = 3) (*p < 0.05, **p < 0.01, ***p < 0.001).

We next assessed the suppression of CHR on RANKL-induced gene expression during OC formation. Expression levels of OC-related genes after 6-days stimulation of RANKL and M-CSF in the absence or presence of indicated concentrations of CHR were analyzed by real-time quantitative PCR. As shown in Figure 2, treatment with CHR dose-dependently suppressed the expression of genes related to OC formation and activity including *Ctr, Mmp9, Acp5, Atp6v0d2, Ctsk,* and *Dc-stamp* (Phan et al., 2004; Haotian et al., 2009).

### CHR Inhibited Bone Resorption and F-Actin Formation

**FIGURE 3:**
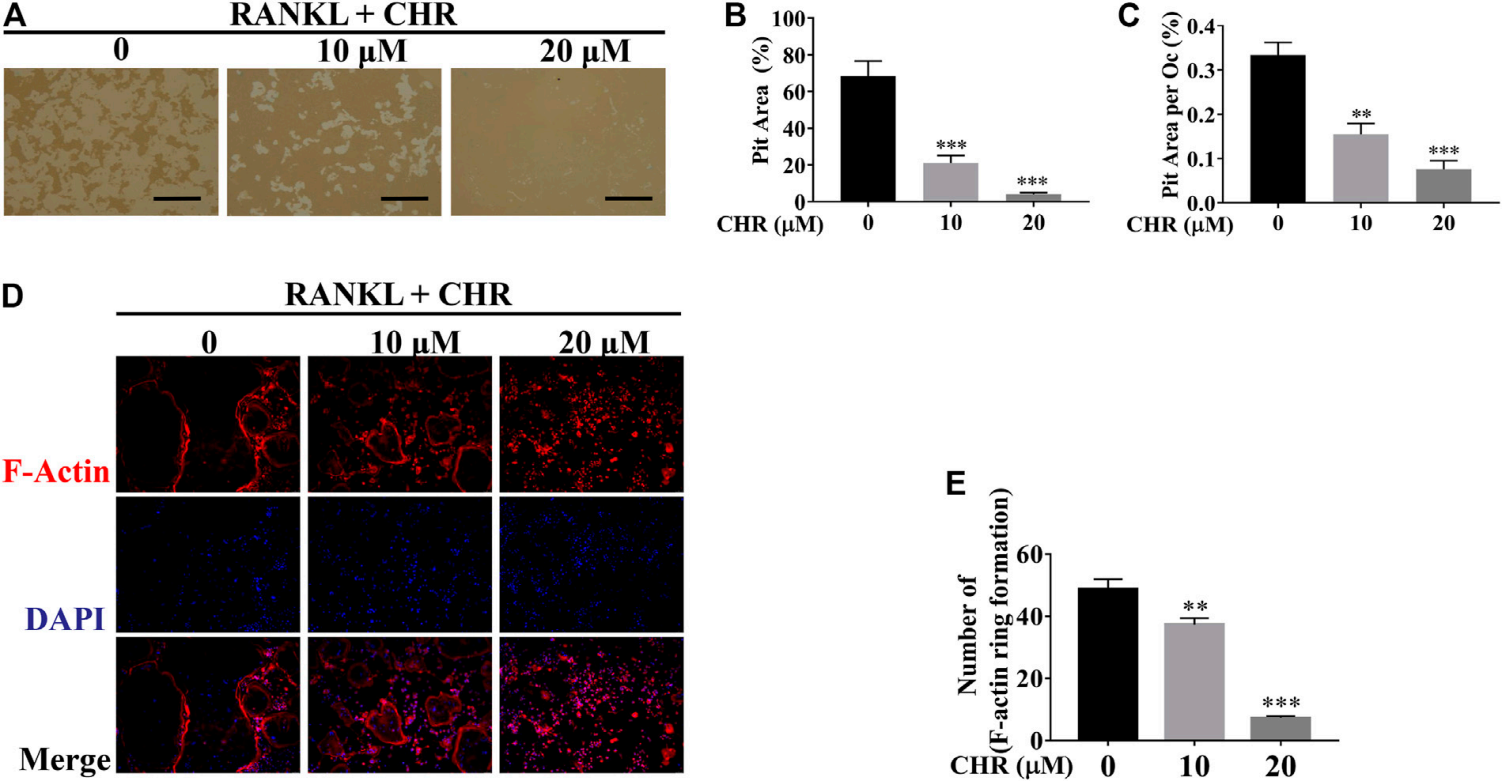
CHR suppressed OC bone resorption and F-actin belts formation. **(A)** Mature OCs were seeded onto hydroxyapatite-coated plates and treated with indicated concentrations of CHR for 48 h. Attached cells were removed and micrographs of bone resorption pits (Magnification = ×100; scale bar = 100 µM). **(B,C)** ImageJ was used to quantify the percentage of absorption pit area occupied by each osteoclast and the total absorption pit area. The data presented is a representative of three independent experiments and expressed as mean ± SD. **(D)** CHR suppressed RANKL-induced F-actin belts formation. Murine BMM cells were stimulated with RANKL and M-CSF and treated with CHR (10 and 20 μM) as previously described. OCs formed were fixed and stained for F-actin and nuclei with Rhodamine-conjugated phalloidin and DAPI, respectively, and images captured using laser scanning confocal microscopy. The data presented is a representative of three independent experiments and expressed as mean ± SD. **(E)** The numbers of OCs based on F-actin belts formation were quantified (**p < 0.01, ***p < 0.001 vs RANKL-treated controls.)

Next, we explored the effect of CHR on the bone resorptive activity of mature OCs. Equal number of mature OCs were seeded into hydroxyapatite coated plates and treated with 10 or 20 μM of CHR for 48 h. Total resorbed areas were quantified by ImageJ. As shown in Figures 3A–C, percentage of average absorption area per osteoclast in CHR treated groups was significantly reduced as compared with positive controls. We next examined F-actin belts formation, a hallmark of actively resorbing OCs, using fluorescence microscopy. Mature OCs treated with indicated concentrations of CHR were stained for F-actin using Rhodamine-conjugated phalloidin and counterstained with DAPI to determine the number for nuclei per OC. As shown in Figures 3D,E, in the presence of CHR, the formation of F-actin rings were significantly reduced as compared with untreated controls, consistent with a reduction in size of the OC demonstrated in earlier experiments. Together, the results suggest that CHR can exert a suppression effect on RANKL-induced OC formation, fusion, and bone resorption in a dose-dependent manner.

### CHR Inhibited RANKL-Induced NF-κB and MAPK Signaling Pathways

**FIGURE 4:**
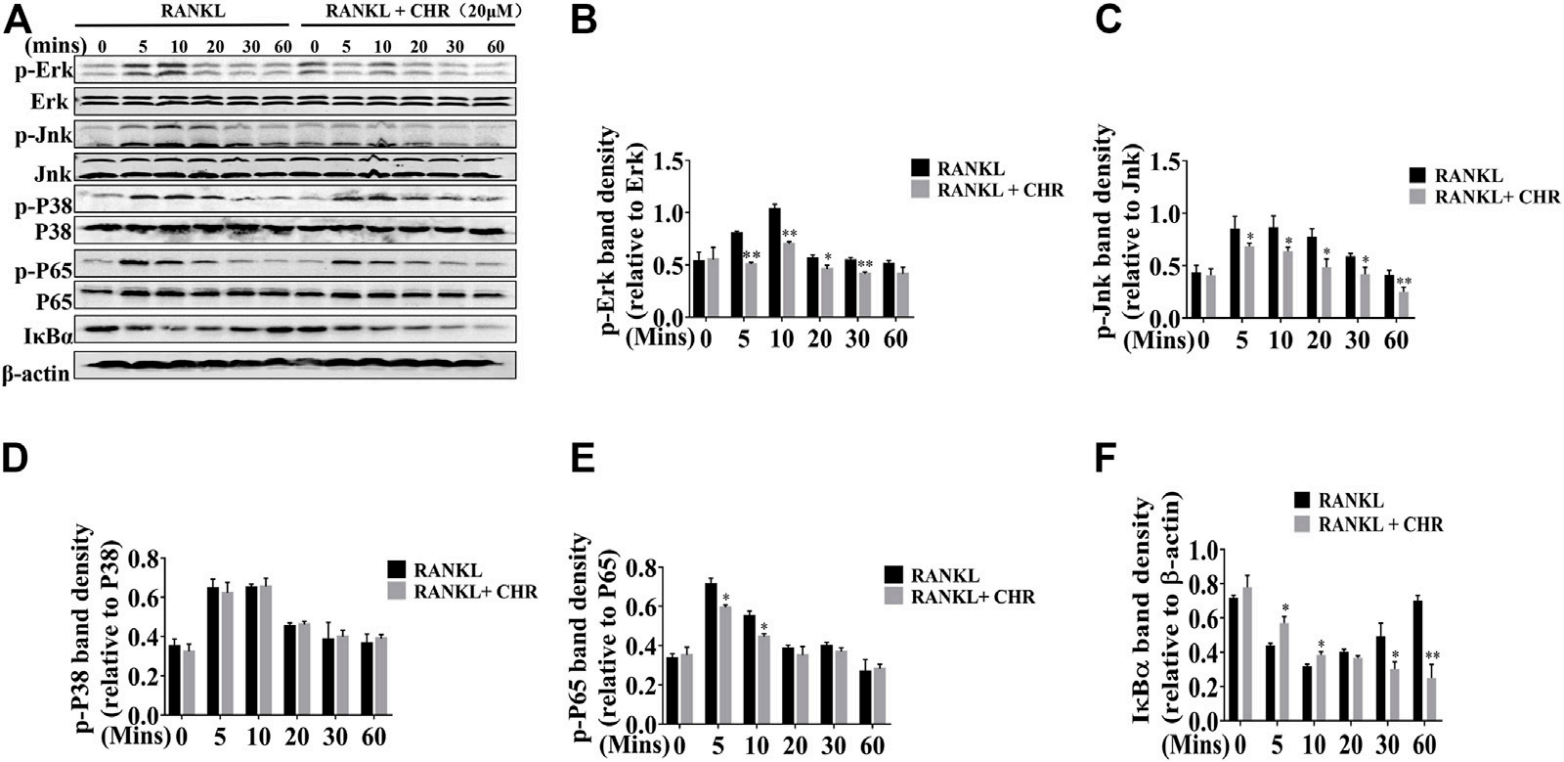
CHR inhibited RANKL-induced MAPKs and NF-κB signaling pathways. **(A)** BMMs cells were pretreated with or without 20 μM CHR for 4 h and stimulated with stimulation with 100 ng/ml RANKL for indicated times. Cells were lysed, total cellular proteins were extracted and subjected to Western blot analysis using specific antibodies against p-ERK, total ERK, p-JNK, total JNK, p-p38, total p38, p-p65, total p65, p-IκBα, and IκBα. Antibody against β-actin was used as internal loading control. The relative density of protein bands for **(B)** p-ERK/total ERK, **(C)** p-JNK/total JNK, **(D)** p-P38/total P38, **(E)** p-p65/total p65, and **(F)** IκBα/β-actin was quantified using ImageJ (n = 3) (*p < 0.05, **p < 0.01, ***p < 0.001).

The NF-κB and MAPK signaling pathways play are critical for RANKL-induced OC formation (Ghosh and Karin, 2002). To determine the molecular mechanism by which CHR inhibits OC formation, we investigated the effects of CHR on the NF-κB and MAPK signaling cascades. Murine BMMs were pretreated with or without 20 μM of CHR and subsequently stimulated with 100 ng/ml RANKL for 5, 10, 20, 30, and 60 min. Total proteins were extracted from cells lysate and then subjected to Western blot. As shown in Figures 4A–D RANKL-induced robust phosphorylation of ERK1/2, JNK1/2, p38 and IκBα, reaching peak level of phosphorylation at around 10 min. On the other hand, in the presence of CHR, phosphorylated pERK1/2 and pJNK1/2, were significantly inhibited, whereas phosphorylated p38 remained unaffected.

**FIGURE 5:**
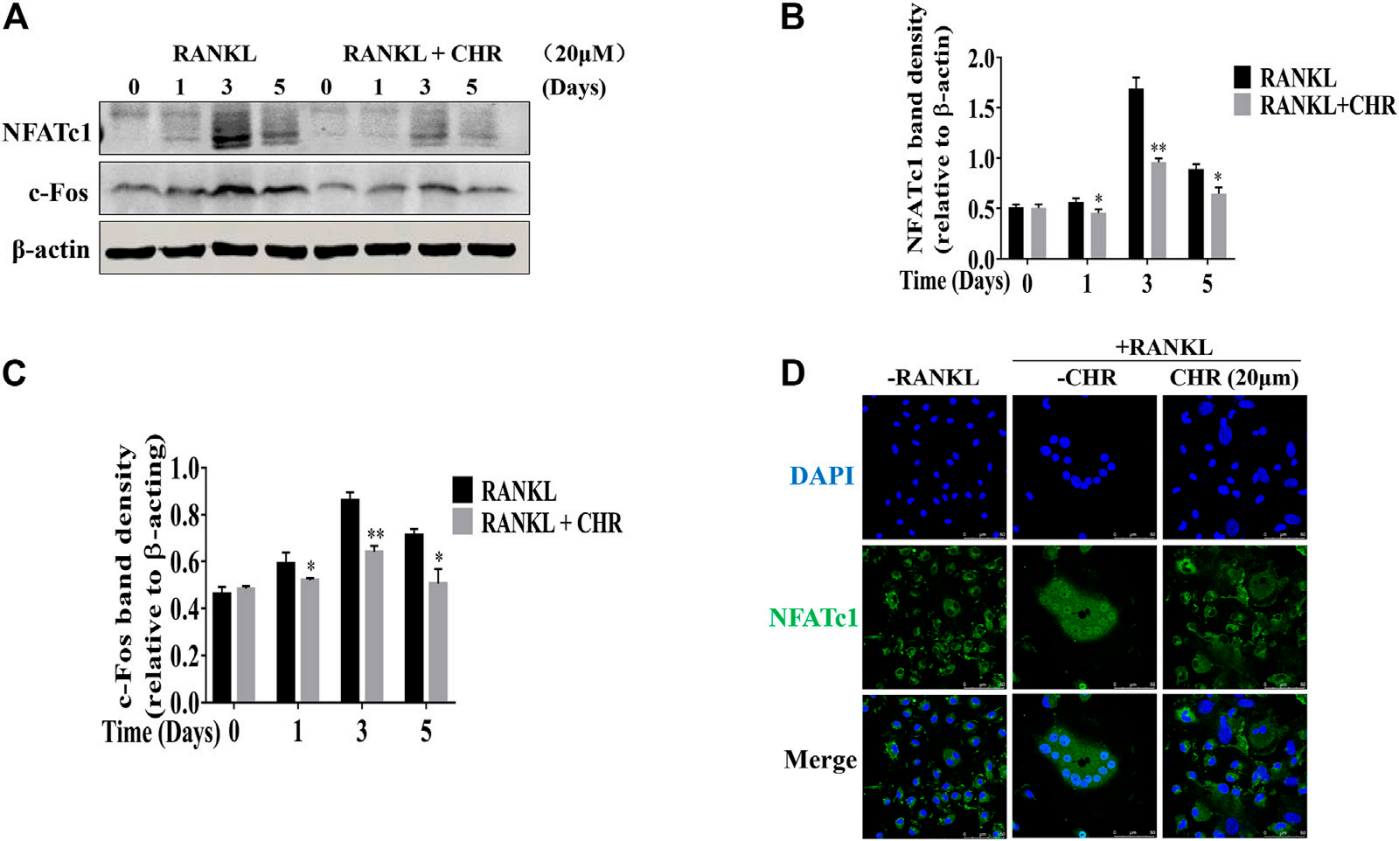
CHR inhibited RANKL induced C-fos and NFATc1 protein expression. **(A)** Murine BMMs were cultured with 30 ng/ml M-CSF and 100 ng/ml RANKL absence or presence of 20 μM CHR for 1, 3, or 5 days and were lysed and total cellular protein extracts were subjected to western blot analysis using specific antibodies to NFATc1 and c-Fos. Antibody to β-actin was used as internal loading control. The relative density of protein bands for **(B)** C-fos and **(C)** NFATc1 against β-actin was quantified using ImageJ. **(D)** Immunofluorescence staining for nuclear localization of NFATc1 (n = 3) (*p < 0.05, **p < 0.01, ***p < 0.001).

CHR similarly exerts inhibitory influence on RANKL-induced NF-κB signaling. Rapid degradation of IκBα is the hallmark for the activation of this signaling cascade. As shown in Figures 4A,E,F, IκBα degraded within 5–10 min with RANKL stimulation, followed by a slow recovery towards basal levels from 20 to 60 min. The degradation of IκBα proteins coincided with the phosphorylation of p65, which also reached peak level among 5 to 10 min after RANKL stimulation. In the presence of CHR, the phosphorylation of p65 was significantly reduced since the delayed degradation of IκBα. Finally, the activation of NF-κB and MAPK culminated the potency as the downstream activation of c-Fos and NFATc1. These two transcriptional factors were indispensable for OC formation and bone resorption (Ikeda et al., 2004; Monje et al., 2005). As can be seen from the results shown in Figures 5A–C, the expression of the RANKL-induced nuclear transcription factors c-Fos and NFATc1 in the nucleus was significantly reduced after treatment with CHR. In addition, as shown in Figure 5D our immunofluorescence assay demonstrated that NFATc1 nuclear translocation was inhibited after treatment with CHR.

Collectively, these results indicate that CHR inhibits both the NF-κB and the MAPK pathways which subsequently down-regulates the expression of key OC transcription factors NFATc1 and c-Fos.

### CHR Inhibited Titanium Particles-Induced Calvarial Osteolysis *In Vivo*

**FIGURE 6:**
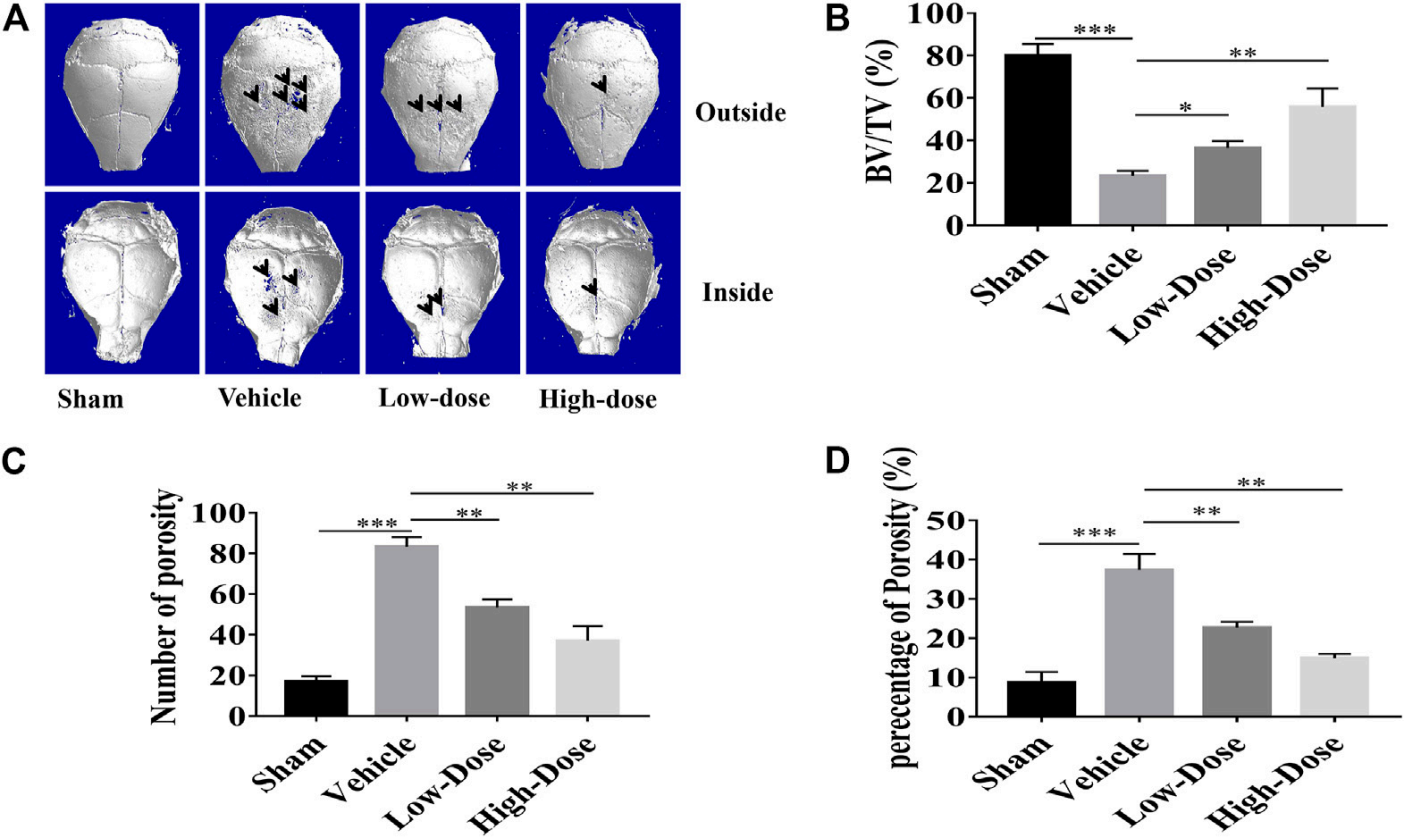
CHR protected against titanium particles-induced osteolysis of mouse calvaria. **(A)** Representative micro-CT three dimensional reconstructed images of calvarial tissue from Sham, PBS vehicle, and CHR treatment groups. Histomorphometric analysis of **(B)** bone volume to tissue volume (BV/TV, %), **(C)** number of bone porosity, and **(D)** the percentage of total bone porosity of treatment groups (*p < 0.05, **p < 0.01, ***p < 0.001).

The titanium particles-induced calvarial osteolysis model was established to mimic *in vivo* function that CHR protected over-activation of osteoclast, as well as its predominant potential as being an anti-osteolytic agent. Thirty milligram titanium particles were implanted under the periosteum in all treating groups except the Sham group (no titanium particles). Mice in CHR-treated group were constantly injected for 2 weeks with 4 mg/kg (low dose) and 8 mg/kg (high dose); respectively every other day. Sham and vehicle groups were injected with the same volume of PBS every other day for 2 weeks. At the end of the experiment, titanium particles-induced calvarial bone destruction was examined using micro-CT scanning. Three dimensional reconstruction of the calvarial bone showed extensive bone resorption and destruction of the bone surface. On the other hand, the degree of surface bone resorption and destruction induced by titanium particles was dose-dependently alleviated in CHR-treated groups (Figure 6A).

**FIGURE 7:**
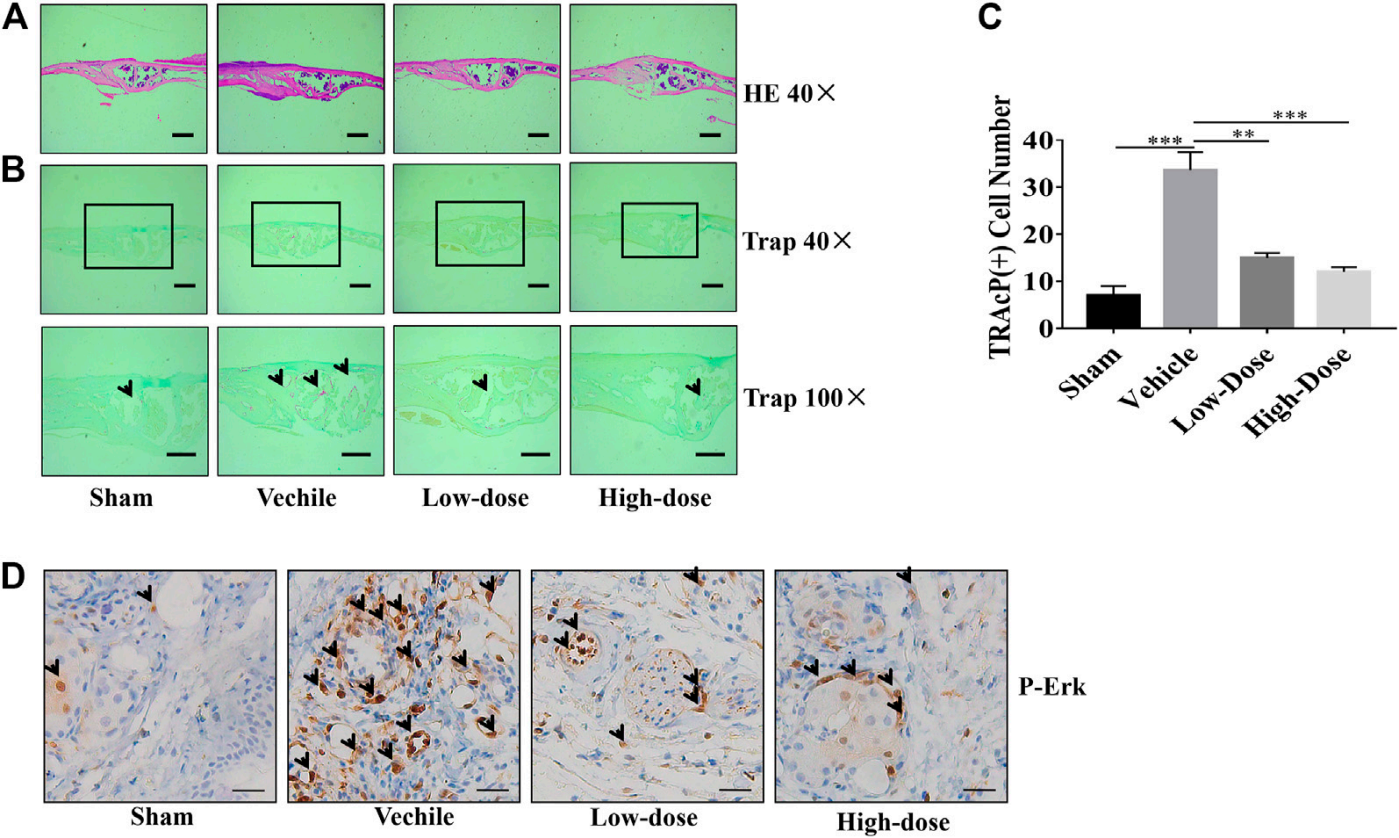
Calvarial tissues from each treatment group were fixed, decalcified, dehydrated, and sectioned for analysis by H&E and TRAcP staining. Representative images of calvaria stained with H&E **(A)** and TRAcP **(B)**, histological assessment of **(C)**, and numbers of TRAcP positive OC were shown. **(D)** Immunohistochemical staining of phosphorylated ERK in calvaria sections (scale bar = 500 µM) (**p < 0.01, ***p < 0.001).

Three major indicators of bone histomorphometric parameters, bone volume/tissue volume (BV/TV), the amount of bone porosity, and the percentage of total bone porosity, were applied for evaluation of bone integrity (Figures 6B–D). BV/TV of the calvarial bone was dose-dependently increased following treatment with CHR as compared to the PBS-treated vehicle group (Figure 6B). Consistent with this effect, porosity number and the percentage of total porosity were also dose-dependently reduced towards Sham levels in CHR treated groups as compared with PBS-treated vehicle group (Figures 6C,D). Histological images provided evidence that CHR protected calvarial osteolysis from titanium particles induction (Figures 7A–C). H&E and TRAcP staining showed serious bone destruction in the vehicle group with numerous TRAcP positive multinucleated OCs and larger amounts of active OCs (Figures 7A–C) which accord to the micro-CT and histomorphometric results. Meanwhile, immunohistochemical staining showed significant activation of phosphorylated ERK in the vehicle group (Figure 7D), and the phosphorylated ERK in the treatment group showed a downward trend, which was consistent with *in vitro* Western blot assay. Taken together, our *in vivo* data suggests that CHR has a protective effect against titanium particles-induced calvarial osteolysis via attenuation of OC formation and function as demonstrated in our *in vitro* experiments.

## Discussion

Over-activation of OCs in response to implant-derived wear particles remains the key cause of osteolysis around the prosthetic joints (Jiang et al., 2013; Langdahl et al., 2016). Clinically, drugs targeting OC formation and function have shown varying levels of effectiveness against osteolysis and osteoporosis. For example, although bisphosphonates have been clinically used for osteolytic conditions, their use is associated with a number of negative side effects including gastrointestinal complications, musculoskeletal pain, and osteonecrosis of the jaw (Verron and Bouler, 2014). Thus, there are still unmet needs for the discovery of novel agents that can specifically target the OC for the effective treatment of local osteolysis.

Traditional Chinese medicine monomers which extracted from natural plants such as Artesunate and Luteoloside have demonstrated inhibitory effect on OC formation and its biological function (Song et al., 2018; Wei et al., 2018). In the present study, for the first time we showed the protection ability of CHR in bone resorption of CHR *in vivo* and *in vitro*. This bioflavonoid was discovered with a good suppression of osteoclast formation, as well as delaying the bone resorption function of osteoclasts*.* Besides, *in vivo* verification illustrated bioactivity of CHR in preventing titanium particles-induced calvarial osteolysis after administration. Moreover, CHR demonstrated no cytotoxicity at a range of concentration (5–20 μM) that inhibited OC formation and bone resorption through RANKL-induced pathways. Furthermore, our data also suggested that CHR exerted its inhibitory effect at early stage of OC formation. Consistent with the attenuation of RANKL-induced OC formation, the expression of OC marker genes such as *Ctr, Mmp9, Acp5, Atp6v0d2, Ctsk*, and *Dc-stamp* were down-regulated after treating with CHR. In addition, we found that CHR could inhibit F-actin belts formation in mature OCs.

The inhibitory effect of CHR on OC formation and function of bone resorption contributed to the protective effect of CHR on titanium particles-induced calvarial bone destruction mediated by OCs *in vivo*. Treatment with CHR every 2 days for 14 days effectively suppressed TRAcP positive OC formation and bone resorption induced by titanium particles. Micro-CT imaging and related histomorphometric analyses demonstrated the damage extent of calvarial bone was relieved in CHR treated groups. Histological assessment by H&E and TRAcP staining further verified that the reduction in bone loss was contributed to the drop of count of TRAcP positive OCs in CHR treated groups.

NF-κB activation is one of the most significant early signaling events induced by RANKL, and perturbations in this signaling axis prevents osteoclastogenesis *in vitro* and leads to osteopetrosis *in vivo* (Soysa and Alles, 2009). On the other hand, over-activation of NF-κB is associated with osteoclastic bone diseases (Xu et al., 2009). IκB kinase (IKK) complex breaks the inactive statue of inhibitor-κBs (IκBs) by phosphorylation which essentially triggers the NF-κB signaling activation. The rapid phosphorylation of IκBs and its subsequent proteosome degradation permits the downstream phosphorylation, nuclear translocation, and activation of NF-κB subunit p65/RelA (Iotsova et al., 1997). In our study, CHR markedly delayed IκBα phosphorylation and degradation, and in turn suppressed nuclear translocation of p65/RelA.

Mitogen-activated protein kinase (MAPK) pathways include extracellular signal-regulated kinase (ERK), c-Jun n-terminal kinase (JNK), and p38, which are all rapidly phosphorylated in response to RANKL and also plays crucial roles in the early stages of OC formation, activation, and survival (Hotokezaka et al., 2002; Stevenson et al., 2011). In this study, we found that CHR could suppress the phosphorylation and activation of ERK and JNK, but not the activation of p38. CHR has also been shown to inhibit phosphorylation of ERK *in vivo*. The inhibition of ERK and JNK suggests that CHR may target a common upstream regulator (such as a common MEK) within the JNK/ERK axis, of which further investigations are required.

Following activation, the NF-κB and MAPKs synergistically activate the transcriptional activities of key transcription factors in the nucleus c-Fos and NFATc1 (Miyamoto, 2011). In particular, NF-κB is required for the initial induction of NFATc1 and the MAPK/c-Fos signaling axis plays an important role in further induction and activity of NFATc1. Being regarded as the prioritized and the most distal transcription factor, NFATc1 regulated OC-specific marker genes including *Ctr, Mmp9, Acp5, Atp6v0d2, Ctsk*, and *Dc-sStamp* (Kim et al., 2008). Consistent with attenuated activation of ERK, JNK, and NF-κB, the induction of c-Fos and NFATc1 proteins was significantly decreased in the presence of CHR. The reduced induction of NFATc1 protein expression suggests decrease transcriptional activity which was consistent with the observed decreased gene expression of *Ctr, Mmp9, Acp5, Atp6v0d2, Ctsk*, and *Dc-stamp* (Phan et al., 2004; Feng et al., 2009) in the presence of CHR. Our signaling experiments strongly suggest that the anti-osteoclastogenic effect of CHR can be attributed to inhibition of early NF-κB and MAPK signaling pathway which in turns down-regulated the expression and activity of the master transcription regulator NFATc1 for OC formation.

Collectively, the results in our study demonstrated that CHR has a dramatic inhibition on osteoclastogenesis and function of bone resorption *in vitro* through the suppression of crucial RANKL-mediated signaling pathways. CHR exerted protective effects on titanium particles-induced osteolysis in the murine calvarial model *in vivo* by directly acting on OCs to attenuate OC-mediated bone destruction. These data presented in this study suggest that CHR is a potential therapeutic candidate for the attenuation of osteolytic diseases.

## Data Availability

The original contributions presented in the study are included in the article/Supplementary Material, further inquiries can be directed to the corresponding authors.
