# Supplementary figures and images for: Chrysin Protects Against Titanium Particle-Induced Osteolysis by Attenuating Osteoclast Formation and Function by Inhibiting NF-κB and MAPK Signaling
Source: Front Pharmacol. 2022 Mar 23;13:793087. doi: 10.3389/fphar.2022.793087 (PMC8985127; doi:10.3389/fphar.2022.793087)

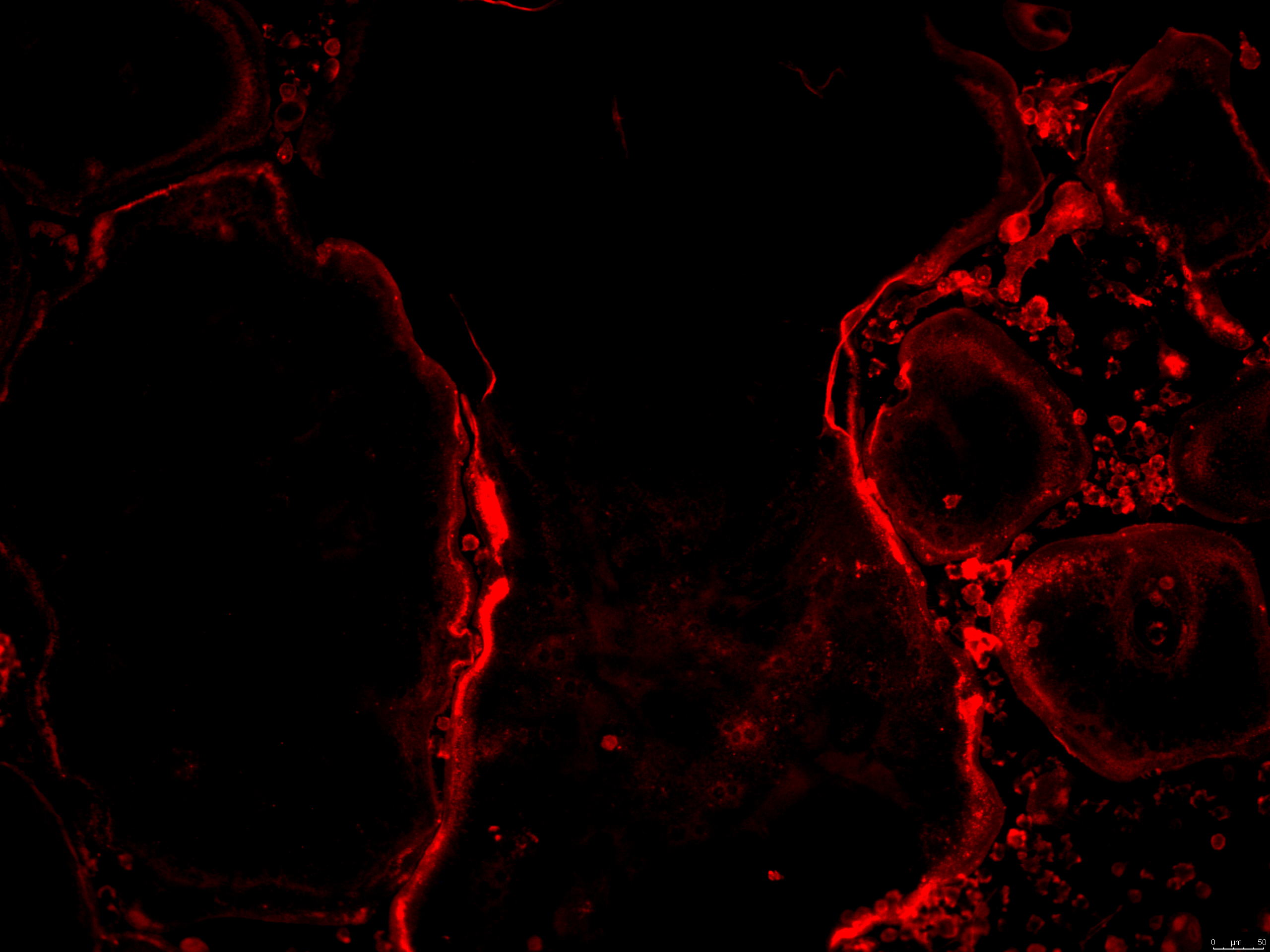

Supplement: Supplementary file 1 [file DataSheet3.ZIP › Figure3/OSTEOCLAST- FUNCTION/0-actin.tif]

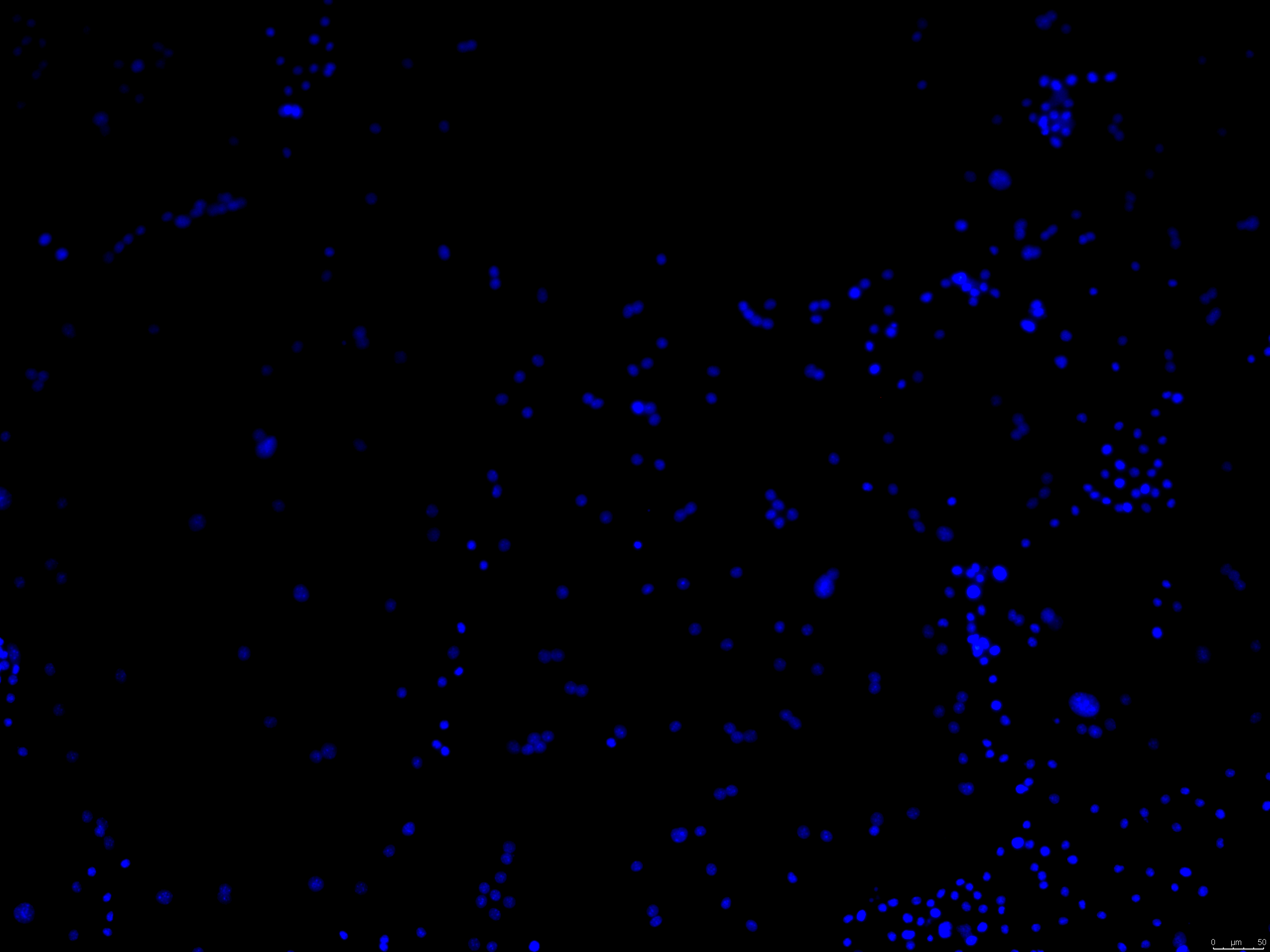

Supplement: Supplementary file 1 [file DataSheet3.ZIP › Figure3/OSTEOCLAST- FUNCTION/0-dapi.tif]

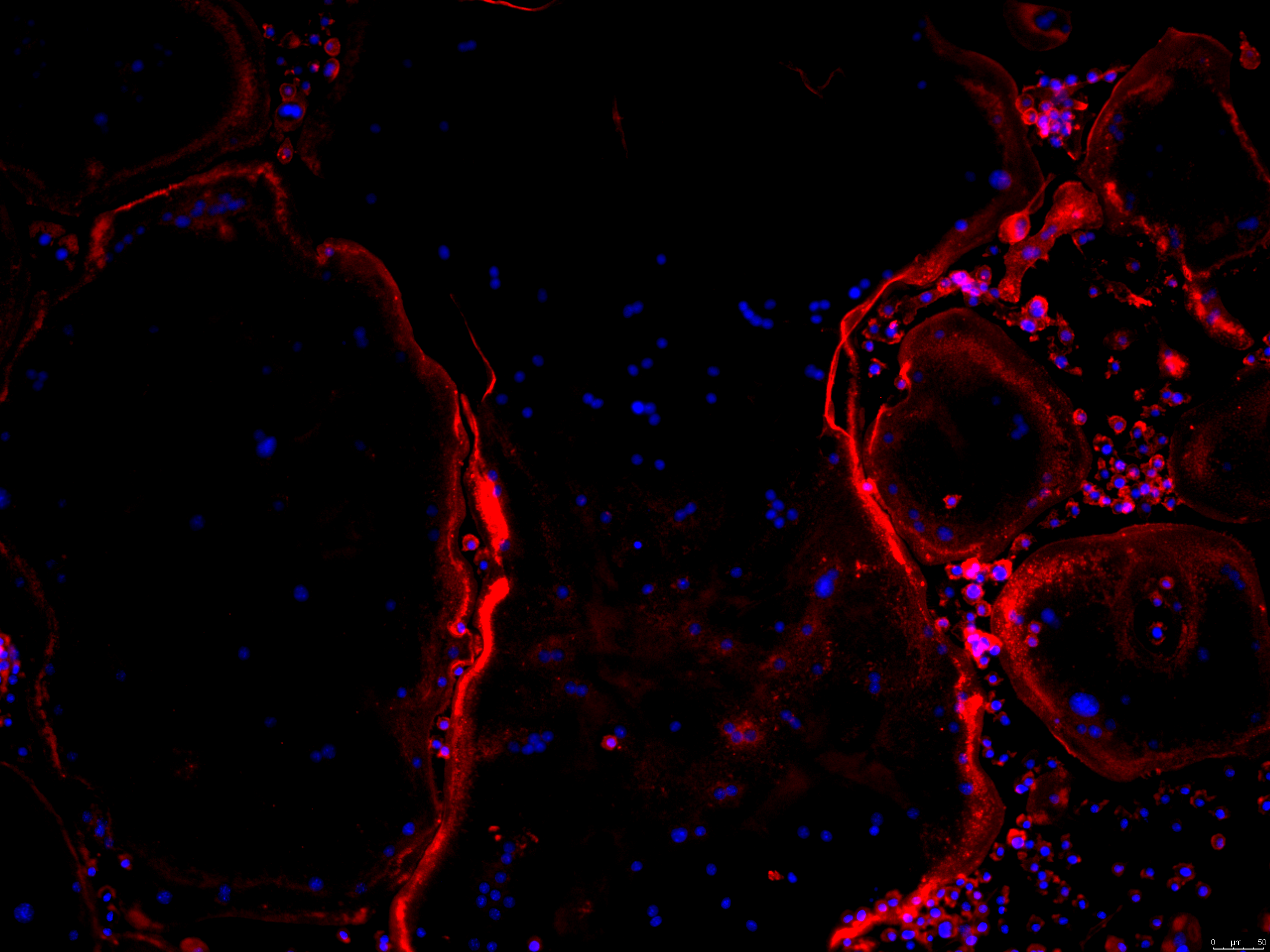

Supplement: Supplementary file 1 [file DataSheet3.ZIP › Figure3/OSTEOCLAST- FUNCTION/0-merge .tif]

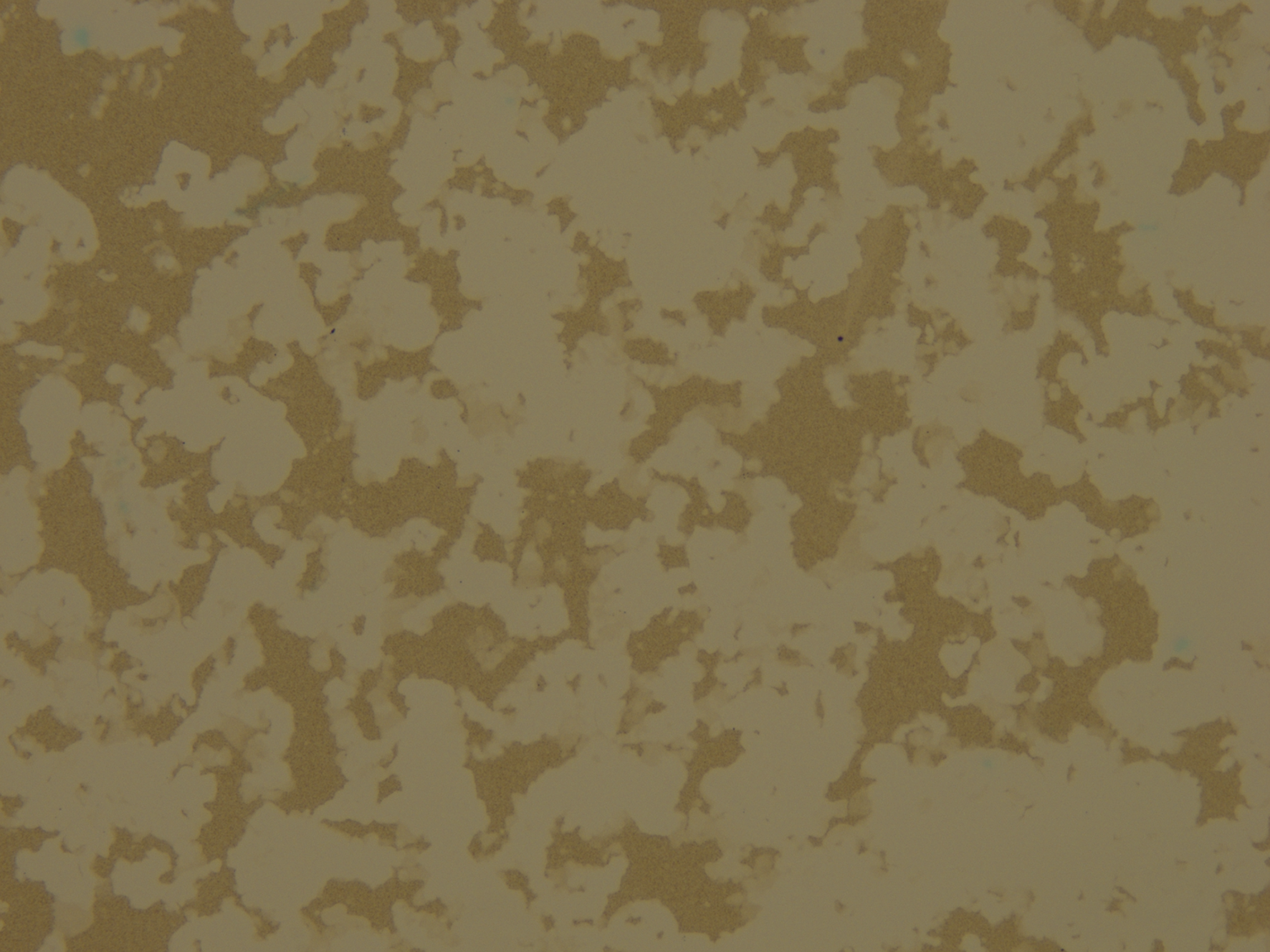

Supplement: Supplementary file 1 [file DataSheet3.ZIP › Figure3/OSTEOCLAST- FUNCTION/0.tif]

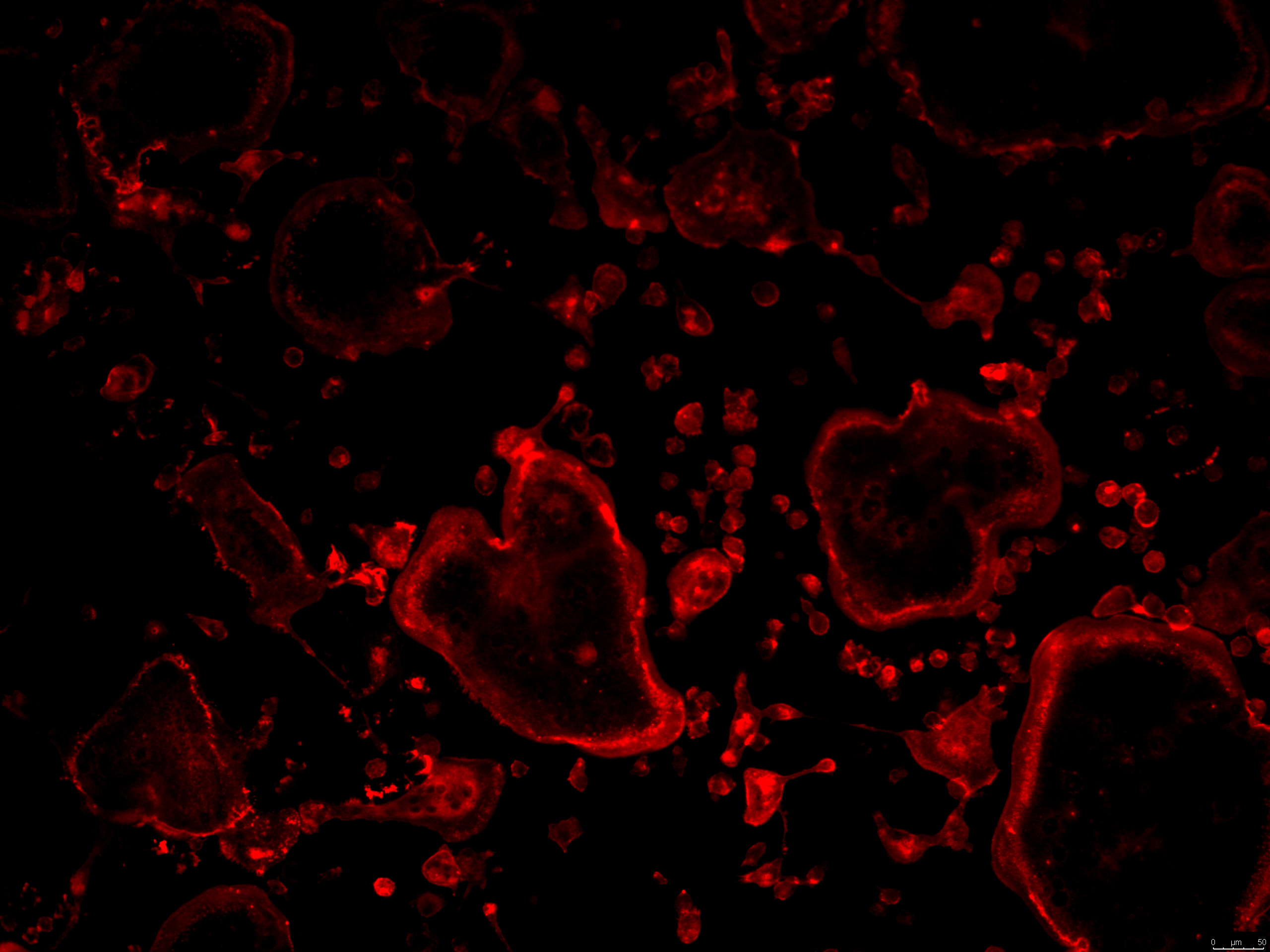

Supplement: Supplementary file 1 [file DataSheet3.ZIP › Figure3/OSTEOCLAST- FUNCTION/10um-actin.tif]

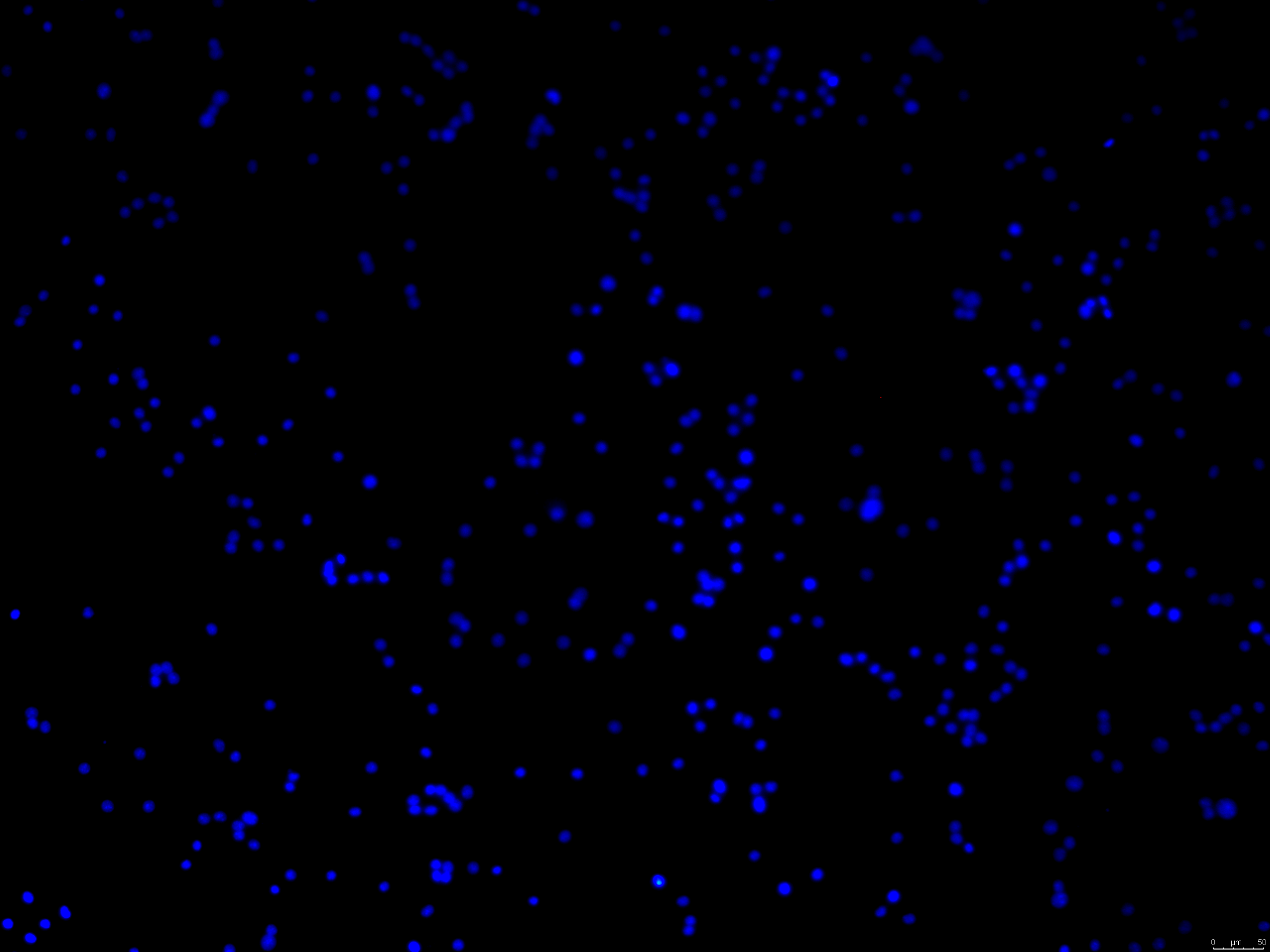

Supplement: Supplementary file 1 [file DataSheet3.ZIP › Figure3/OSTEOCLAST- FUNCTION/10um-dapi.tif]

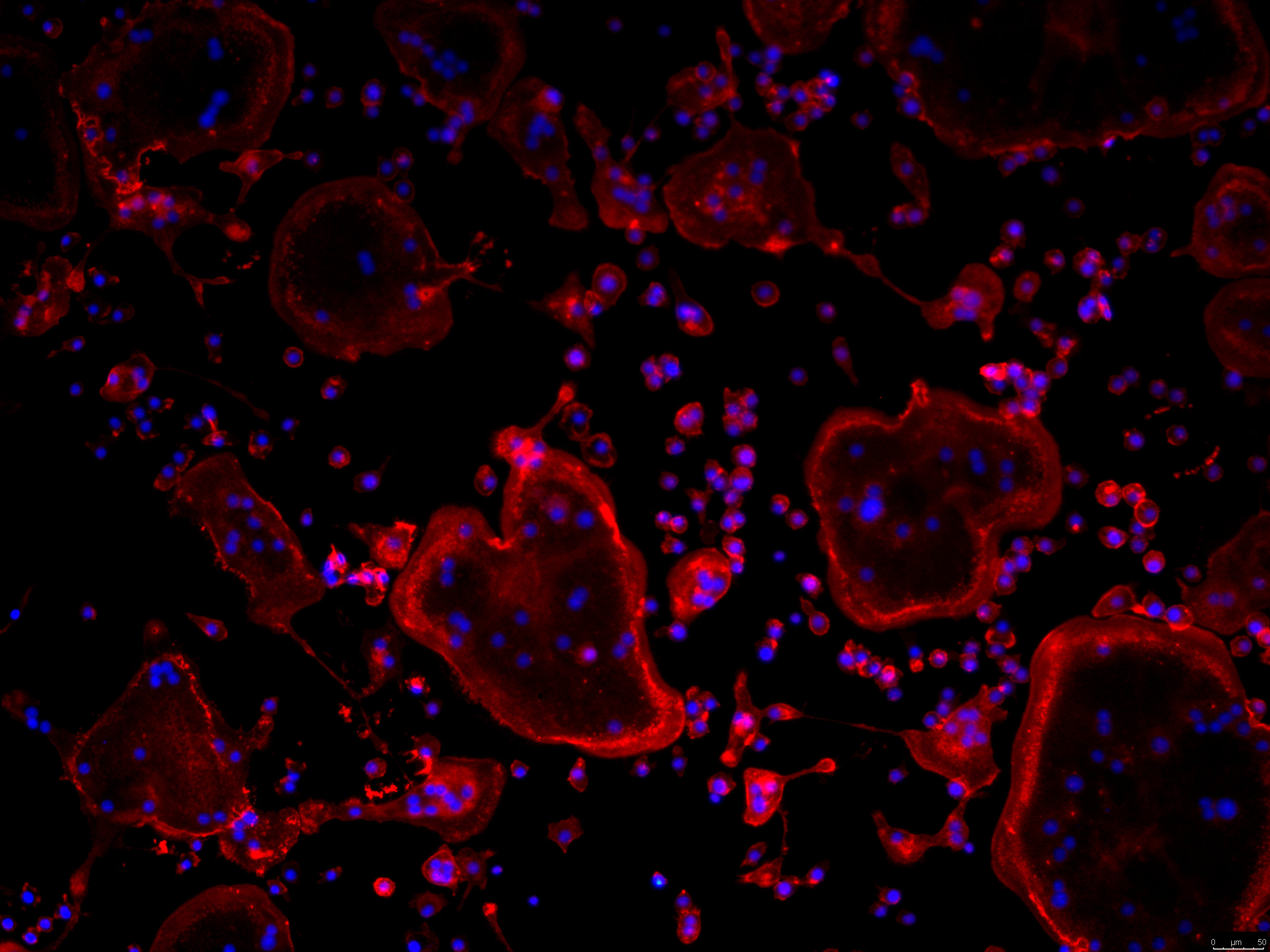

Supplement: Supplementary file 1 [file DataSheet3.ZIP › Figure3/OSTEOCLAST- FUNCTION/10um-merge.tif]

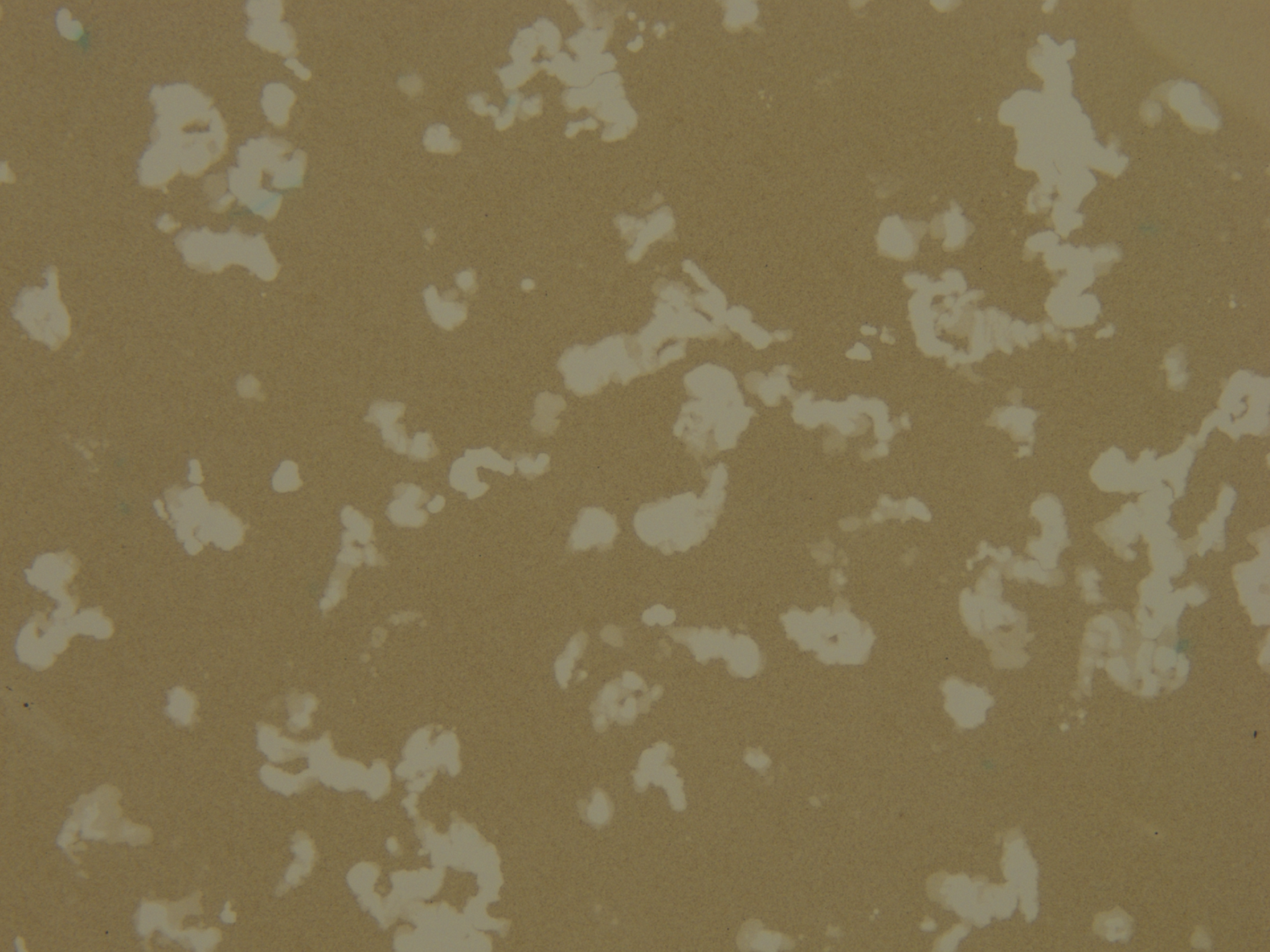

Supplement: Supplementary file 1 [file DataSheet3.ZIP › Figure3/OSTEOCLAST- FUNCTION/10um.tif]

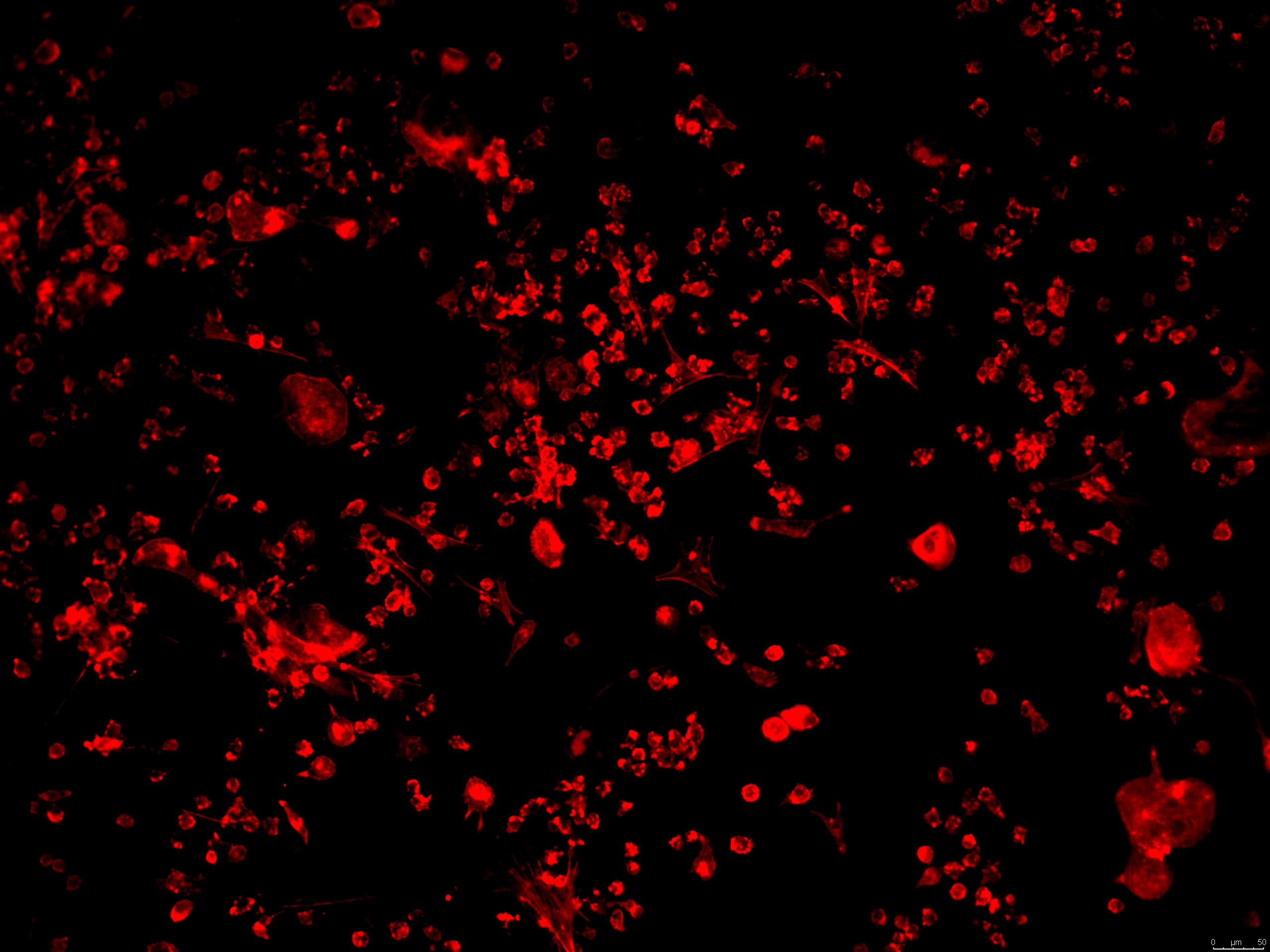

Supplement: Supplementary file 1 [file DataSheet3.ZIP › Figure3/OSTEOCLAST- FUNCTION/20um-actin .tif]

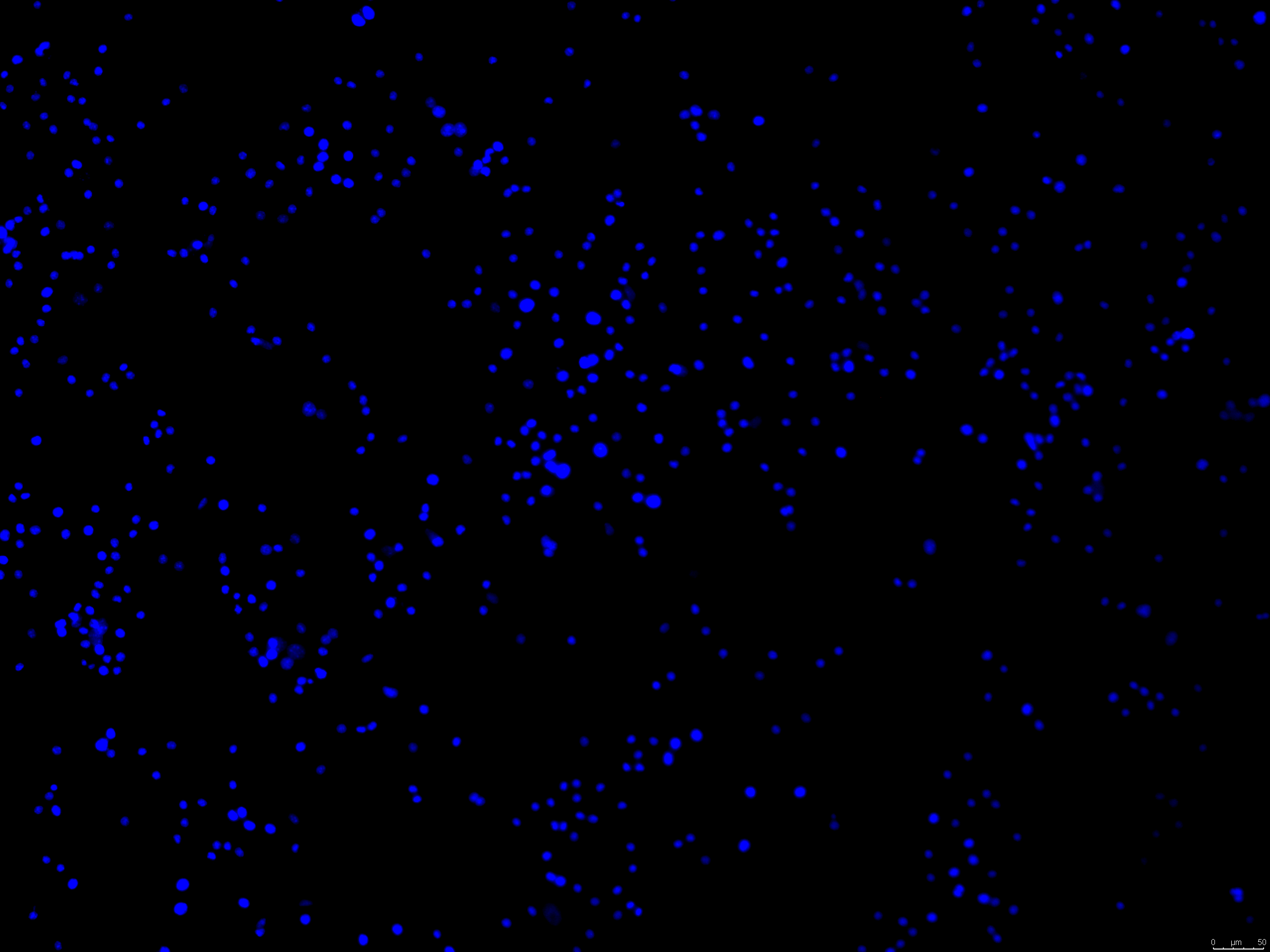

Supplement: Supplementary file 1 [file DataSheet3.ZIP › Figure3/OSTEOCLAST- FUNCTION/20um-dapi.tif]

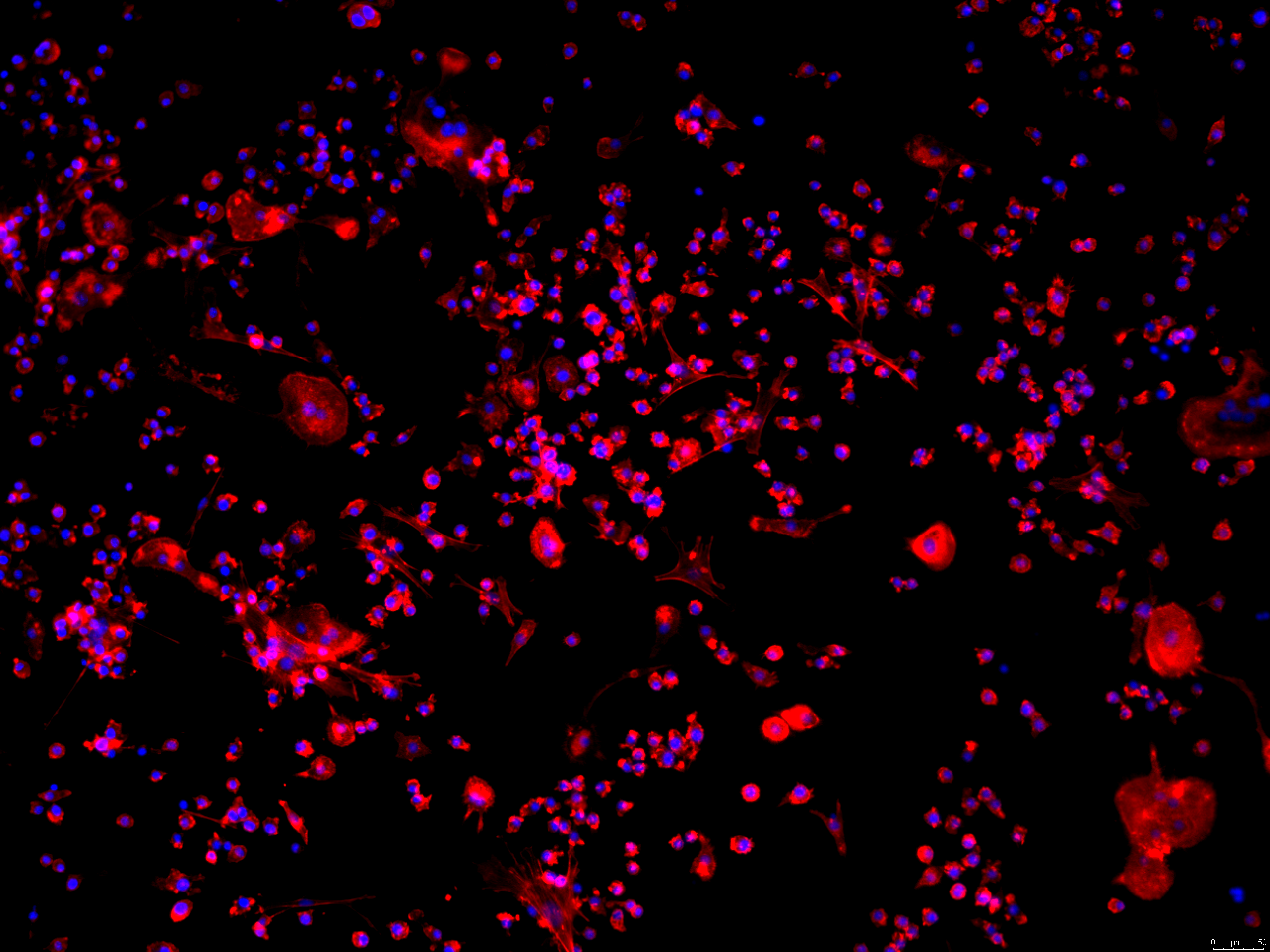

Supplement: Supplementary file 1 [file DataSheet3.ZIP › Figure3/OSTEOCLAST- FUNCTION/20um-merge .tif]

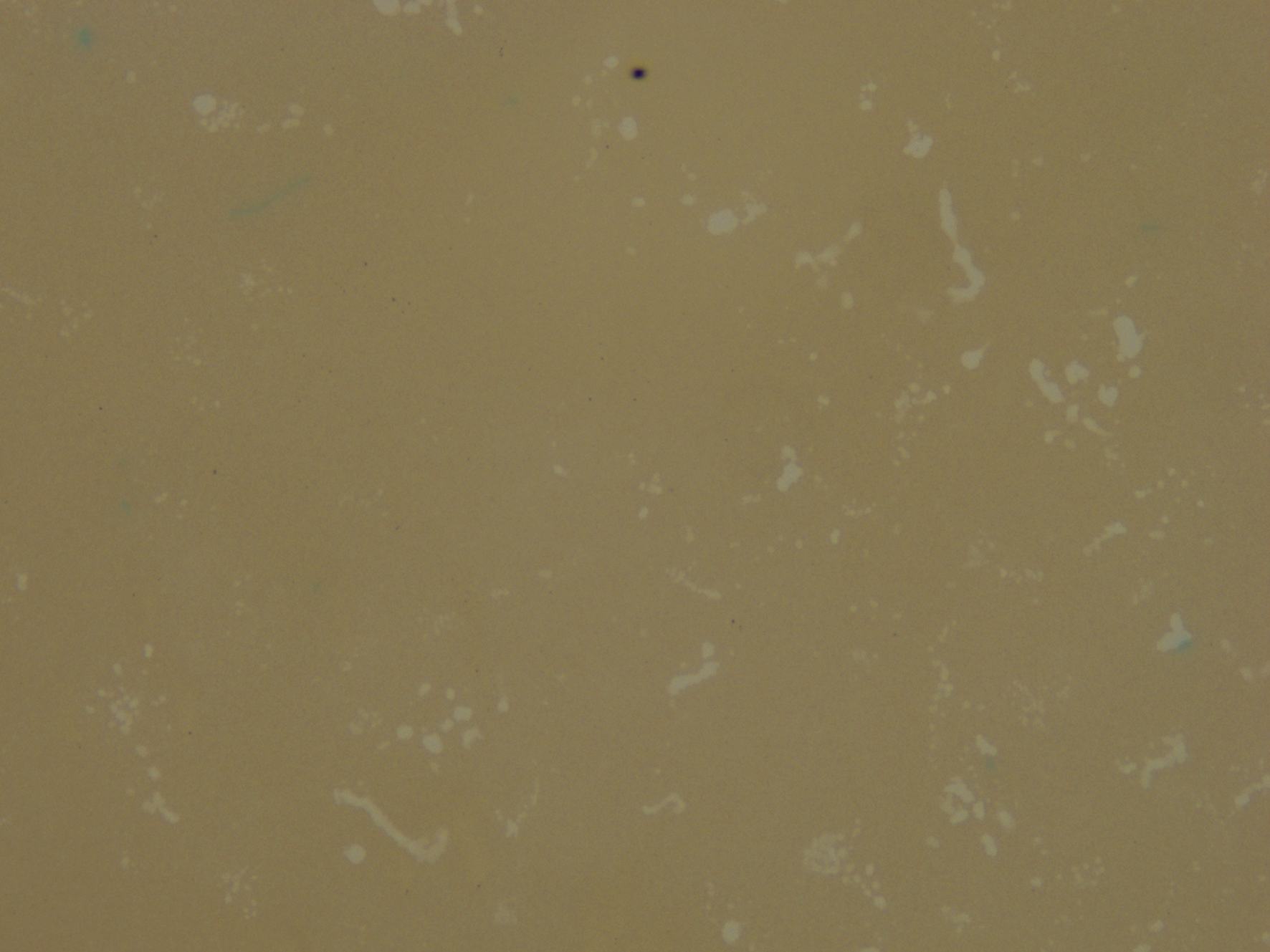

Supplement: Supplementary file 1 [file DataSheet3.ZIP › Figure3/OSTEOCLAST- FUNCTION/20um.tif]

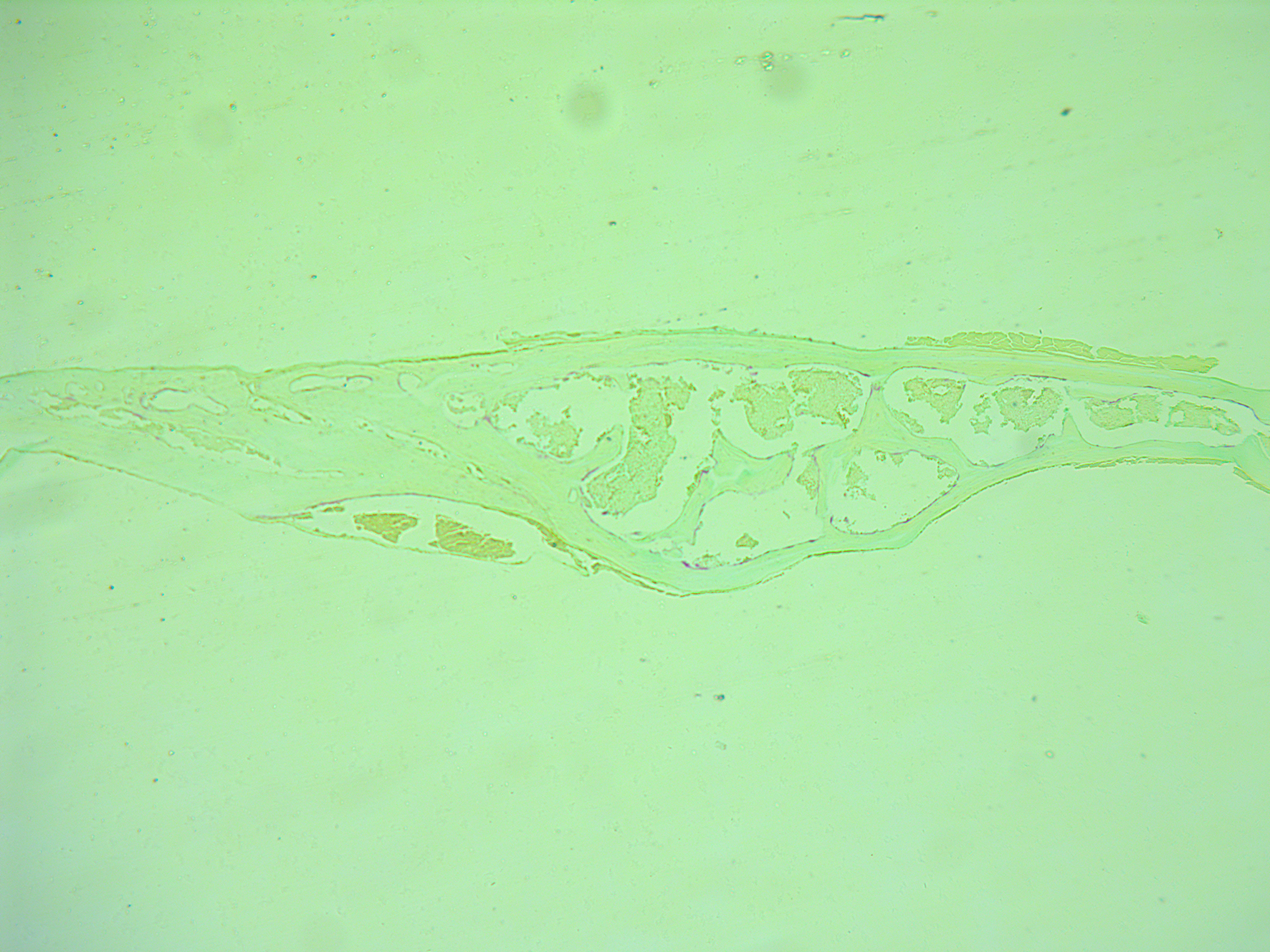

Supplement: Supplementary file 2 [file DataSheet8.ZIP › figure 7-40x-trap/LOW-dose 40X trap.tif]

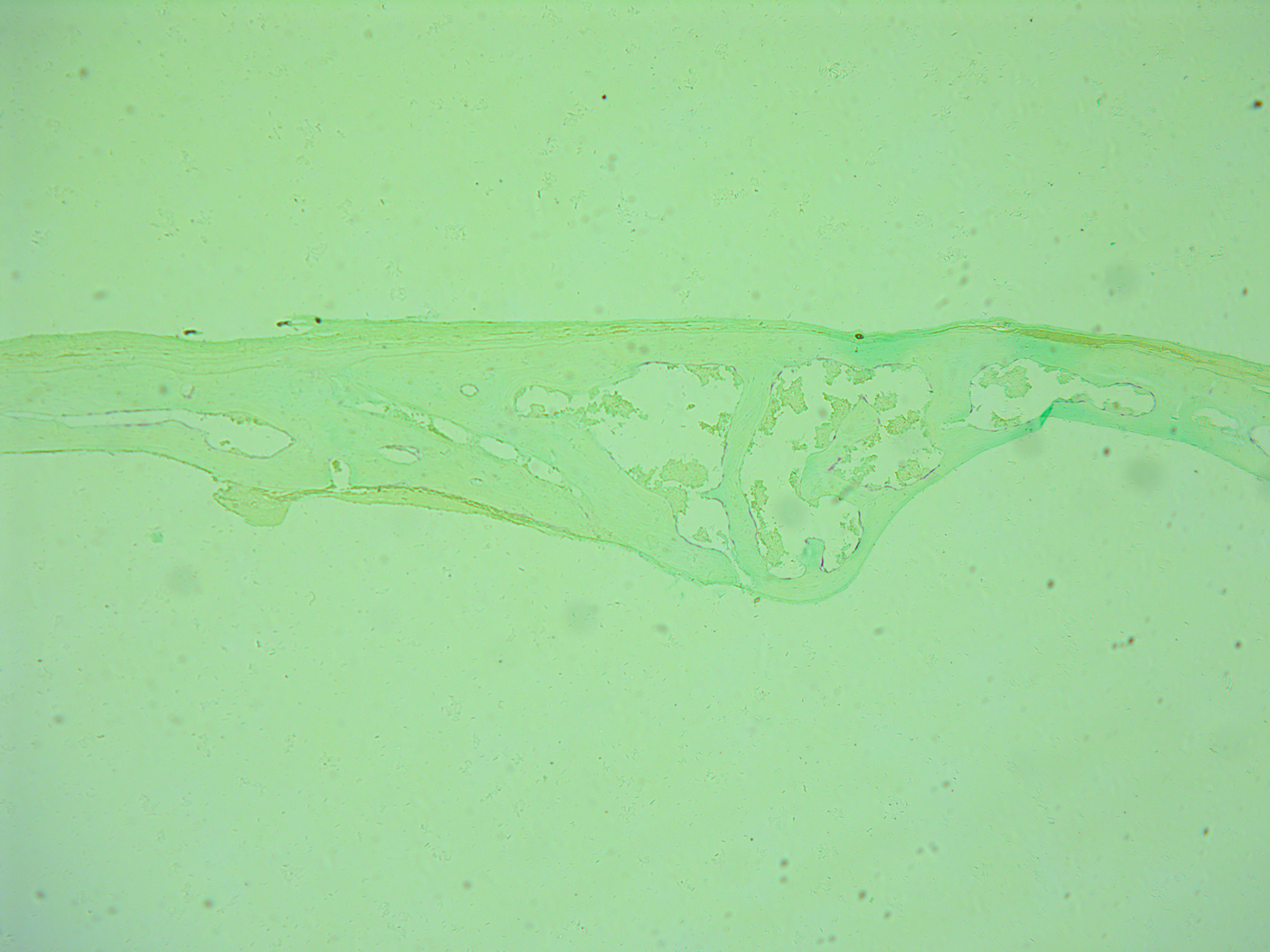

Supplement: Supplementary file 2 [file DataSheet8.ZIP › figure 7-40x-trap/high-dose 40X trap.tif]

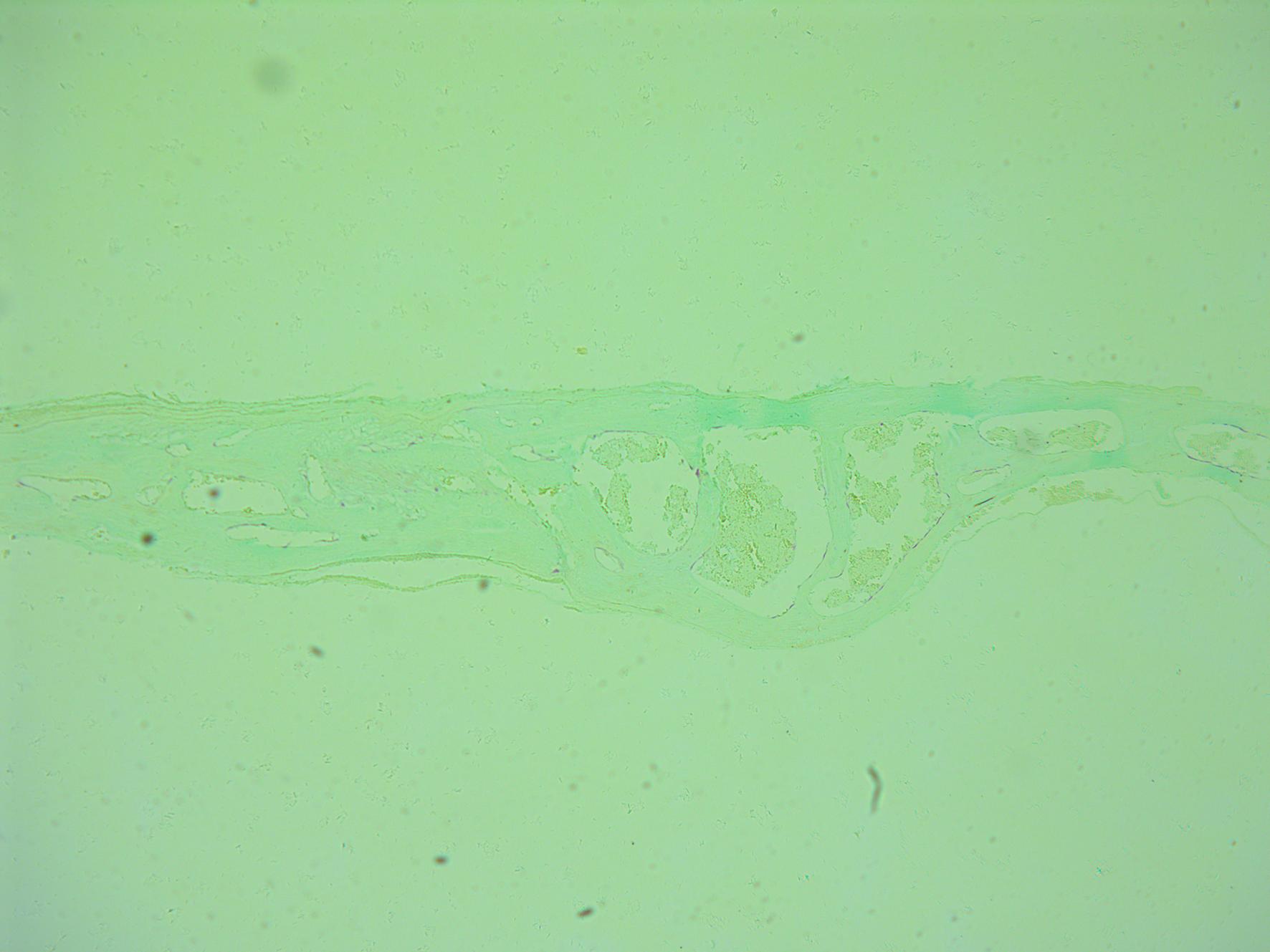

Supplement: Supplementary file 2 [file DataSheet8.ZIP › figure 7-40x-trap/sham 40X TRAP.tif]

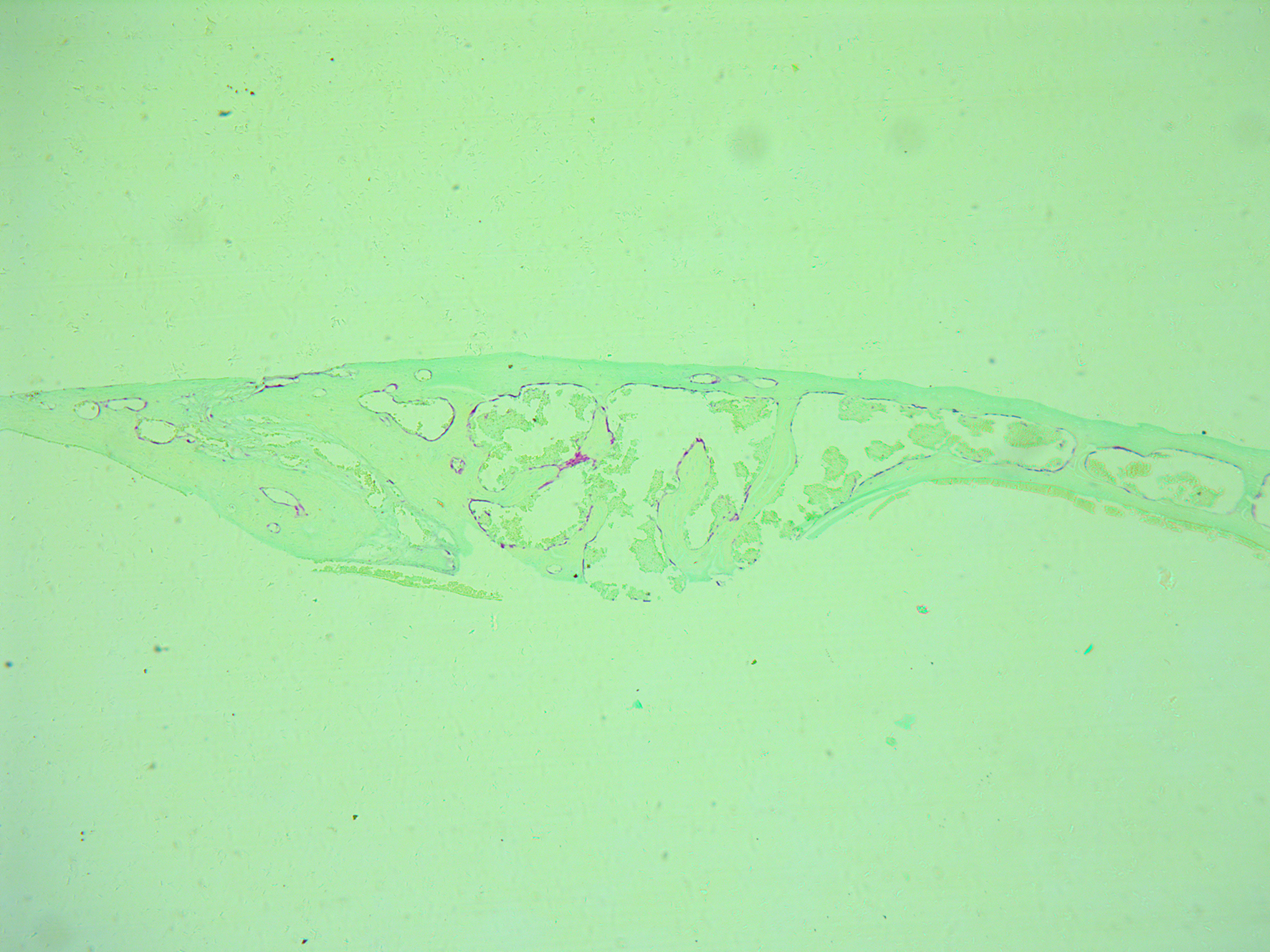

Supplement: Supplementary file 2 [file DataSheet8.ZIP › figure 7-40x-trap/vehicle 40X trap.tif]

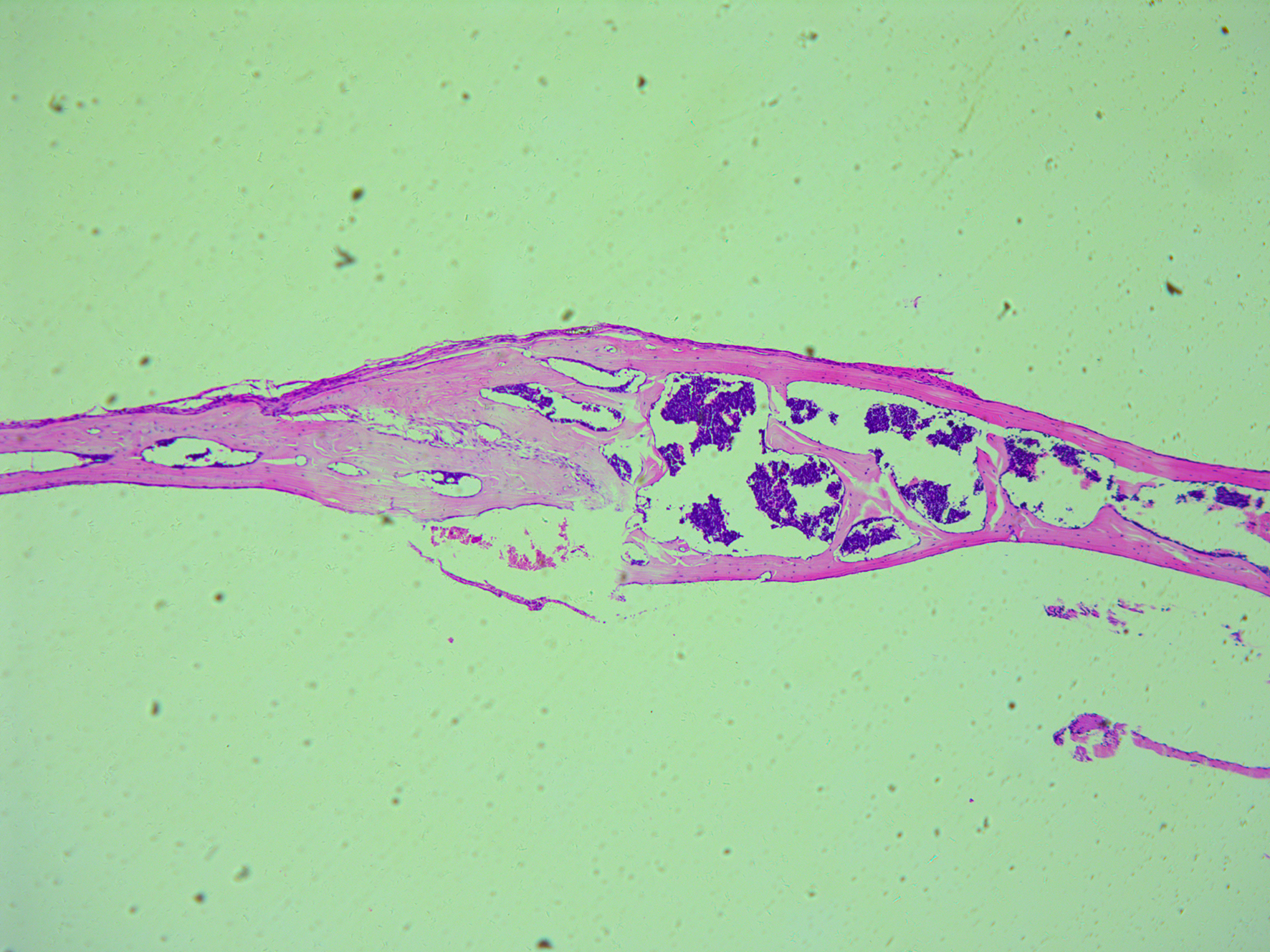

Supplement: Supplementary file 3 [file DataSheet9.ZIP › figure 7-40x-he/high 40X HE.tif]

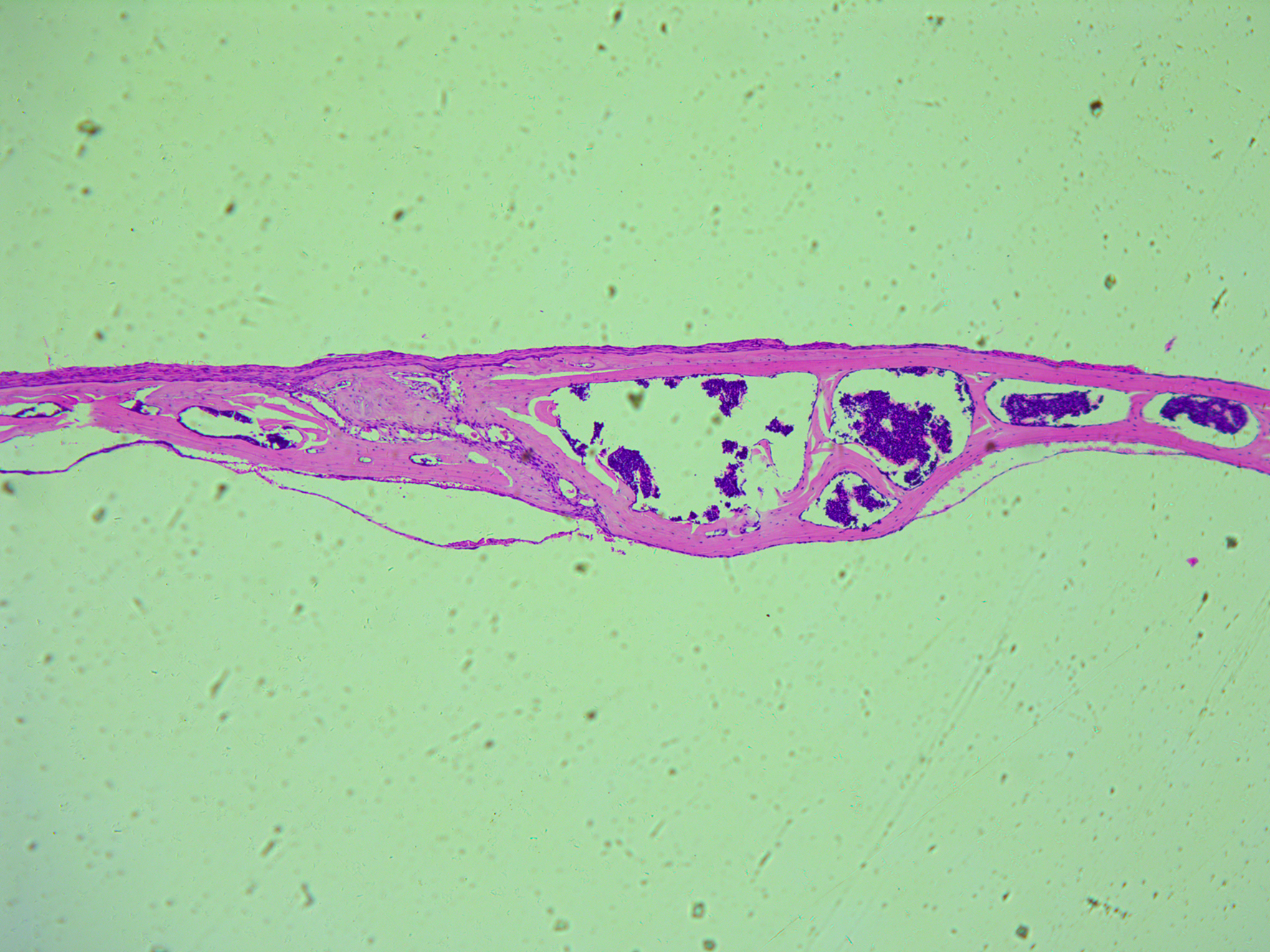

Supplement: Supplementary file 3 [file DataSheet9.ZIP › figure 7-40x-he/low 40X he.tif]

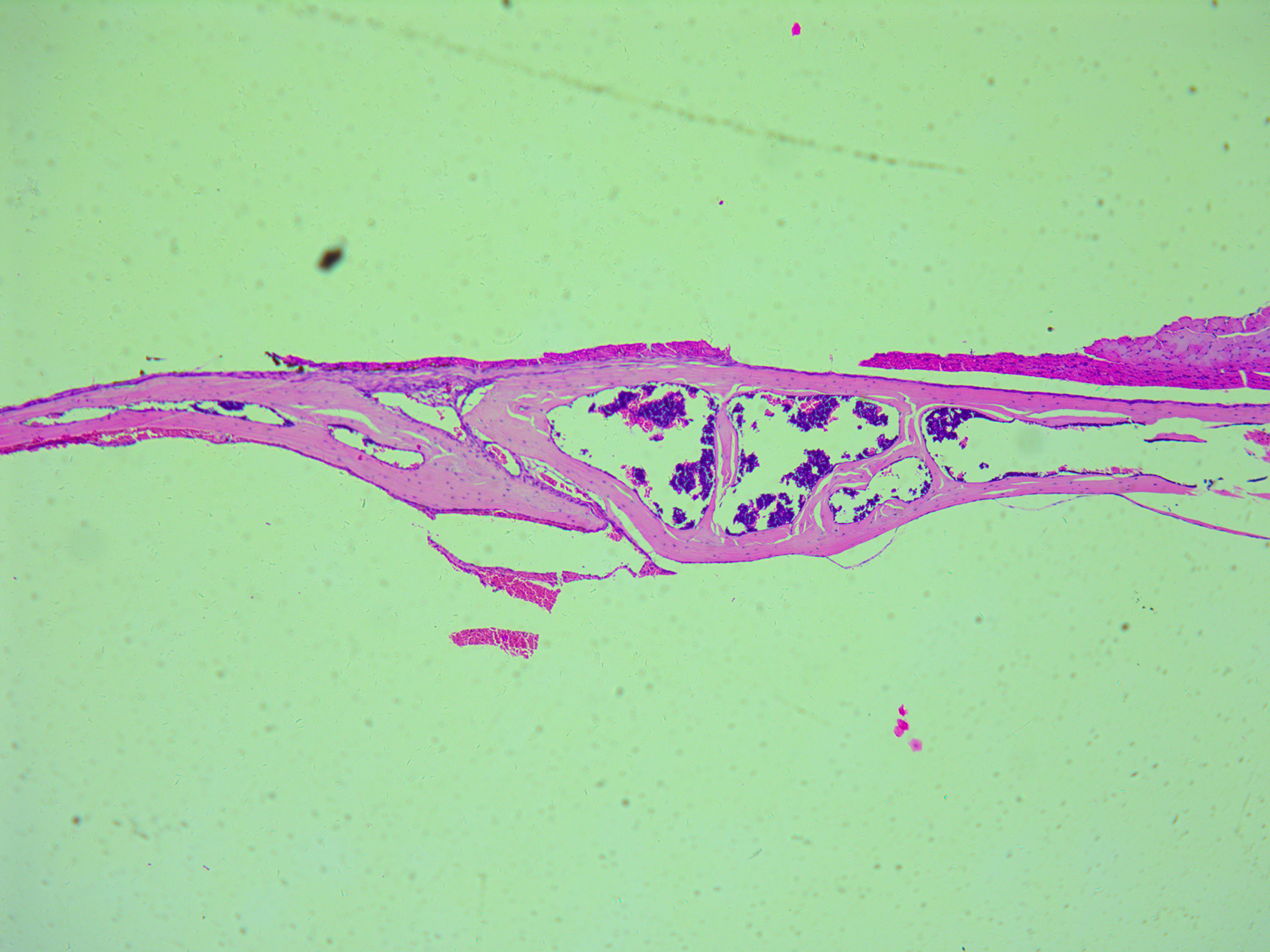

Supplement: Supplementary file 3 [file DataSheet9.ZIP › figure 7-40x-he/sham 40X HE.tif]

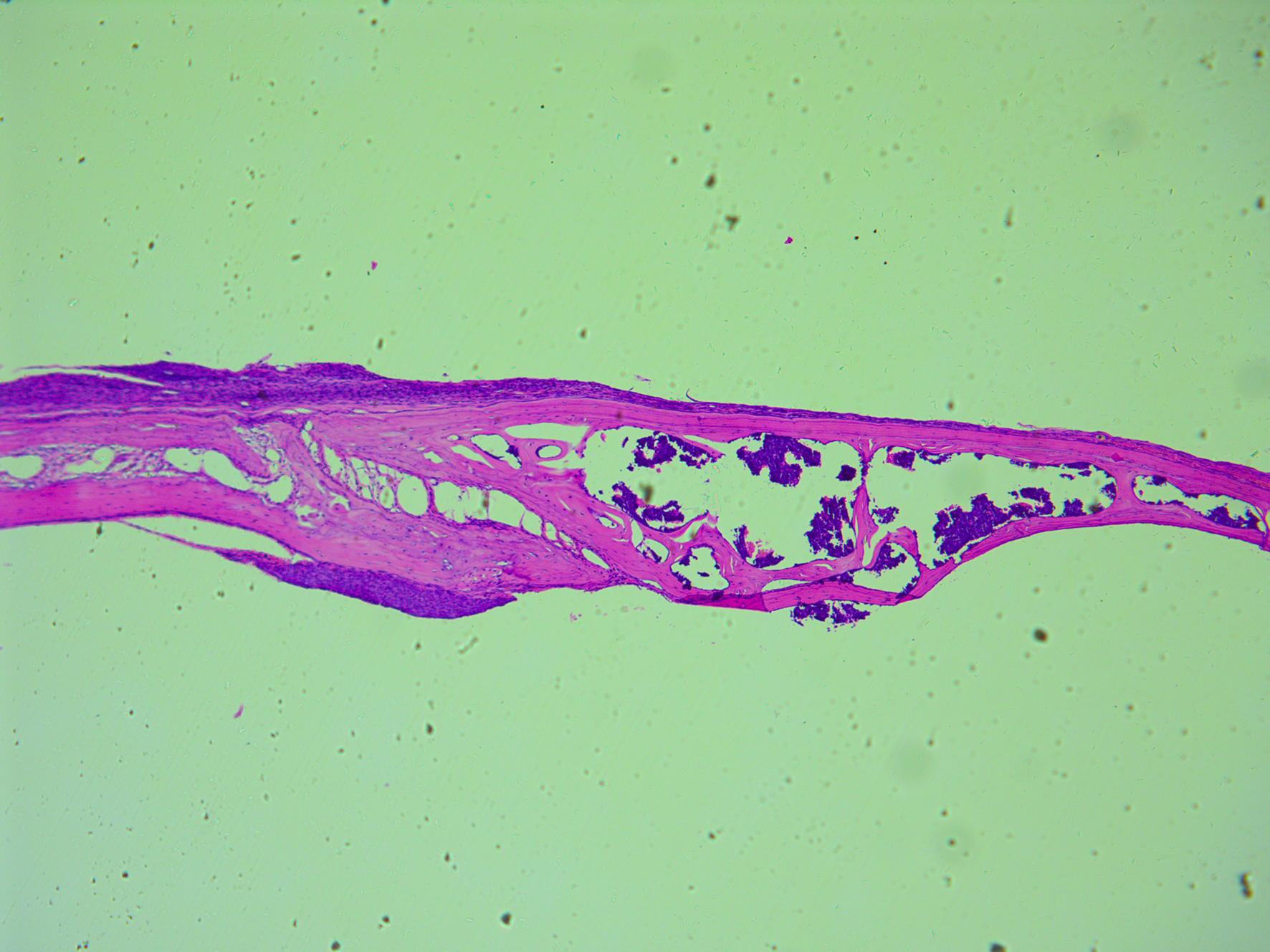

Supplement: Supplementary file 3 [file DataSheet9.ZIP › figure 7-40x-he/vehicle 40X he.tif]

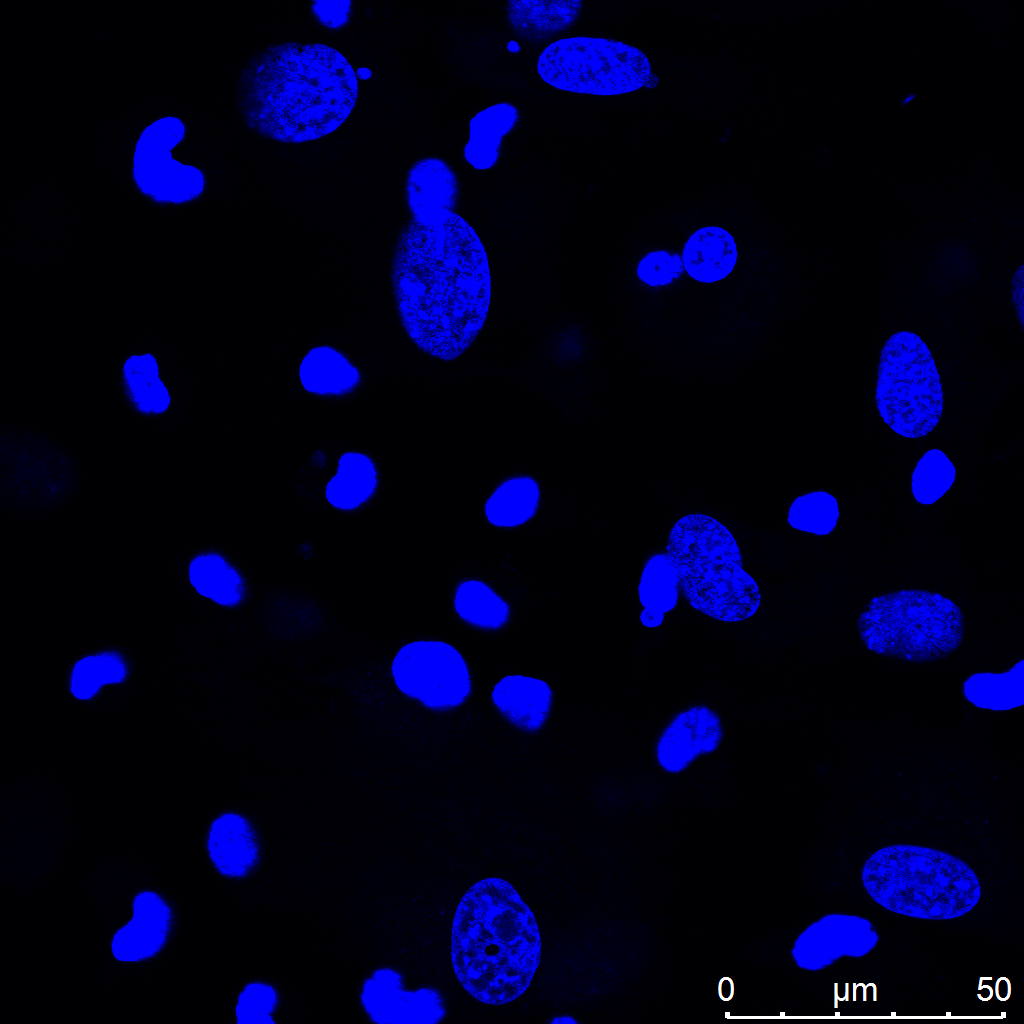

Supplement: Supplementary file 4 [file DataSheet4.zip › Figure4&5/immunofluorescence-nfatc1/DAPI+RANKL+20u.tif]

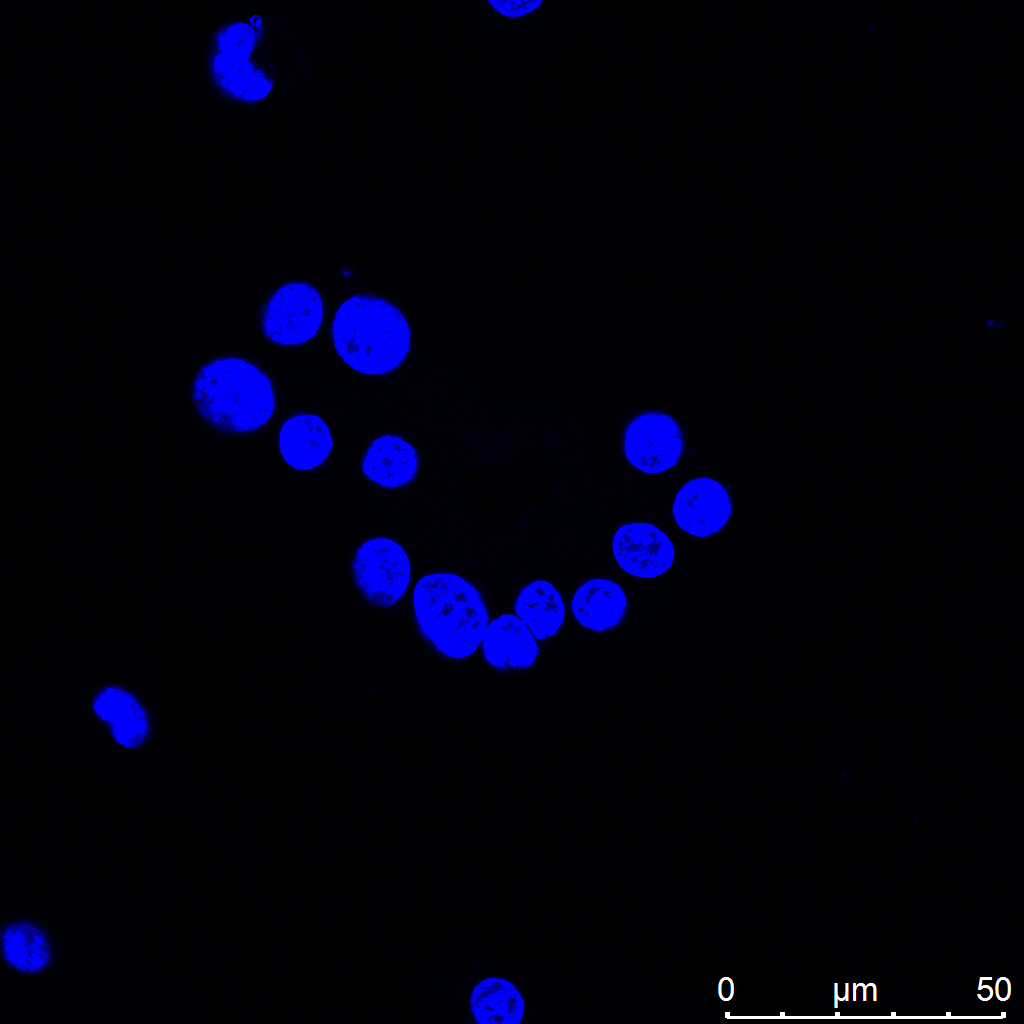

Supplement: Supplementary file 4 [file DataSheet4.zip › Figure4&5/immunofluorescence-nfatc1/DAPI+RANKL.tif]

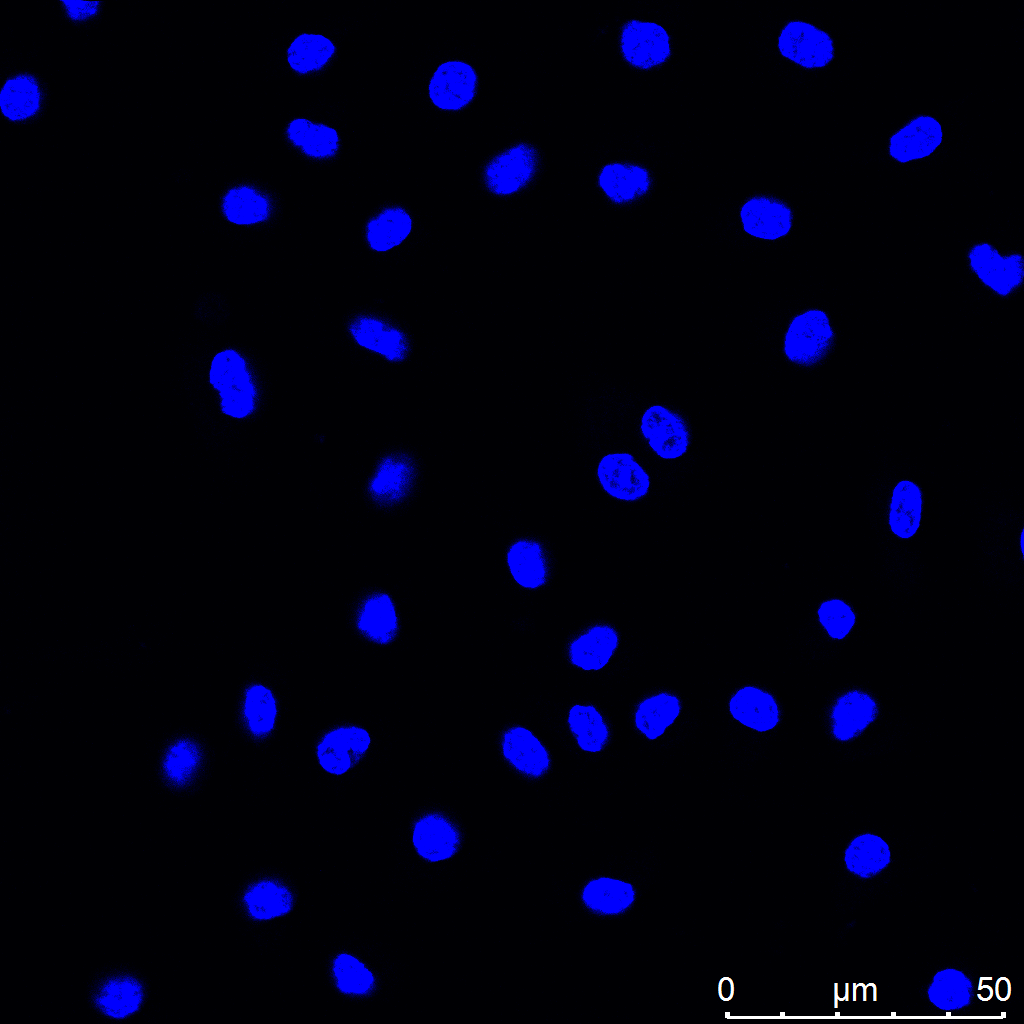

Supplement: Supplementary file 4 [file DataSheet4.zip › Figure4&5/immunofluorescence-nfatc1/DAPI-RANKL.tif]

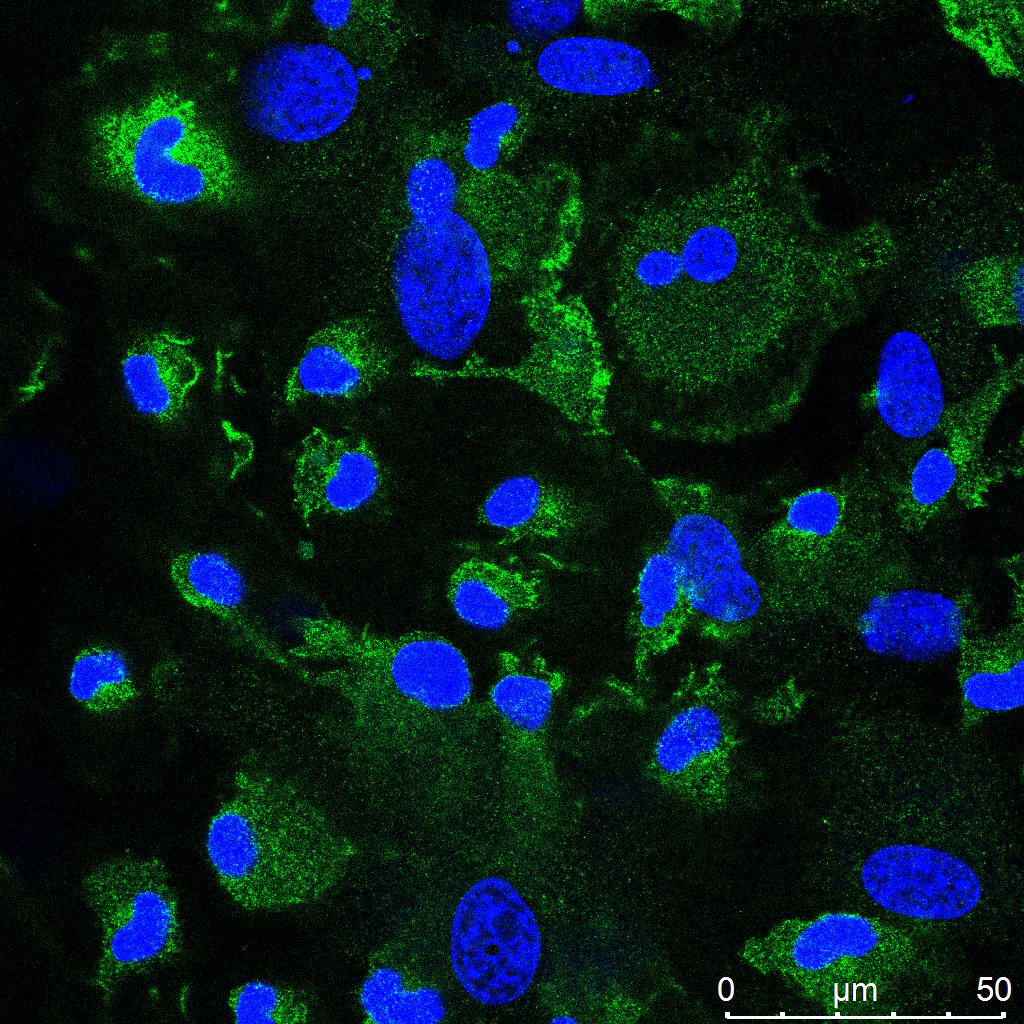

Supplement: Supplementary file 4 [file DataSheet4.zip › Figure4&5/immunofluorescence-nfatc1/Merge+RANKL+20um.tif]

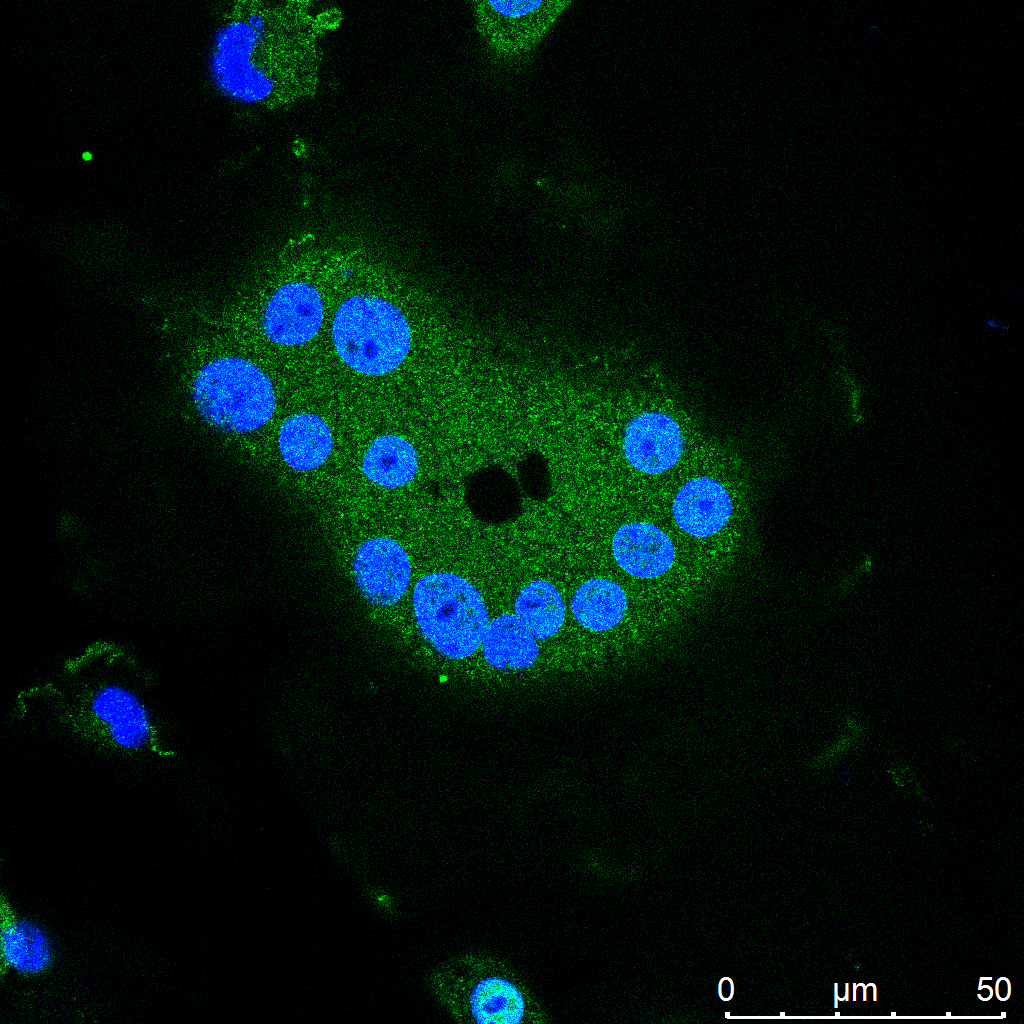

Supplement: Supplementary file 4 [file DataSheet4.zip › Figure4&5/immunofluorescence-nfatc1/Merge+RANKL.tif]

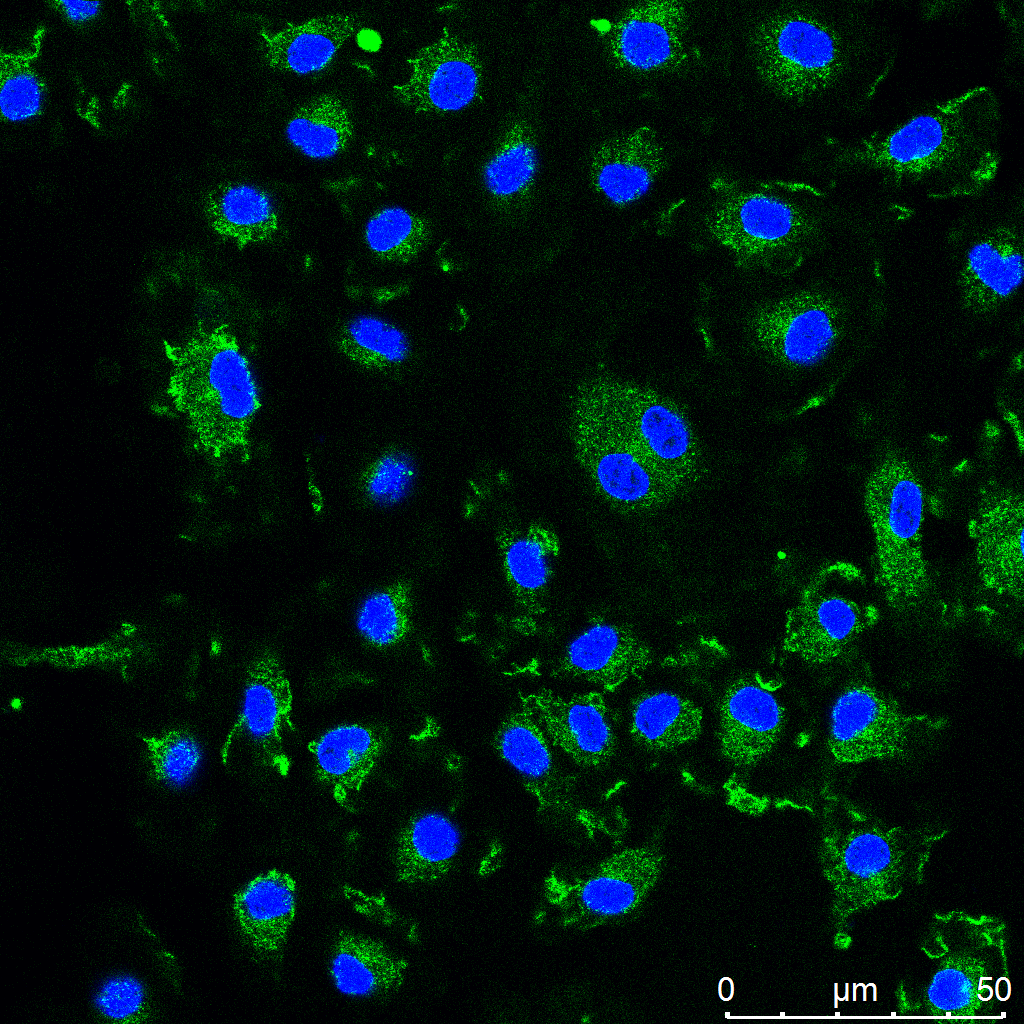

Supplement: Supplementary file 4 [file DataSheet4.zip › Figure4&5/immunofluorescence-nfatc1/Merge-RANKL.tif]

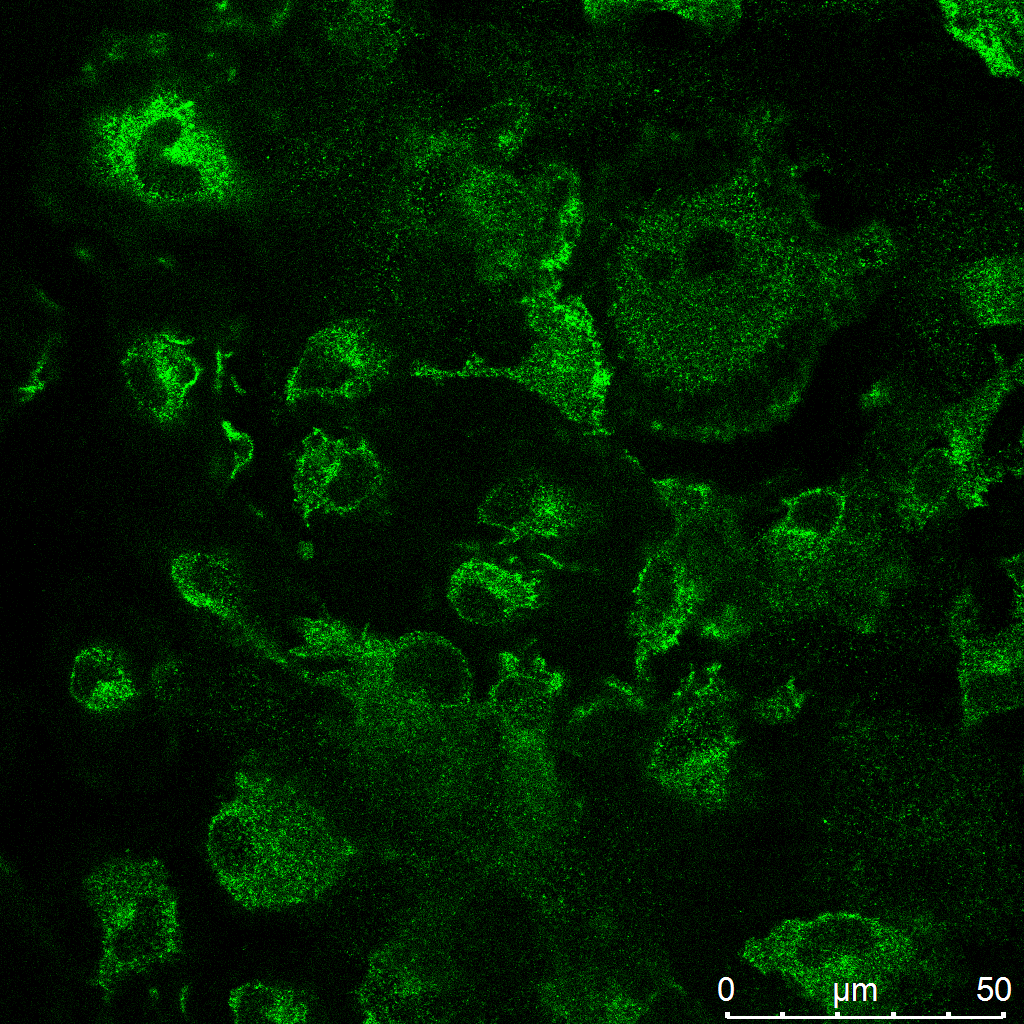

Supplement: Supplementary file 4 [file DataSheet4.zip › Figure4&5/immunofluorescence-nfatc1/NFATc1+RANKL+20um.tif]

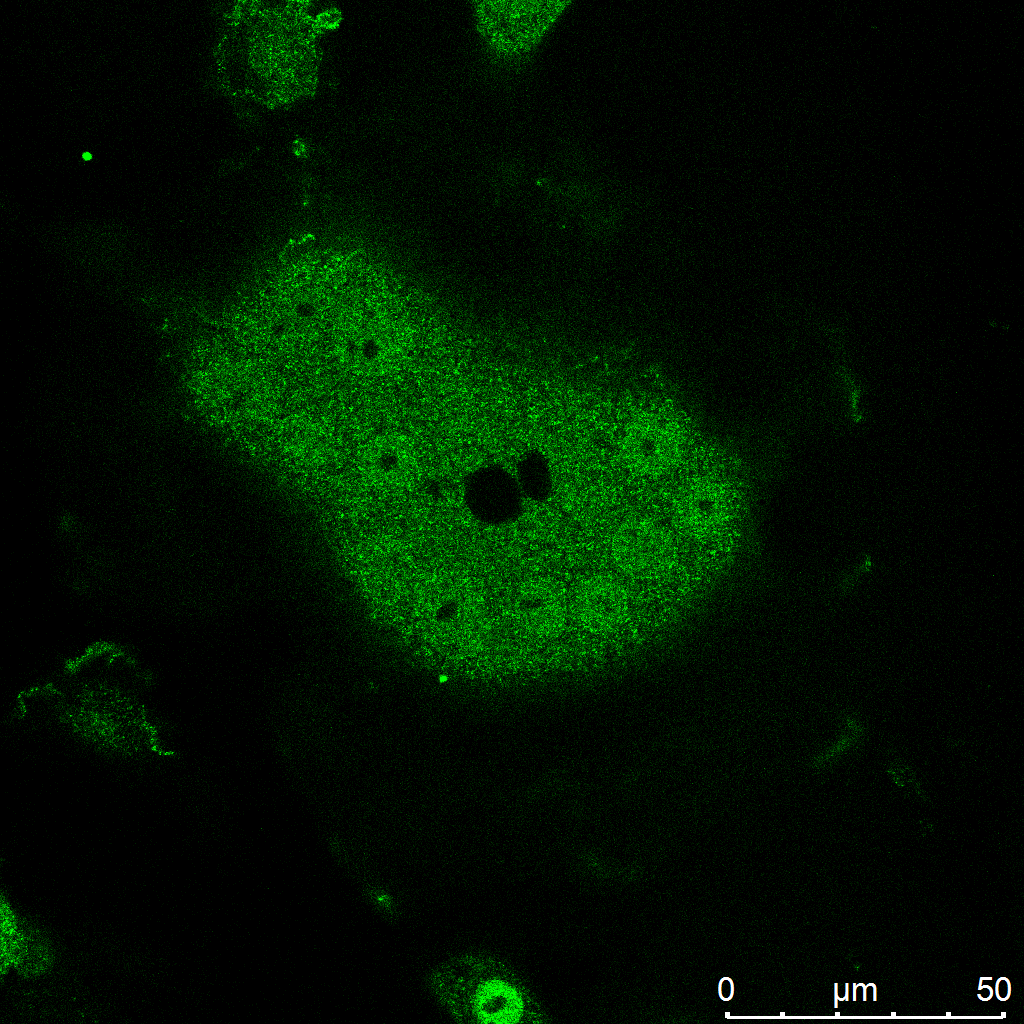

Supplement: Supplementary file 4 [file DataSheet4.zip › Figure4&5/immunofluorescence-nfatc1/NFATc1+RANKL.tif]

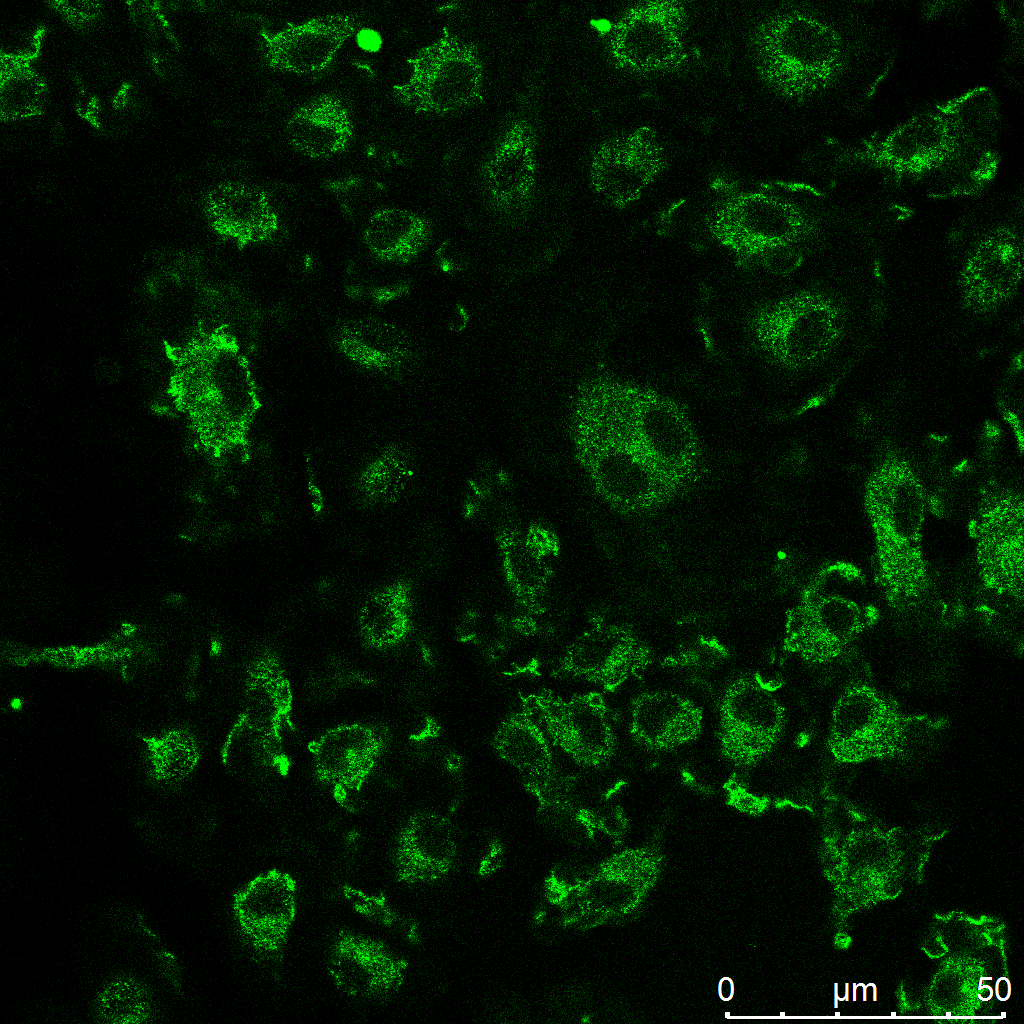

Supplement: Supplementary file 4 [file DataSheet4.zip › Figure4&5/immunofluorescence-nfatc1/NFATc1-RANKL+.tif]

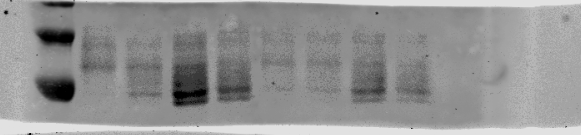

Supplement: Supplementary file 4 [file DataSheet4.zip › Figure4&5/westernblot/NFATc1.tif]

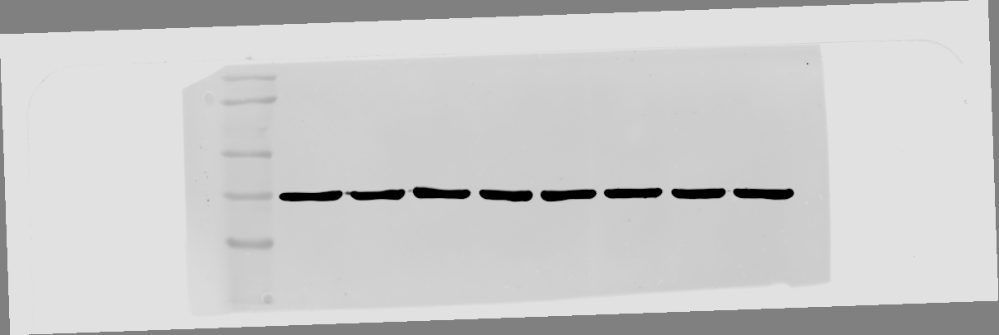

Supplement: Supplementary file 4 [file DataSheet4.zip › Figure4&5/westernblot/b-actin-2 .tif]

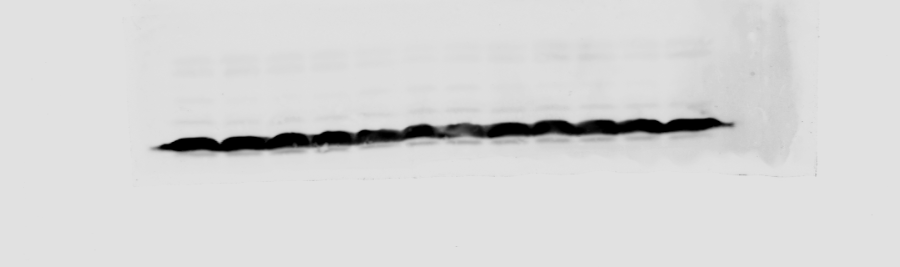

Supplement: Supplementary file 4 [file DataSheet4.zip › Figure4&5/westernblot/b-acting-1.tif]

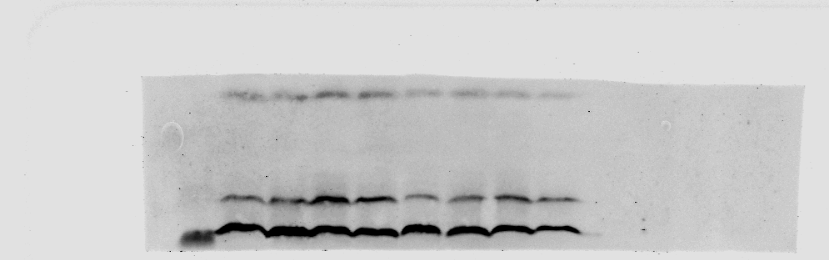

Supplement: Supplementary file 4 [file DataSheet4.zip › Figure4&5/westernblot/c-fos.tif]

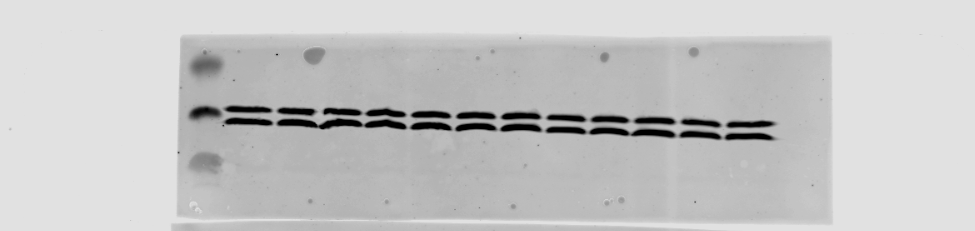

Supplement: Supplementary file 4 [file DataSheet4.zip › Figure4&5/westernblot/erk.tif]

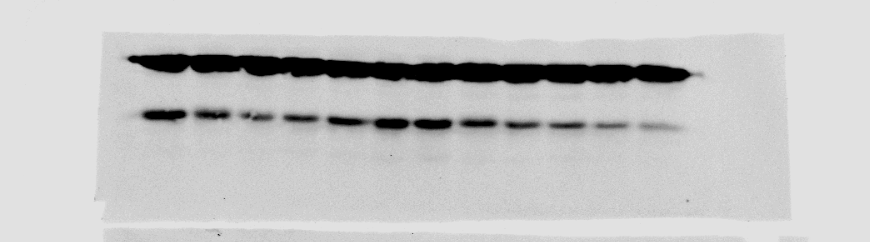

Supplement: Supplementary file 4 [file DataSheet4.zip › Figure4&5/westernblot/ikba.png]

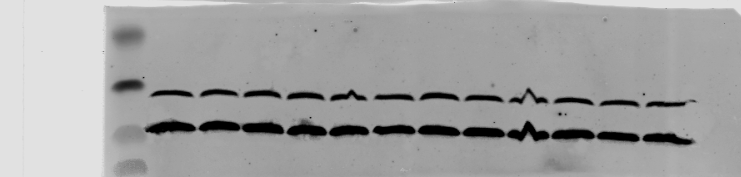

Supplement: Supplementary file 4 [file DataSheet4.zip › Figure4&5/westernblot/jnk.tif]

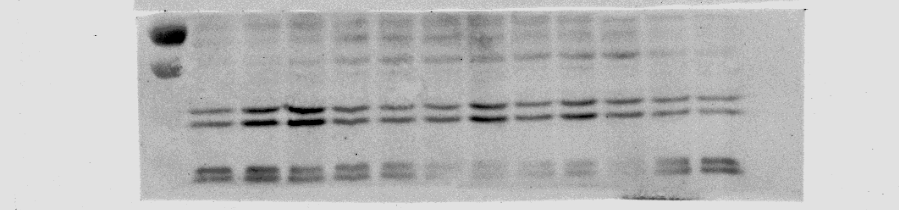

Supplement: Supplementary file 4 [file DataSheet4.zip › Figure4&5/westernblot/p-erk.tif]

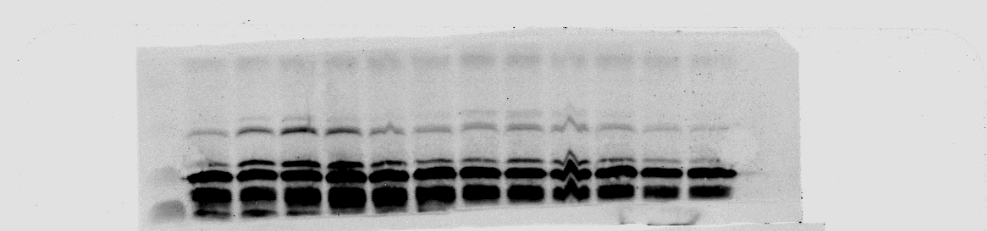

Supplement: Supplementary file 4 [file DataSheet4.zip › Figure4&5/westernblot/p-jnk.tif]

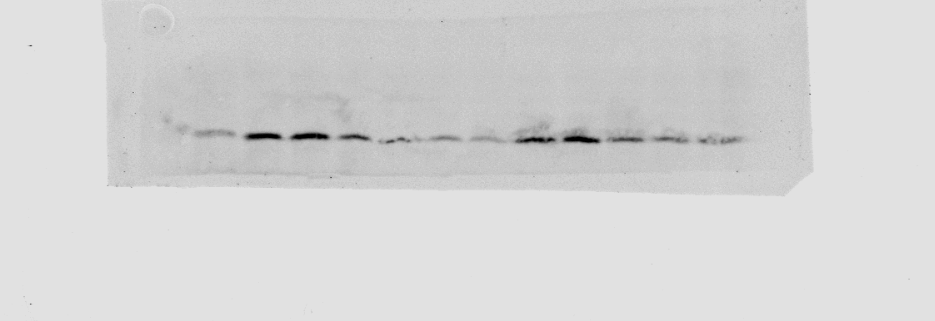

Supplement: Supplementary file 4 [file DataSheet4.zip › Figure4&5/westernblot/p-p38.tif]

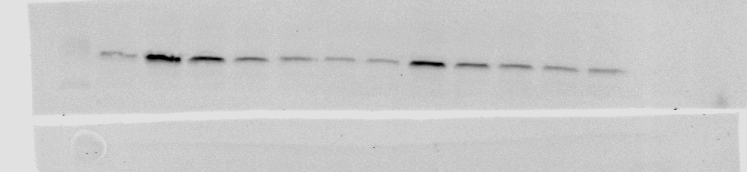

Supplement: Supplementary file 4 [file DataSheet4.zip › Figure4&5/westernblot/p-p65.tif]

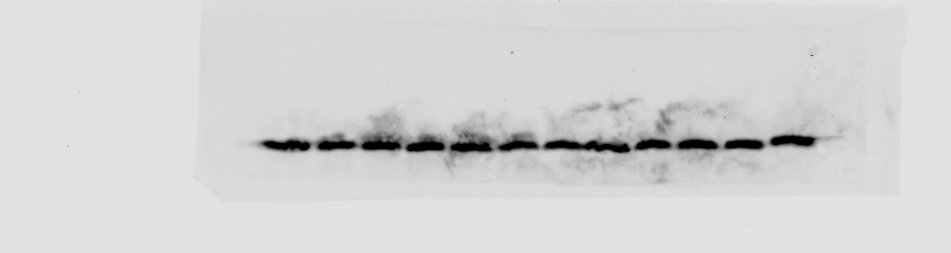

Supplement: Supplementary file 4 [file DataSheet4.zip › Figure4&5/westernblot/p38tif.tif]

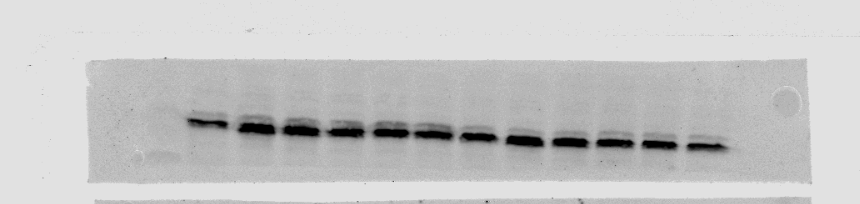

Supplement: Supplementary file 4 [file DataSheet4.zip › Figure4&5/westernblot/p65.tif]

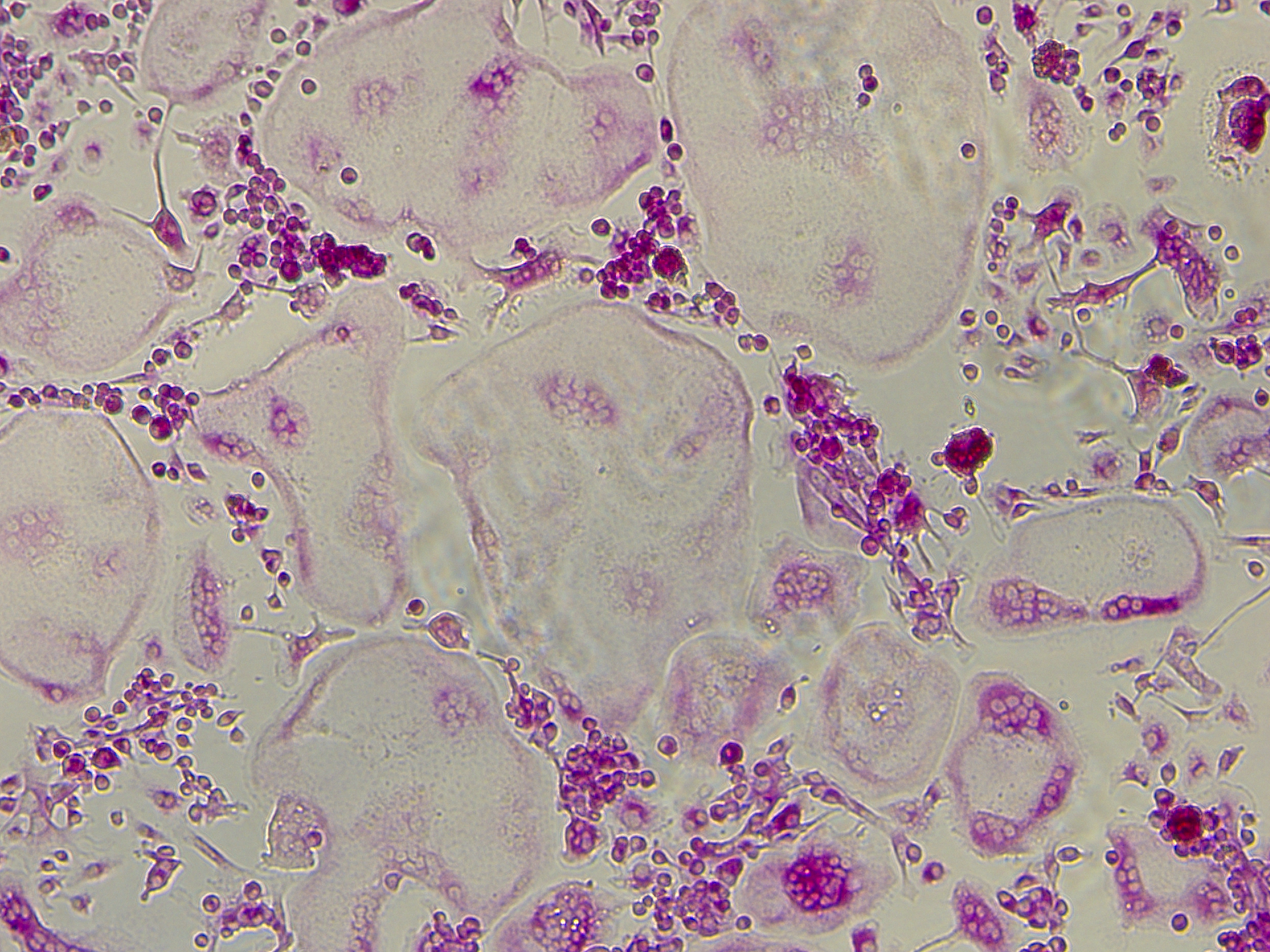

Supplement: Supplementary file 5 [file DataSheet1.ZIP › Figure1/TRAP STANING/0 um.tif]

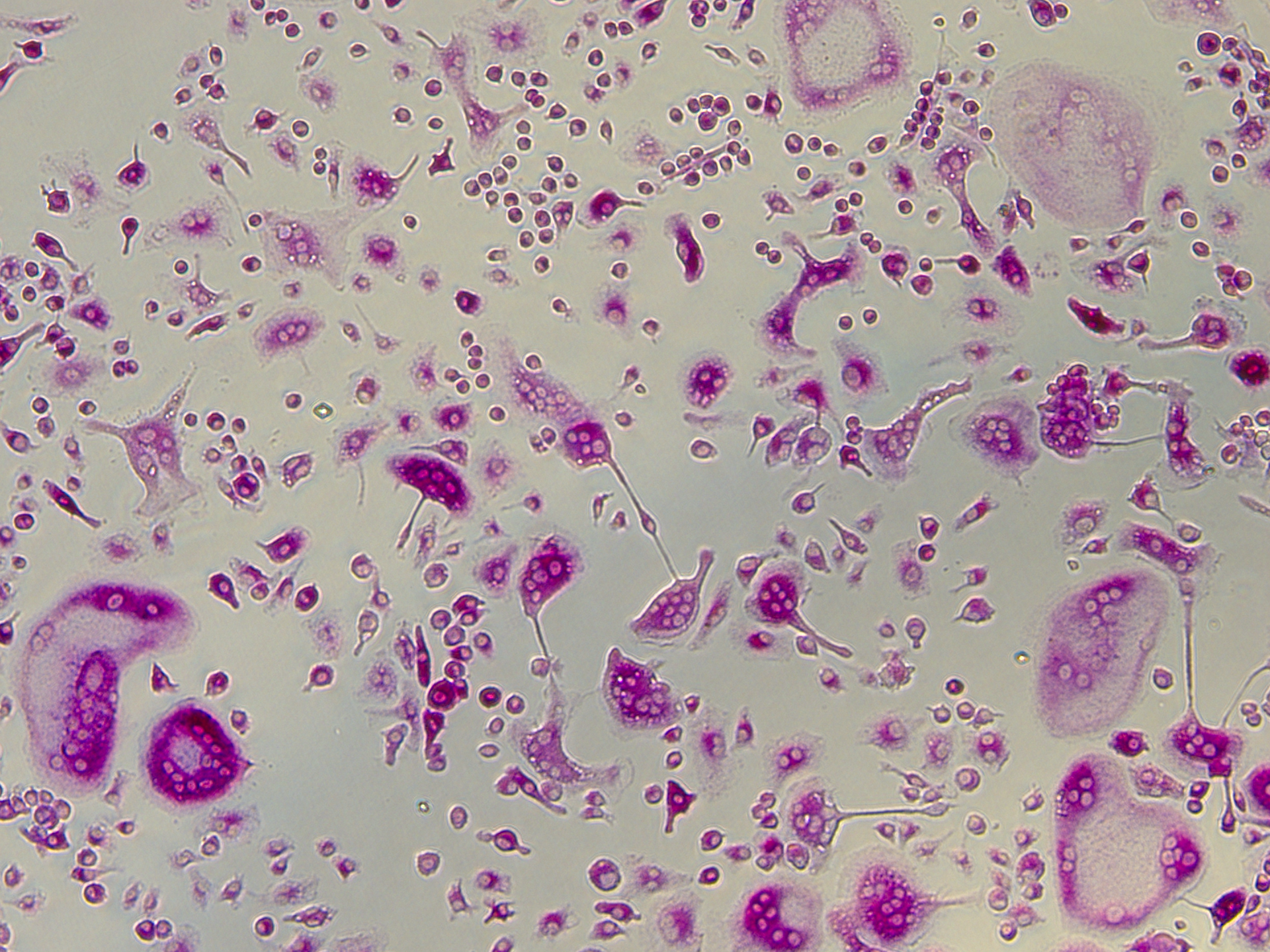

Supplement: Supplementary file 5 [file DataSheet1.ZIP › Figure1/TRAP STANING/10 um.tif]

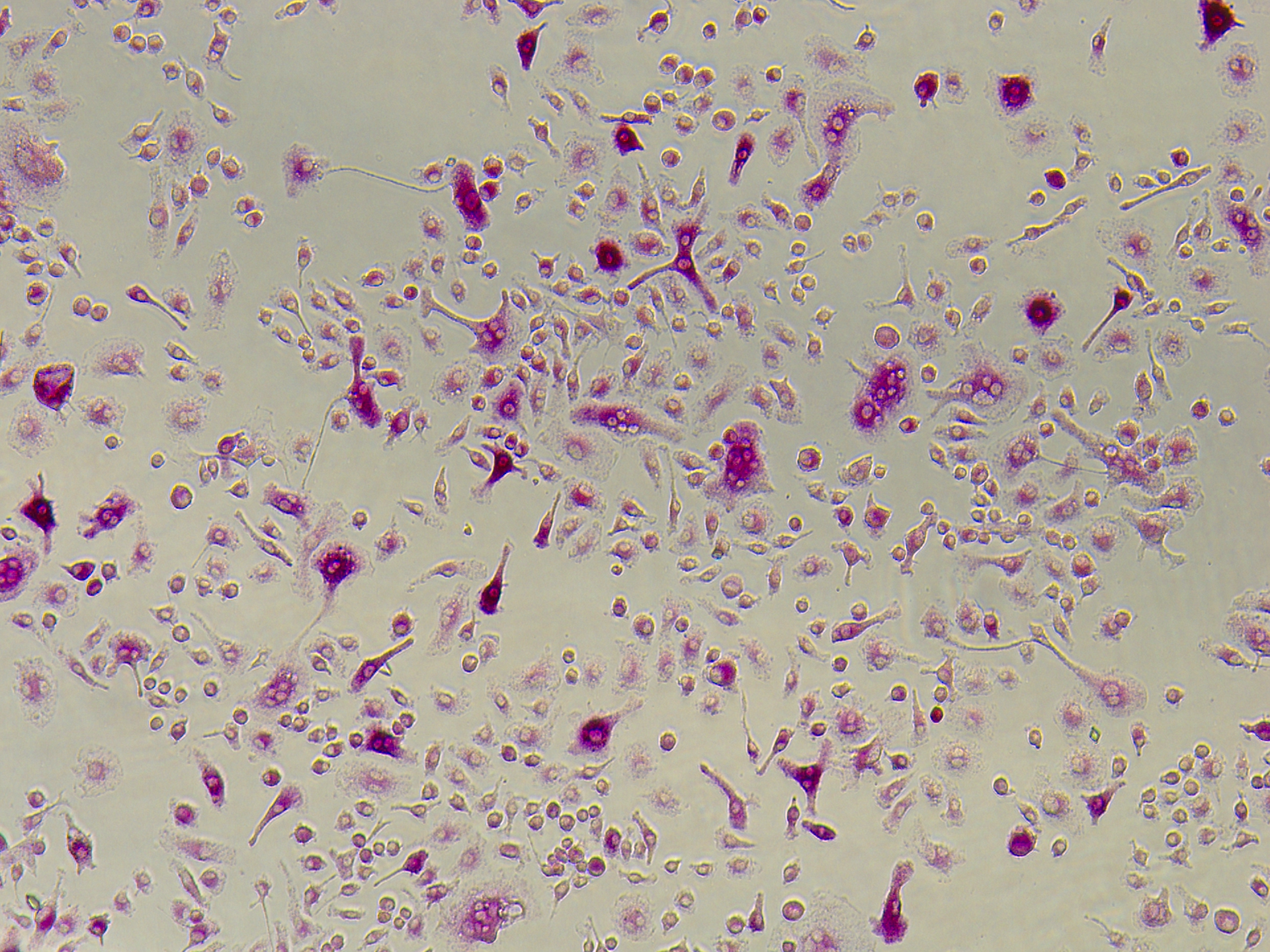

Supplement: Supplementary file 5 [file DataSheet1.ZIP › Figure1/TRAP STANING/20um.tif]

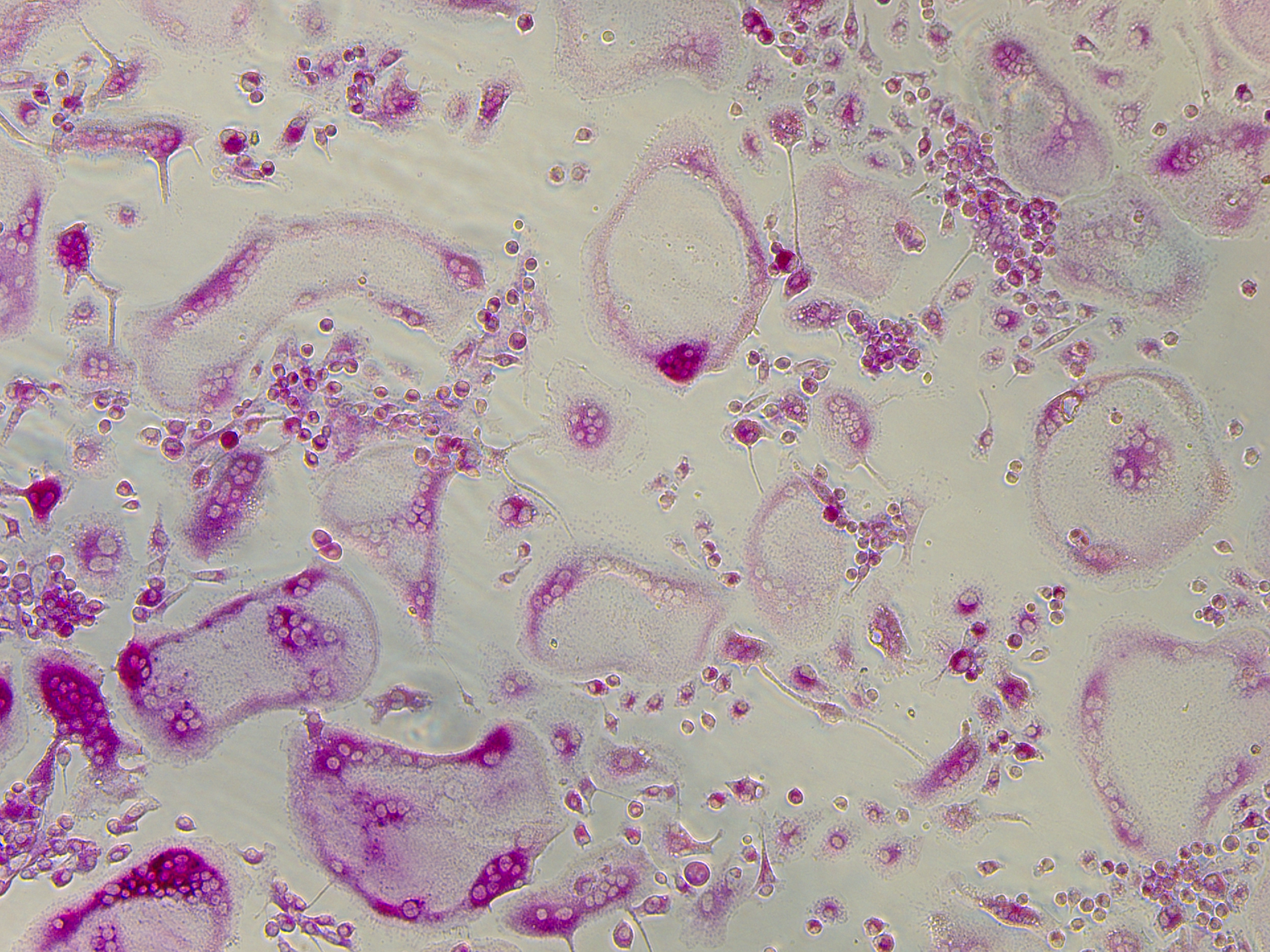

Supplement: Supplementary file 5 [file DataSheet1.ZIP › Figure1/TRAP STANING/5 um.tif]

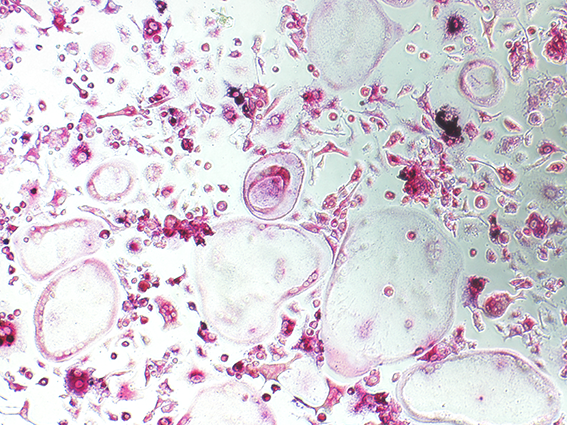

Supplement: Supplementary file 5 [file DataSheet1.ZIP › Figure1/TRAP STANING/time-period-trap/control.tif]

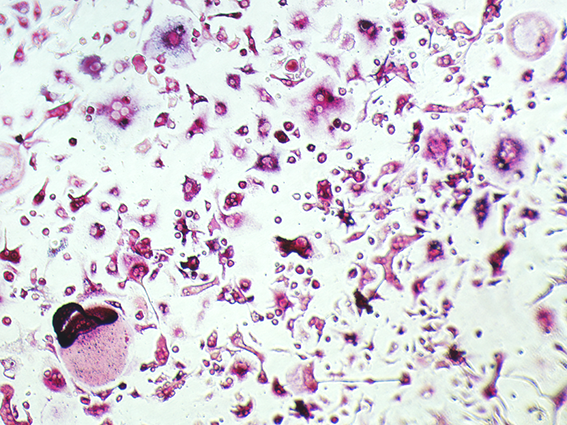

Supplement: Supplementary file 5 [file DataSheet1.ZIP › Figure1/TRAP STANING/time-period-trap/day-1-3.tif]

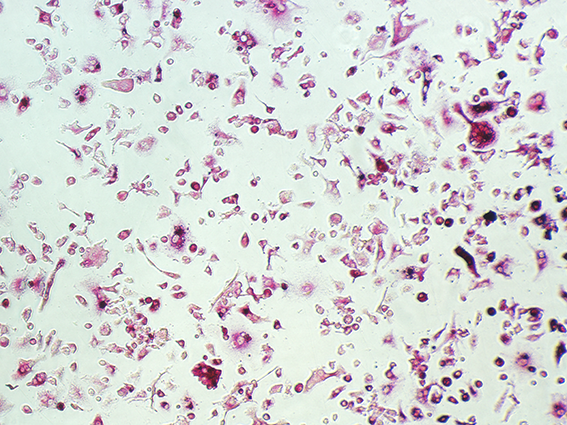

Supplement: Supplementary file 5 [file DataSheet1.ZIP › Figure1/TRAP STANING/time-period-trap/day3--5.tif]

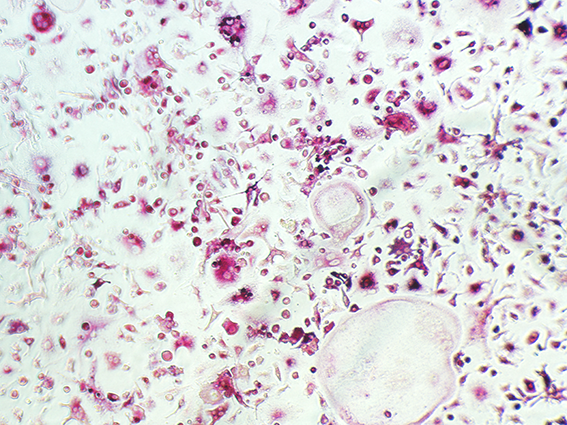

Supplement: Supplementary file 5 [file DataSheet1.ZIP › Figure1/TRAP STANING/time-period-trap/day5-6.tif]

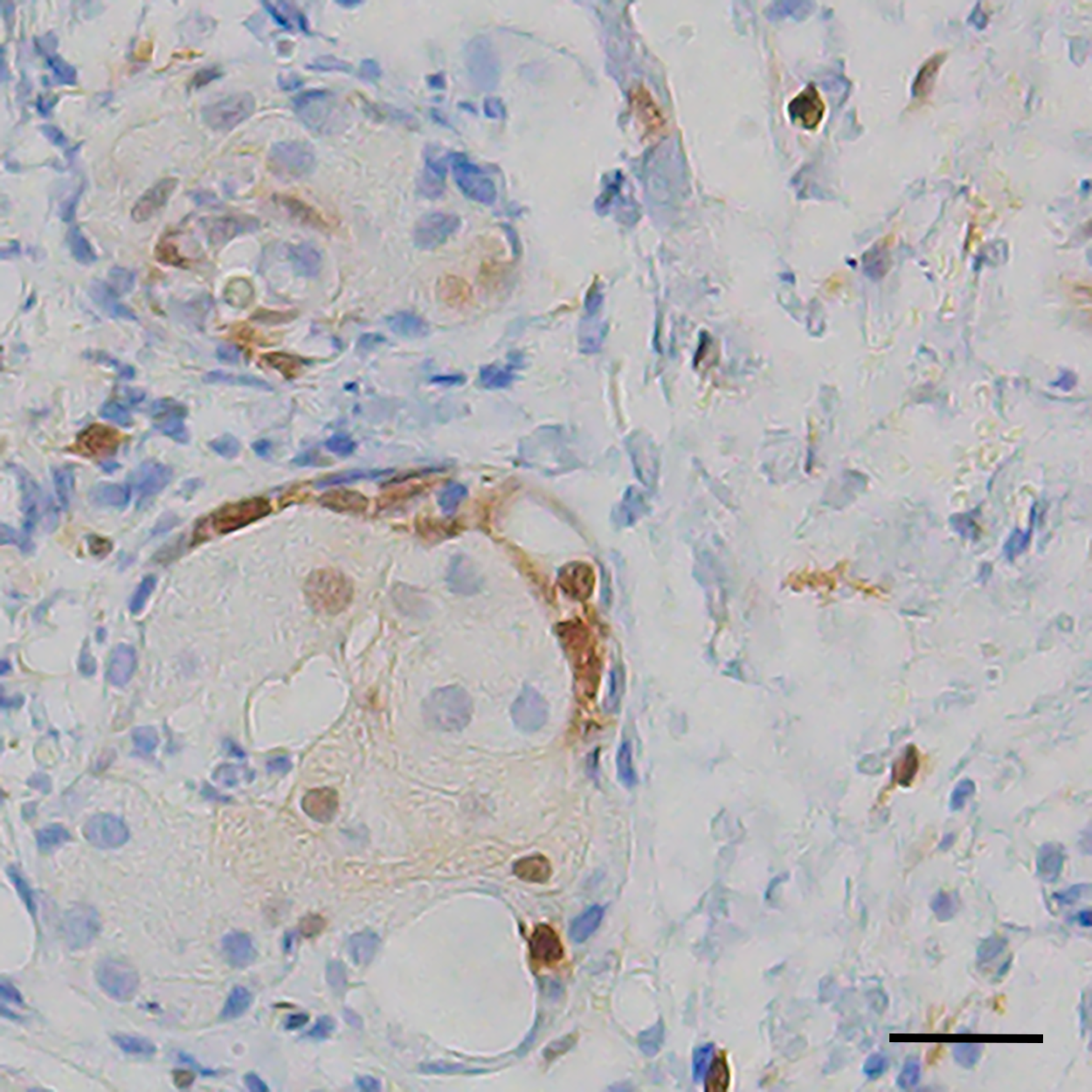

Supplement: Supplementary file 6 [file DataSheet6.ZIP › figure 7-IHC/High-dose.tif]

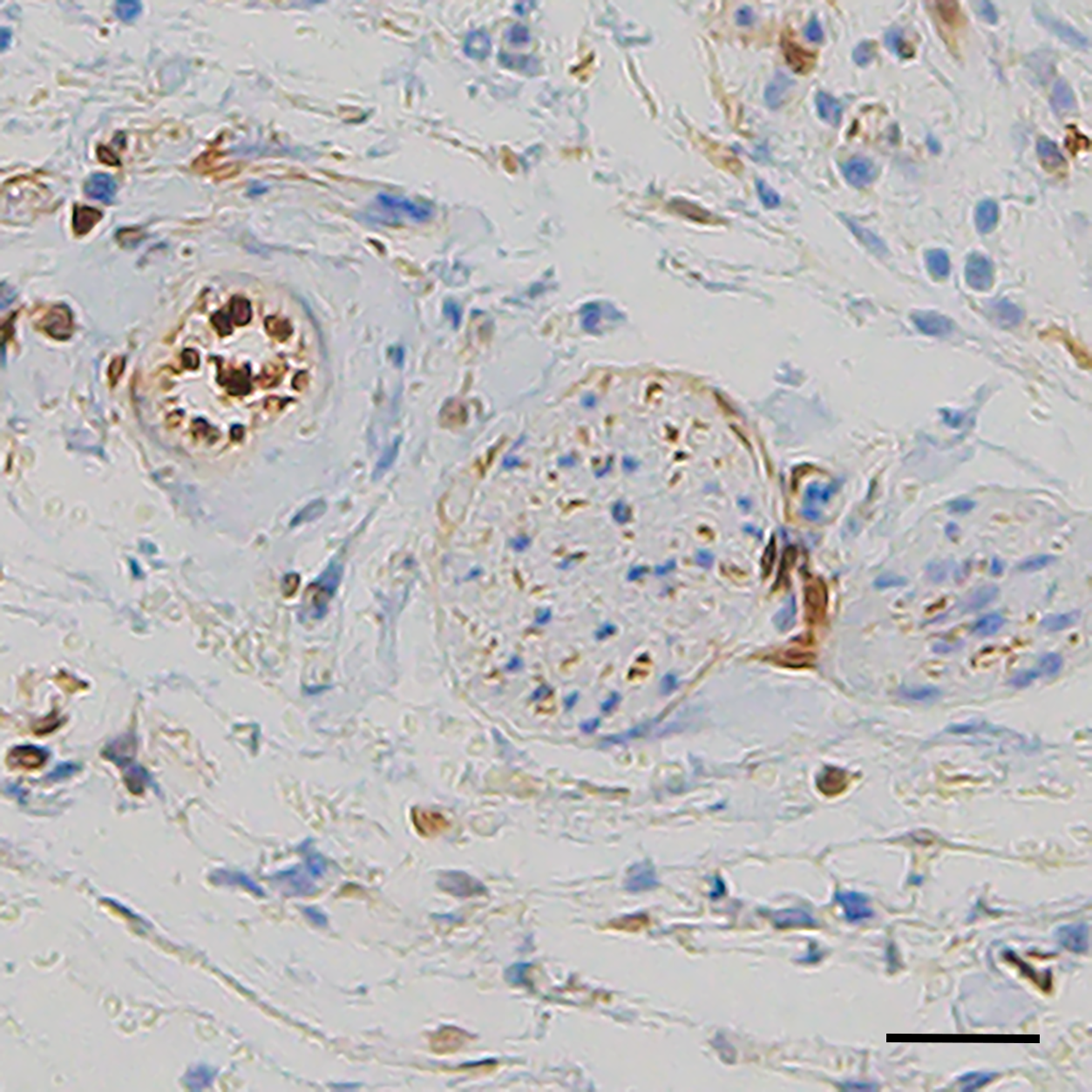

Supplement: Supplementary file 6 [file DataSheet6.ZIP › figure 7-IHC/Low-dose.tif]

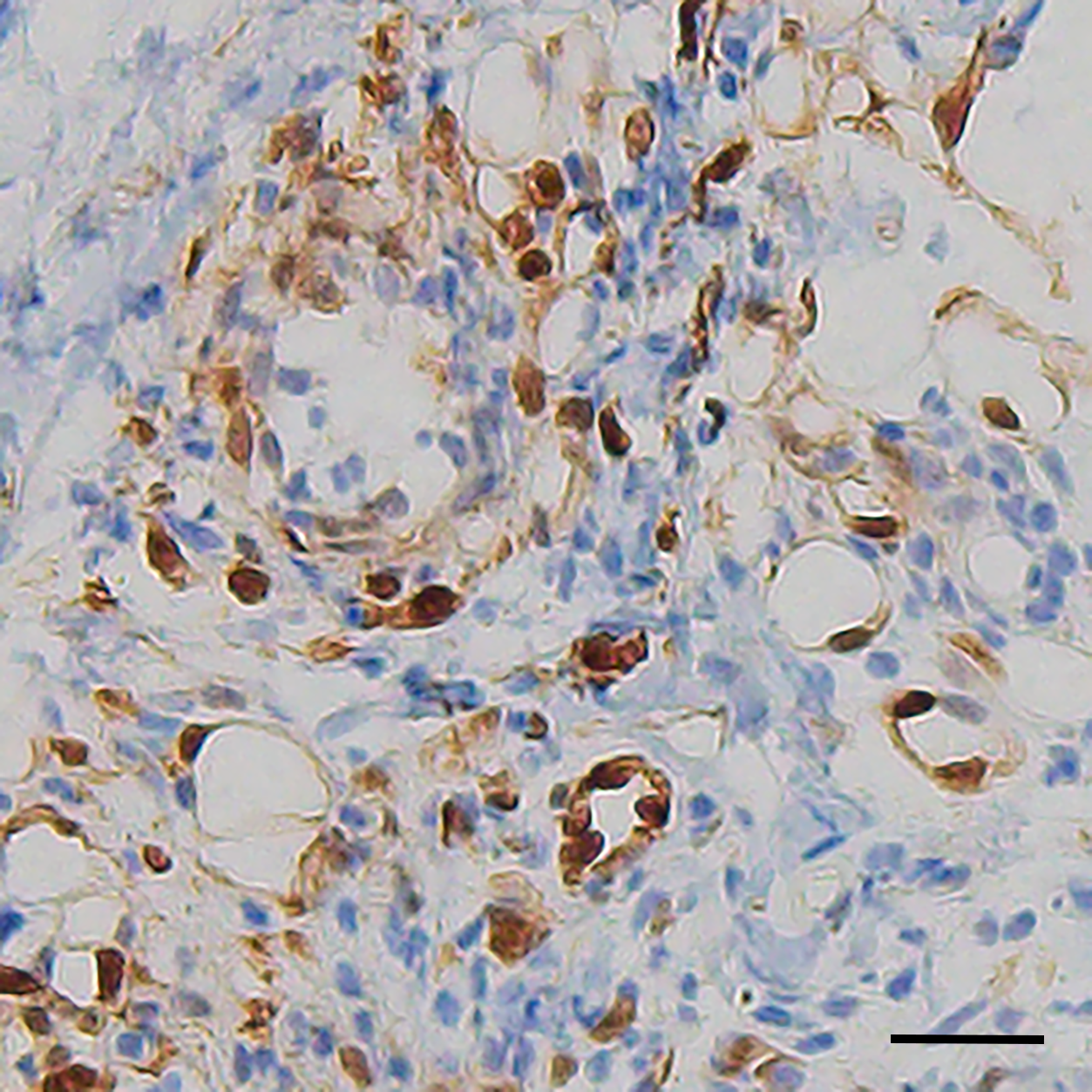

Supplement: Supplementary file 6 [file DataSheet6.ZIP › figure 7-IHC/Vechile.tif]

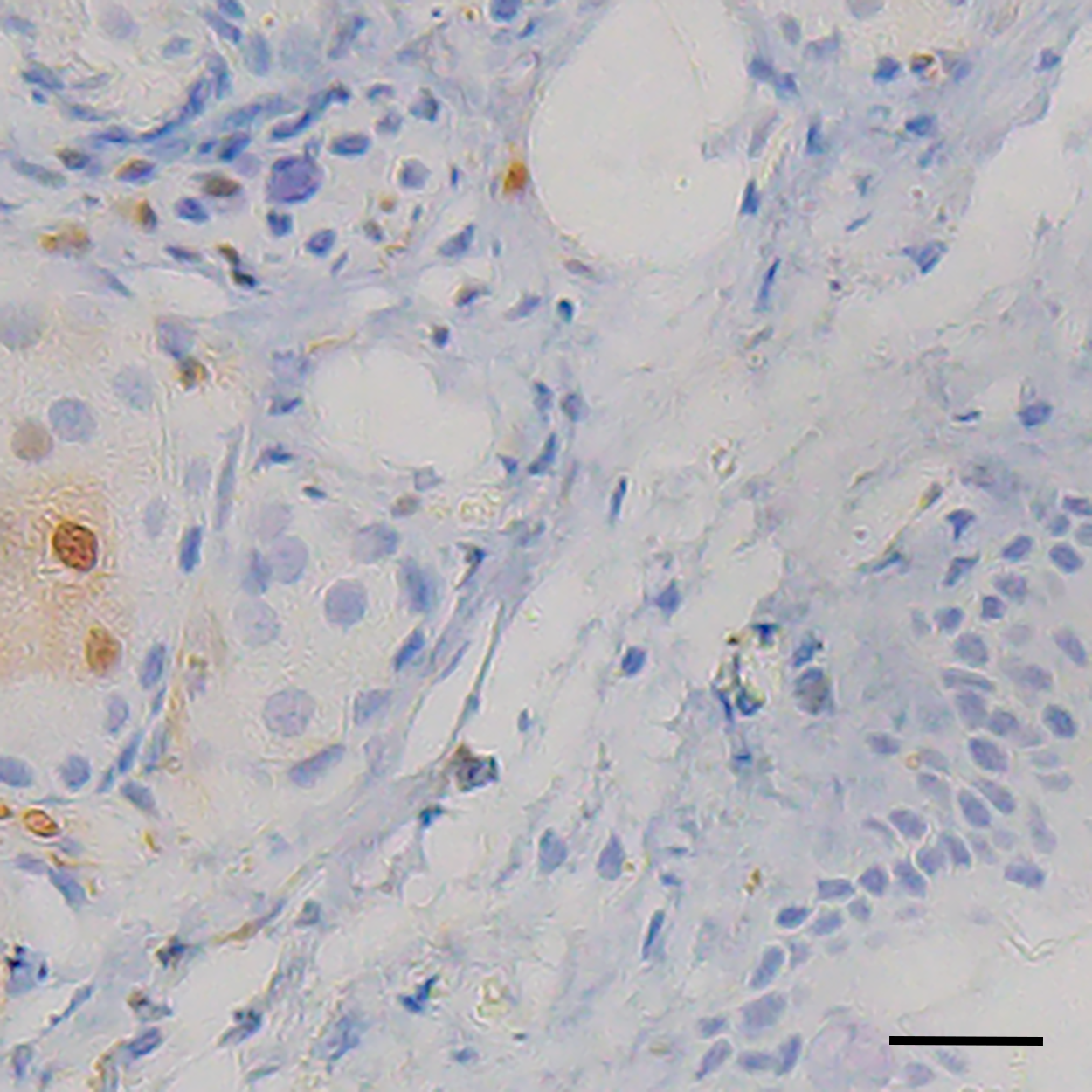

Supplement: Supplementary file 6 [file DataSheet6.ZIP › figure 7-IHC/sham.tif]

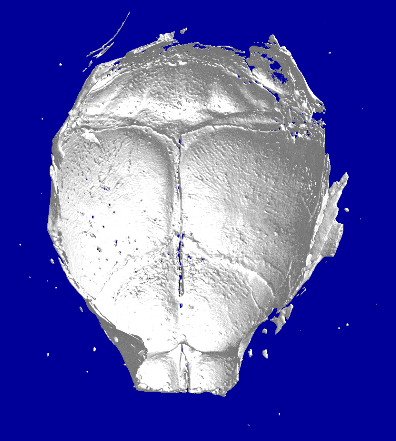

Supplement: Supplementary file 8 [file DataSheet5.ZIP › Figure6/CT/high-dose-1.tif]

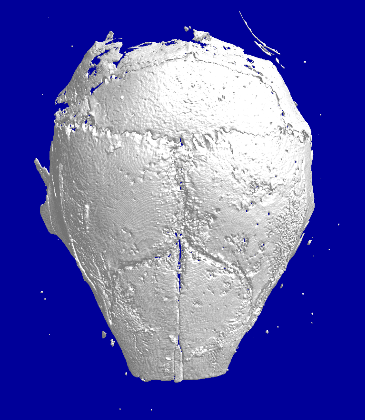

Supplement: Supplementary file 8 [file DataSheet5.ZIP › Figure6/CT/high-dose-2.tif]

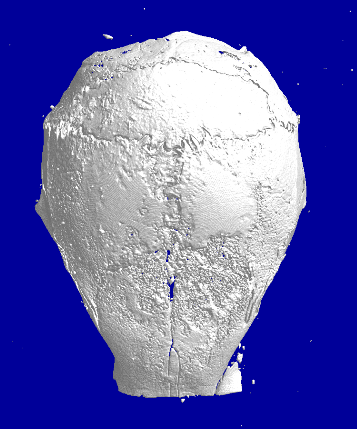

Supplement: Supplementary file 8 [file DataSheet5.ZIP › Figure6/CT/low-dose-1.tif]

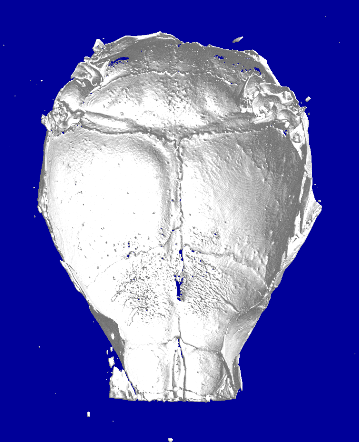

Supplement: Supplementary file 8 [file DataSheet5.ZIP › Figure6/CT/low-dose-2.tif]

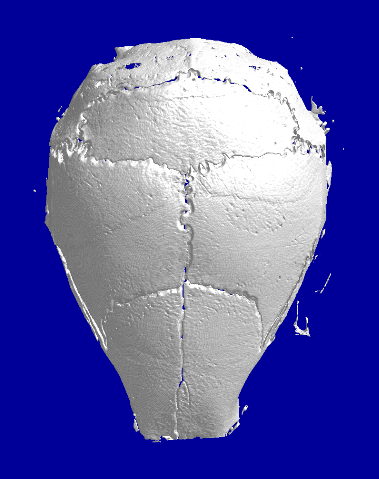

Supplement: Supplementary file 8 [file DataSheet5.ZIP › Figure6/CT/sham-1.tif]

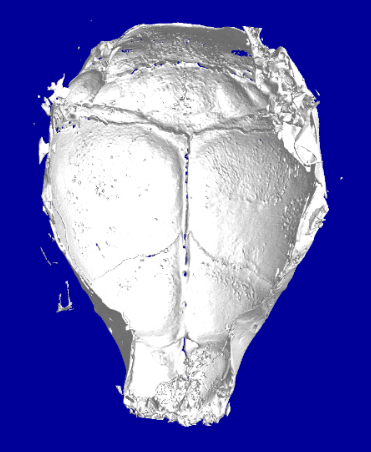

Supplement: Supplementary file 8 [file DataSheet5.ZIP › Figure6/CT/sham-2.tif]

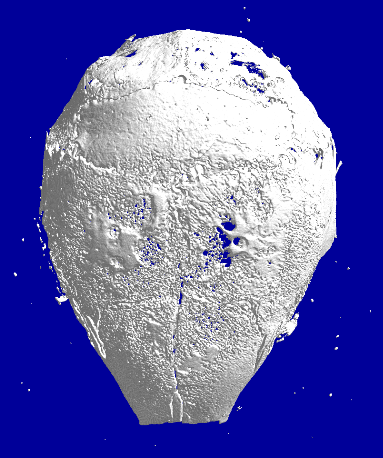

Supplement: Supplementary file 8 [file DataSheet5.ZIP › Figure6/CT/vehicle-1.tif]

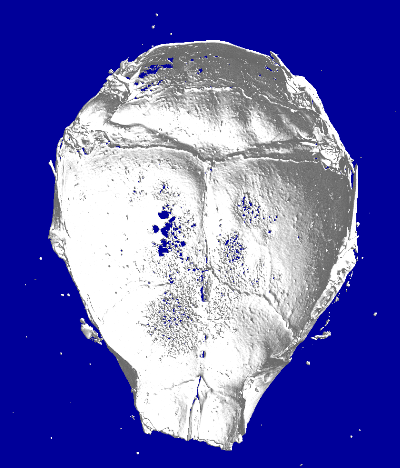

Supplement: Supplementary file 8 [file DataSheet5.ZIP › Figure6/CT/vehicle-2.tif]

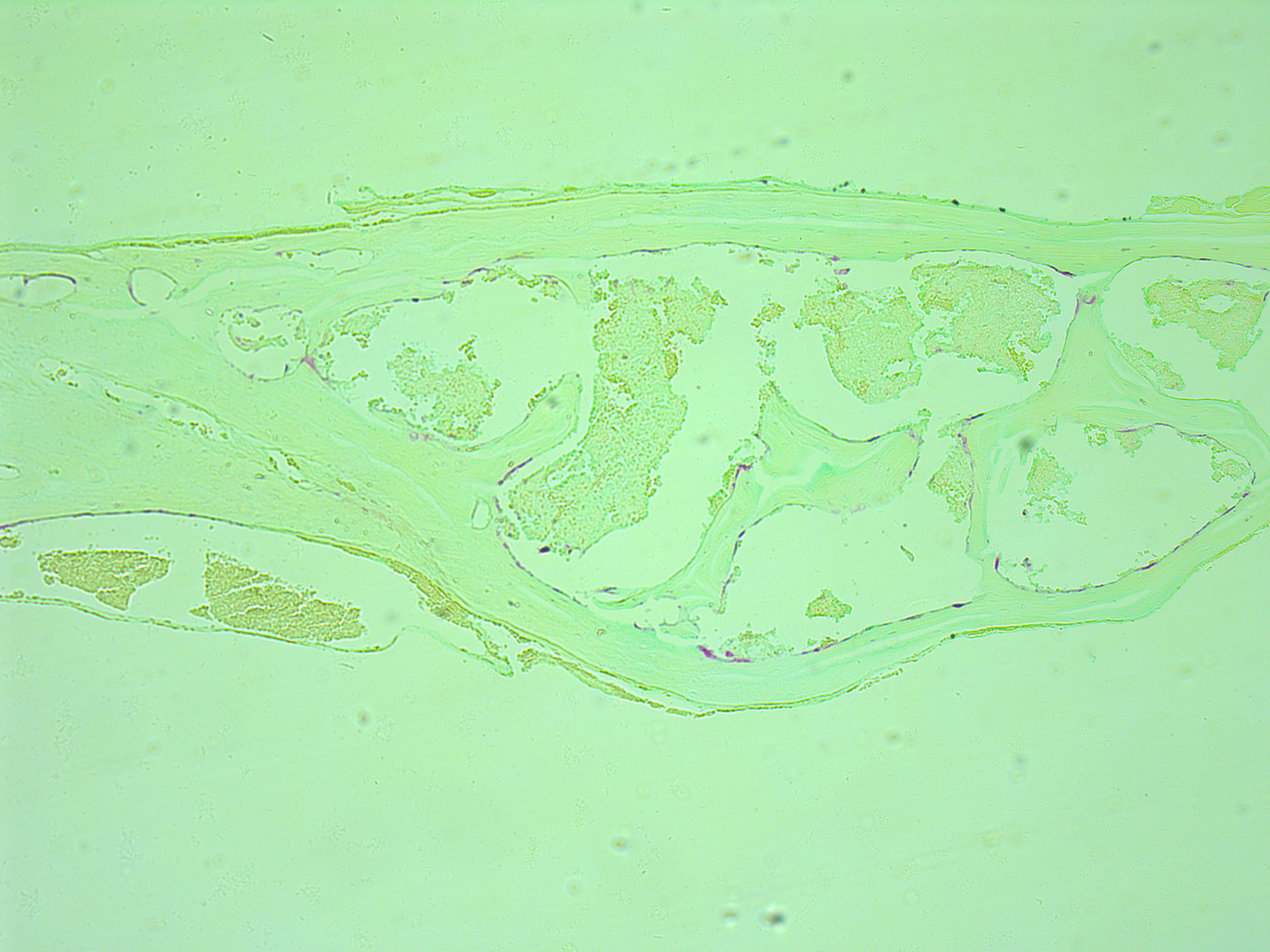

Supplement: Supplementary file 9 [file DataSheet7.ZIP › figure 7-100x-trap/LOW-dose 100X trap.tif]

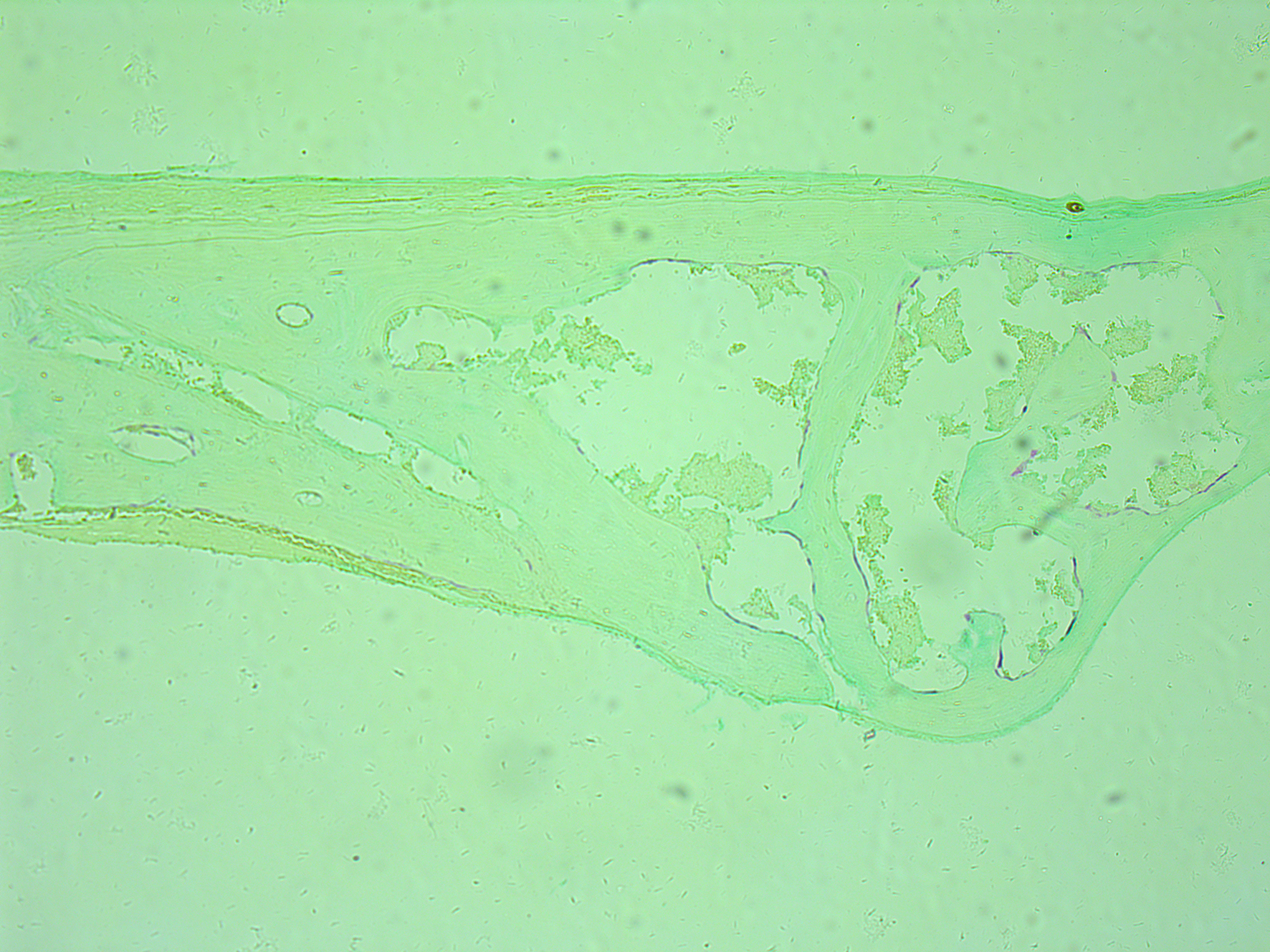

Supplement: Supplementary file 9 [file DataSheet7.ZIP › figure 7-100x-trap/high-dose 100X trap.tif]

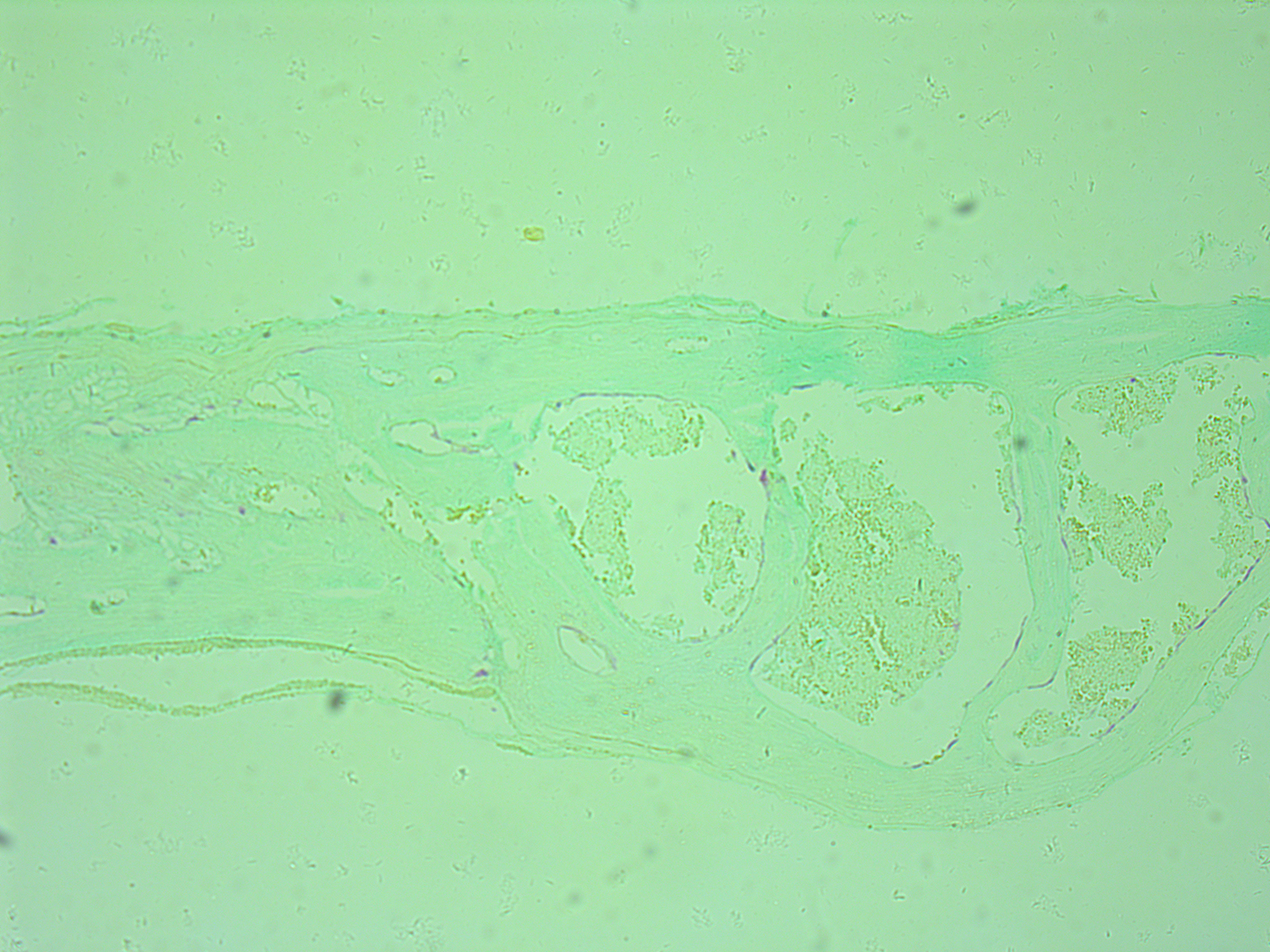

Supplement: Supplementary file 9 [file DataSheet7.ZIP › figure 7-100x-trap/sham100X TRAP.tif]

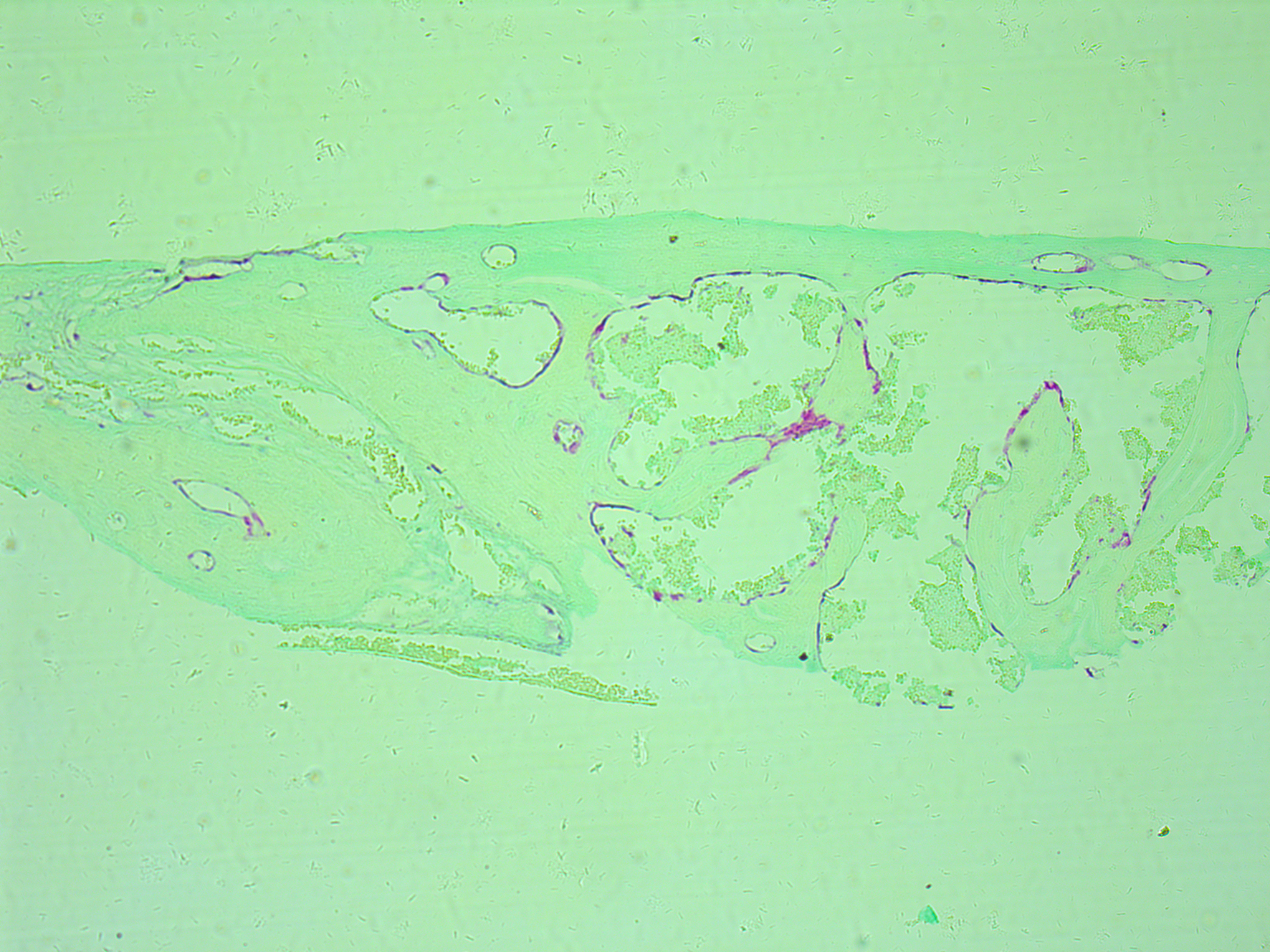

Supplement: Supplementary file 9 [file DataSheet7.ZIP › figure 7-100x-trap/vehicle 100X trap.tif]
